# Elevated atmospheric CO_2_ promoted speciation in mosquitoes (Diptera, Culicidae)

**DOI:** 10.1038/s42003-018-0191-7

**Published:** 2018-11-05

**Authors:** Chufei Tang, Katie E. Davis, Cyrille Delmer, Ding Yang, Matthew A. Wills

**Affiliations:** 1 Department of Entomology, College of Plant Protection, China Agricultural University, 100193 Beijing, China; 2 The Milner Centre for Evolution, Department of Biology & Biochemistry, University of Bath, Claverton Down, Bath, BA27AY UK; 3 Department of Biology, University of York, Heslington, York YO10 5DD UK

## Abstract

Mosquitoes are of great medical significance as vectors of deadly diseases. Despite this, little is known about their evolutionary history or how their present day diversity has been shaped. Within a phylogenetic framework, here we show a strong correlation between climate change and mosquito speciation rates: the first time to our knowledge such an effect has been demonstrated for insects. Information theory reveals that although climate change is correlated with mosquito evolution there are other important factors at play. We identify one such driver to be the rise of mammals, which are predominant hosts of Culicidae. Regardless of the precise mechanism, we demonstrate a strong historical association. This finding, taken in combination with projected rises in atmospheric CO_2_ from anthropogenic activity, has important implications for culicid vector distributions and abundance, and consequently for human health.

## Abstract (web summary)

Chufei Tang and Katie E. Davis et al. show that an elevated atmospheric CO_2_ promotes the speciation rates of mosquitoes. They demonstrate that climate change can expedite the evolution of mammalian disease vectors, potentially increasing vector−pathogen interactions and affecting human health.

## Introduction

The adverse effects of climate change upon global biodiversity are of increasing concern. Global warming is known to affect the diversity, abundance and distribution of animal and plant species^1^, but organisms respond in a wide variety of ways depending upon their ecology, physiology and life history. Similarly, elevated levels of CO_2_ may have effects on plants over and above those mediated by greenhouse warming^2^. Surprisingly, given the singular contribution of insects to global diversity, there have been few studies of the effects of temperature, and no direct studies of the effects of atmospheric CO_2_ levels on their species richness or rate of diversification^3^.

Mosquitoes (Diptera, Culicidae) include many of the most problematic insect pests, and are of particular medical significance. The adult females of many species require a blood meal from a vertebrate, which is located using CO_2_^4^ and other chemical cues, before producing eggs^5^. Mosquitoes vector filarial nematodes, a variety of bacterial infections (including tularaemia) and numerous viral diseases (including zika, yellow fever, dengue fever, chikungunya, West Nile virus and other arboviruses)^6^. Specially, Malaria, which is caused by species of the alveolate *Plasmodium*, is only vectored to humans by *Anopheles*, a genus of Culicidae^6,7^. Effective treatments are lacking for many of these infections. An estimated one million people die each year as a result of pathogens transmitted via these bites^7^.

Mosquitoes have extremely wide environmental tolerances and a nearly ubiquitous geographical distribution, being present on all major landmasses except Antarctica and Iceland. Nevertheless, changes in climate and land use on ecological timescales can variously expand or fragment their distribution patterns^8^, raising consequent concerns for human health. Here we ask whether such ecological responses leave a signature in the pattern of clade diversification throughout the deep time history of the group. In particular, we investigate speciation as a potential correlate of both global mean temperature and atmospheric CO_2_ concentration over the last 195 million years. We also attempt to identify the mechanism by which climate change has driven speciation in mosquitoes.

Diversification through time is often quantified using simple taxonomic methods. Species (or higher taxa as proxies) are treated as independent units of diversity, and their numbers are tallied within successive time bins^9^. Such approaches have been used very effectively when testing the correlation between climate and biological diversification for predominantly extinct groups with a rich fossil record^10^. Mosquitoes, by contrast, have a rich extant diversity but a fragmentary fossil record concentrated in a few sites of exceptional preservation^11^. In such cases, more sophisticated phylogenetic methods can be implemented to model diversification rates through time using Bayesian inference^12,13^. These approaches require large and inclusive trees, which are not always available for the most speciose groups. Large trees can be inferred from supermatrices: concatenations and reanalyses of all available morphological and molecular character data. This is a painstaking and time-consuming process. Large blocks of missing data can create analytical problems^13^ and further data collection may ultimately be necessary^14^. Moreover, the best methods for analysing one data partition may not be optimal for all^15^. The alternative and usually more tractable approach entails synthesising a supertree from all of the published phylogenies for the group. This is most often achieved by encoding the structure of the source trees using group inclusion characters and resolving conflicts using parsimony: formally, Matrix Representation Parsimony (MRP)^10,16–18^. Our supertree is synthesised from 550 source trees published between 1981 and 2014. We time-calibrate this using those fossil dates in which we have greatest confidence, enabling us to model rates of speciation across the last 195 million years. We demonstrate statistically how these rates correlated with changes in atmospheric CO_2_ and global temperature. We also show that there was a direct transfer of information from the speciation of mammals to the speciation of mosquitoes. However, despite significant correlation, there was an additional missing driver acting to transfer information from climate to the biota.

## Results

### The most inclusive phylogeny of mosquitoes

**Fig. 1:**
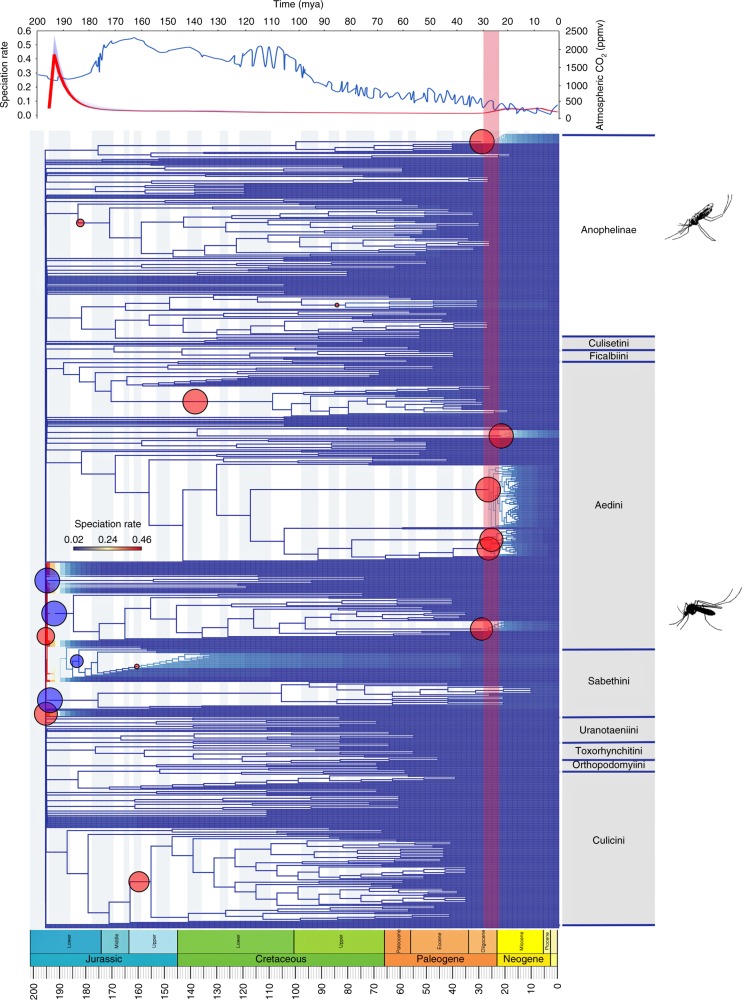
Phylogeny of Culicidae, time-calibrated against the international stratigraphic timescale (shown in the bottom of the figure). Rates of speciation are indicated via coloured branches (dark blue denotes low rates; dark red denotes high rates). Significant rate shifts are shown as circles: blue symbols represent decreases and red circles represent increases. The size of these same circles denotes the marginal shift probability: larger symbols indicate higher probabilities. Anophelinae constitute the sister group of Culicinae (grey vertical bar). Silhouettes illustrate typical resting postures of flies from the two subfamilies. The top panel shows atmospheric CO_2_ concentration (blue) and the mean speciation rate of Culicidae through time (red). The red vertical bar marks the most recent significant speciation rate increase (30–24 mya), which coincided with the parallel radiation of many mammal groups as potential hosts

Our supertree includes 1000 leaves (a coincidentally round number), equivalent to 28% of described mosquito species from nearly 80% of the genera identified by Reinert et al.^19^, and is therefore the most inclusive phylogeny ever assembled for the group. A small number (about 1%) of species were recovered in anomalous subfamilies, but this phenomenon has been noted in other MRP supertrees^10,16–18^, and is a function of both the sparsity of taxon sampling in some source trees and the degree of overlap between them^18^. The two subfamilies, Culicinae and Anophelinae, resolved as sister clades (Fig. 1). Relationships at the generic and deeper levels were broadly consistent with those proposed by Harbach^20^ based on morphological characters, but greatly expanded upon that author’s taxon sample (28 genera within two subfamilies). Relationships within Anophelinae were well resolved and consistent with previous studies. Tribes within Culicinae were mostly recovered as clades, with the exceptions of Aedeomyiini, Orthopodomyiini and Ficalbiini (which are poorly sampled: Fig. 1; Supplementary Fig. 1).

**Fig. 2:**
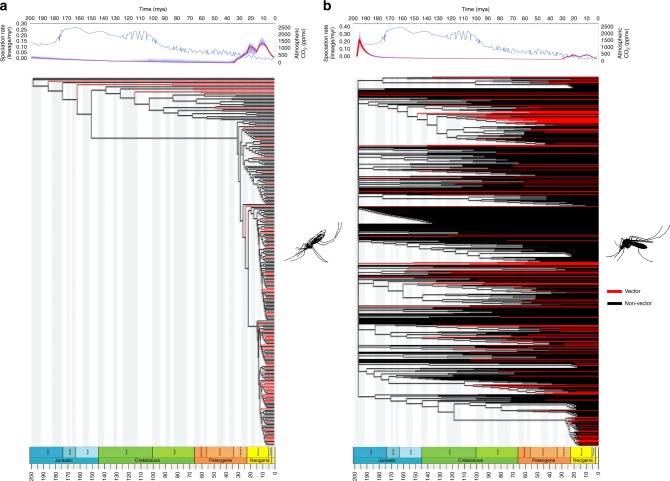
Expanded trees of the two subfamilies of Culicidae. In both of the two panels, vectors are marked in red and non-vectors are marked in black. The panel above each tree shows atmospheric CO_2_ concentration (blue) and the mean speciation rate through time (red). Silhouettes illustrate typical resting postures of flies from the two subfamilies. **a** Anophelinae and **b** Culicinae

Relationships within the traditional *Aedes* (Culicinae) are the most problematic part of the tree, and were the subject of greatest controversy hitherto^19,20^, reflecting conflicts within the source trees. Human vector species are indicated on our supertree (Fig. 2a, b), and although there is specificity of pathogens to vectors near the tips of the phylogeny, there is increasing liability in these interactions progressively deeper into the tree. Closely related vectors can therefore convey distantly related pathogens, and vice versa.

### Divergence times

The two constituent subfamilies of Culicidae, namely Culicinae and Anophelinae, diverged around 195 mya (million years ago), and were sister clades in our supertree. Most extant genera diverged before 130 mya, with the exception of *Anopheles* and *Ochlerotatus*, which were estimated to have first appeared around 51 and 48 mya respectively. These originations were in the immediate wake of the Palaeocene-Eocene Thermal Maximum (PETM) 56 mya. At this time CO_2_ levels were re-equilibrating after record high levels, but both ocean and air temperatures were nonetheless elevated^21^. Atmospheric CO_2_ levels are currently increasing at their fastest rate since the PETM^22^, and this may similarly stimulate rates of speciation across the group.

### Overall shifts in speciation rates

**Fig. 3:**
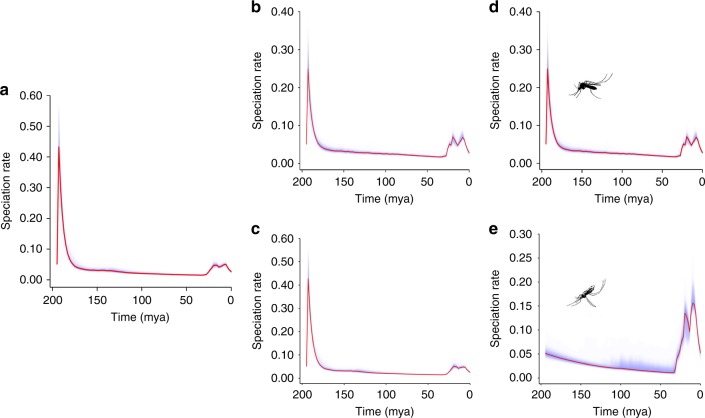
The mean speciation rates through time of the phylogenies of mosquitoes. **a** Entire tree (Culicidae), **b** vector species only, **c** non-vector species only, **d** Culicinae, and **e** Anophelinae

Mean speciation rates throughout the tree were inferred in BAMM^12^ (9000 samples of rate−time data pairs: a figure determined empirically from previous studies^10^) and mean speciation rates for major groups have been illustrated through time (Fig. 3). Overall mean rates of speciation were significantly faster in Anophelinae than Culicinae. There were also three major rate shifts across the entire Culicidae: one just before 194 mya (a brief and transient increase), a second at 194 mya (a significant decline, continuing to 180 mya) and a third between 30 and 24 mya (a more modest acceleration) (Figs. 1 and 3a). Mean speciation rates within the entire Culicidae remained low between 180 and 30 mya (Fig. 3a). All significant rate shifts across all Culicidae, as well as within vector and non-vector species considered in isolation, are tabulated (Supplementary Table 1). Vector and non-vector species both had trends of mean speciation rate similar to those for the entire Culicidae (Fig. 3b, c). The Anophelinae underwent only the most recent (30–24 mya) increase in rate: largely a function of the rapid radiation of *Anopheles* (Fig. 3d). Culicinae underwent patterns of speciation rate change similar to those in the entire Culicidae (Fig. 3e). The most recent (30–24 mya) speciation rate increase coincided with the evolution of C_3_ grasses, the opening up of savannah habitats^23^, the late Oligocene warming, and the parallel speciation of several groups of ruminants^24^, rodents^25^ and other mammal clades that may have increased the availability of host species (Fig. 1).

Our speciation rate analyses also demonstrated more rapid speciation amongst vector species than non-vectors, and rates of speciation in Anophelinae twice as fast as those in Culicinae (Supplementary Table 2). In response to recent criticisms of BAMM^12^, we verified our speciation rate estimates by calculating diversification rates for Anophelinae and Culicidae using a fossilised birth−death model based on molecular data and implemented in BEAST 2^26^. The diversification rate of Anophelinae was estimated at 0.0553 lineage per myr (million years), which is still distinctly higher than the diversification rate of Culicidae (0.037 lineage per myr). To further investigate any putative relationship between speciation rate and partitions of our data (vector/non-vector, Anophelinae/Culicinae), phylogenetic generalised least squares regression tests were used. Speciation rate was significantly correlated with the partition of subfamily (*p* < 0.001), but not with the partition into vector and non-vector species (*p* = 0.7) as in our BAMM analyses.

### Speciation rates increase with rising atmospheric CO_2_, and with rising temperature

Our detrended cross-correlation analysis (DCCA) tests^27^ demonstrated that rising CO_2_ concentration correlated positively and very significantly (*p* < 2.2e−16 in all cases) with increasing rates of speciation across the Culicidae, as well as in all of our subgroups (Anophelinae and Culicinae, vectors and non-vectors). This correlation was stronger for the non-vectors than for the vectors, and stronger for the Culicinae than for the Anophelinae. CO_2_ is a potent greenhouse gas, and its atmospheric concentration is correlated with mean global temperatures over a variety of timescales^28^. Global warming is the obvious mechanism by which elevated CO_2_ levels might cause increases in the rate of speciation, but our results are equivocal in this regard. While increasing rates of speciation correlate significantly with increasing temperature (*p* < 2.2e−16 in all cases), the correlation of speciation with CO_2_ level is stronger still (Table 1). Moreover, the correlation between speciation rate and temperature is stronger for vectors than for non-vectors, and stronger for the Culicinae than for the Anophelinae. Hence, at least some of the effects of CO_2_ must be mediated by mechanisms other than greenhouse warming.

**Table 1:** Correlations between the mean speciation rate for different groups of Culicidae and either temperature or atmospheric CO_2_ concentration

Category	Temperature	CO_2_ concentration
	DCCA	DCCA
Culicidae	0.2389 ± 0.0003	0.72156 ± 0.0005
Vector	0.2960 ± 0.0070	0.68103 ± 0.0005
Non-vector	0.2474 ± 0.0004	0.72141 ± 0.0005
Culicinae	0.2686 ± 0.0004	0.70705 ± 0.0006
Anophelinae	0.1615 ± 0.0030	0.31791 ± 0.0004

*p*-value < 2.2e−16 in all cases

### Information transfer

Calculating information flow from palaeo-temperature and atmospheric CO_2_ to the mosquito speciation rate time series reveals a misinformation signal (i.e., a negative value for TE) for both analyses (temperature = −0.780; CO_2_ = −1.950). This indicates that global palaeo-temperature and atmospheric CO_2_ both drove mosquito speciation, but via unknown mechanisms. By contrast, we find information transfer from the mammal speciation rate time series to the mosquito speciation rate time series (0.222), but not in the reverse direction (0.013). However, there remains a misinformation signal from both climate variables (palaeo-temperature = −0.704; CO_2_ = −1.861) to mammal speciation, implicating one or more additional processes^29^. The flow of information from mammals to mosquitoes shows that the rise of mammals is one factor underlying mosquito diversification. However, the precise mechanism by which climate change drives speciation in both mammals and mosquitoes remains unknown. All results were significant at the 95% confidence interval (Supplementary Table 3).

## Discussion

Climate change over macro-evolutionary time scales has long been known to drive biotic turnover^30^. Erwin^31^ theorised that the positive association between biodiversity and global temperature seen at the spatial scale (e.g., the latitudinal diversity gradient) would predict a positive relationship between biodiversity and global temperatures through time. The results of some previous work support this^32,33^, whilst others have found evidence to the contrary^10,34^. For mosquitoes, we find that global warming and elevated atmospheric CO_2_ in particular are strongly correlated with increased speciation rates. We also find that more than one mechanism is required to fully explain the effect of past climate change on biotic evolution.

Our finding that mammal speciation is linked to mosquito speciation is unsurprising given that mammals are a predominant source of food for many species of mosquitoes^35^. Anophelinae, in particular, exhibit distinctive and diverse behaviours and a high degree of host specificity^36^. *Anopheles*, which constitutes most species of Anophelinae, is renowned for feeding on both humans and domesticated animals, and is the sole vector of human malarial parasites. The speciation rate in Anophelinae is relatively high, but the correlation between this rate and both atmospheric CO_2_ and temperature is comparatively low. Hence, speciation in Anophelinae may also have been driven, at least in part, by its relatively high degree of host specificity and the evolution of mammals.

We note in this context that female mosquitoes in search of a blood meal use levels of CO_2_ as the initial cue to locate potential human or animal hosts. However, this stimulus is less effective as the ambient level of CO_2_ increases, since the differential between the host and its environment becomes less marked^37^. At closer quarters, a diversity of other chemical cues become more important for host selection, including lactic acid, saccharides, blood group and other antigens, as well as a variety of additional volatile odours^38^. It is well recorded that some people are more prone to mosquito bites than others, though the mechanisms underpinning these differences are still not fully understood^19,39^. Similar cues govern host specificity, although many species of mosquitoes will feed on a diversity of hosts across higher taxa. Other mechanisms by which elevated CO_2_ might promote speciation in mosquitoes are unclear, but one possibility is that females may have evolved a variety of other more specific mechanisms for locating their hosts at greater distances, and these may have functioned as potential isolating mechanisms.

The effects of elevated atmospheric CO_2_ upon mosquito development are not well understood. In general terms, elevated CO_2_ levels are known to have detrimental effects on the quality of nutrients available to many species that feed upon leaf litter as larvae^21^, thereby reducing rates of larval development. By contrast, raised CO_2_ may have indirect and beneficial effects, mediated by its influence upon ambient global temperatures. These include reduced hatching times and increased survival rates^40,41^, both of which increase the rate of transfer of mosquito-borne diseases^42,43^. Elevated CO_2_ may also have direct effects on the dynamics of interspecific and intraspecific competition between aquatic larvae^43^, changes that may also have promoted speciation. Higher atmospheric CO_2_ may also have macro-evolutionary effects. In land plants, raised CO_2_, humidity, and global temperatures correlate with increased rates of diversification^44^. Many groups of insects, including mosquitoes, have close associations with plants, and their patterns of diversification are broadly correlated^45,46^. Elevated CO_2_ has also been shown to increase the diversification rate of large mammalian herbivores, mediated via its effects upon vegetation. Large mammals are, in turn, predominant hosts of numerous mosquito species^47^. Therefore, there are a number of plausible mechanisms that could link global warming and elevated atmospheric CO_2_ to increased rates of speciation in mosquitoes, and that could also contribute to the missing processes identified in our analyses.

Given the historical association between climate change and the rise of mosquitoes, the projected anthropogenic rises in atmospheric CO_2_ pressures and concomitant increases in global mean temperatures appear likely to elevate rates of speciation across Culicidae. This may, in turn, increase the probability of new vector/pathogen interactions arising, particularly given the plasticity of these relationships across the phylogeny. This effect is in addition to the troubling increases in vector distributions projected for most climate change scenarios, such that vectors and diseases are likely to re-establish themselves in regions from which they have been eradicated^8,48^.

## Methods

### Building the supertree

Potential source papers were identified using the Web of Science. We used the following search terms: phylog*, taxonom*, systematic*, and clad*, coincident with any scientific and common names constituent within the Culicidae, from families to genera. Each source paper was inspected manually for one or more phylogenetic trees, and the references cited by each paper were similarly trawled for additional trees. In this manner, we collated 550 source trees from 284 source papers published up to the end of July 2014 (Supplementary References: Part 1). Each tree was digitised precisely in its published form using *Mesquite* 3.11 ^49^. The Supertree Toolkit 2 (STK)^50^ was used to standardise these sources and to produce a single matrix representation of their structure using group inclusion characters^51^. We excluded synonyms and standardised species names using the mosquito taxonomic inventory website (http://mosquito-taxonomic-inventory.info/). The protocols described by Davis and Page^18^ were followed to ensure the independence of each source. Outgroup taxa were removed from the entire data set, and replaced by an all zero outgroup. The resulting matrix representation was analysed using flat-weighted maximum parsimony in TNT 1.5 ^52^. The analysis followed Davis et al.^10^. We ran multiple replications at level 10, with 1000 random additions of taxa and TBR branch swapping. One thousand most parsimonious trees, each of 5321 steps, were saved and summarised as a Maximum Agreement Subtree in PAUP*4.0a151 ^53^. The resulting supertree comprised 1000 taxa (a coincidentally round number) within 103 genera (according to the classification of Reinert et al.^19^).

### Time calibration

Only nine extant genera are known as fossils, and we used the earliest known record of each of these as calibration points in our supertree. A patchy fossil record meant that many branches of our tree had no reliable fossil calibration dates, and we therefore used molecular estimates of divergence times for 18 additional genera^39^, as well as for the entire family and the subfamily Culicinae. This method of supplementing fossil divergence dates with molecular dates was successfully used in a recent supertree of caridean shrimps^54^. Genera classified within *Aedes* in the classification of system of Wilkerson^55^ were treated as *Aedes* during the time calibration. The node calibration dates are summarised in Supplementary Data 1. The R packages *Paleotree*^56^ and *Strap*^57^ were used to scale and plot the supertree.

### Data on disease vectors

Data on the diseases vectored by each species of mosquito were obtained from Norbert et al.^6^. We also searched Web of Science for the names of all pathogens and the names of the diseases that they cause, coincident with any of the stems: vect*, transmit*, mosquit*, culicid*, pathog* or vir*. All of the sources consulted are listed in Supplementary References (Part 2), while the resulting list of vectors and diseases is available as Supplementary Data 2.

### Speciation rate and correlations

Speciation rate parameters were modelled on the time-calibrated tree using BAMM^12^. We sampled four MCMC chains of 10 million generations every 10,000 iterations, with a burn-in of 10%, for each phylogeny. Full analytical parameters and settings are given in Supplementary Note 1, while the sampling file is provided as Supplementary Note 2. The R package *BAMMtools*^58^ was then used to identify significant speciation rate shifts across the entire tree (all Culicidae), and for the subclades Anophelinae and Culicinae. We also modelled vector and non-vector species separately. Speciation rates through time were modelled using both *λ* and *μ* parameters and then tested in both *λ* and *μ* calculations. Global temperature data and R code were derived from Davis et al.^10^. Atmospheric CO_2_ concentration data were acquired from Bergman et al.^59^ (Supplementary Data 3). For the DCCA analysis, we used a window for temperature and CO_2_ concentration data from 200 mya to the present. DCCA correlations of all realisations of the speciation curve in the MCMC analysis (9000 data pairs in total) were performed for all Culicidae and the subclades against the temperature and atmospheric CO_2_ concentration curves. Specifically, we tested whether the distribution of all 9000 correlation coefficients differed from the null hypothesis of zero, which implies no correlation. The correlations between clade (subfamily and vector/non-vector) and tip speciation rates were tested using the pgls function implemented in R package *caper*^60^.

### Information transfer

Information Theory has previously been used to explore causality in palaeontological and geological data sets with great success. Transfer entropy (TE) is a directional information flow method that quantifies the coherence between continuous variables in time^61^. It is an extension of the mutual information method, but can take into account the direction of information transfer by assuming that the processes can be described by a Markov model. Transfer Entropy reduces to a linear Granger causality process (whereby a signal in one time series gives a linear response to the second time series) when the two time series can be linked via autoregressive processes. However, TE makes fewer assumptions regarding the linearity of the processes involved, and is therefore more suitable for analysing causality when the processes involved are unknown^62,63^. Transfer Entropy is calculated using:

$$T_{X \to Y} = {\sum} {p\left( {Y_{n + 1},Y\frac{{(k)}}{n},X\frac{{(l)}}{n}} \right){\mathrm{log}}\left( {\frac{{p\left( {Y_{n + 1} \vee Y\frac{{(k)}}{n},X\frac{{(l)}}{n}} \right)}}{{p\left( {Y_{n + 1} \vee Y\frac{{(k)}}{n}} \right.}}} \right)},$$

where *T*_*X*→*Y*_ is the TE from time series *X* to time series *Y*, both of which have data at time *n*, and *k* and *l* are the embedding dimensions of the two time series respectively. As in Davis et al.^64^, we used the R (R Core Team 2017) package *TransferEntropy*^65^ which implements the above equation using a nearest neighbour algorithm^66^. This function returns a numeric value where 0 indicates no information transfer, positive numbers indicate information transfer, and negative numbers indicate misinformation transfer. The latter implies that there are other, unspecified processes interacting^29^. The embedding dimensions of the time series were estimated using the R package *nonlinearTseries*^67^.

We calculated TE for four time series: speciation rate in Culicidae, speciation rate in Mammalia, global palaeo-temperature and atmospheric CO_2_. We derived TE values from each climatic variable to each taxon and also between mosquitoes and mammals, in both directions of putative transfer. The mammalian speciation rate time series was inferred from a BAMM analysis of a recent, almost complete mammal phylogeny^68^. The significance of TE values was determined by randomising the source time series 250 times to create surrogates, and leaving the target time series unchanged. If the TE value was outside the 95% interval of the surrogate TE values, it was deemed significant^69^.

### Speciation rates verification

Molecular trees of Culicidae and Anophelinae were built using data from previous studies. The data set underpinning the phylogeny of Culicidae was obtained from Reidenbach et al.^39^, and comprised six nuclear gene protein-coding genes (*arginine kinase*, CAD, *catalase*, *enolase*, *hunchback* and *white*) coded for 25 ingroup genera and 2 outgroup taxa. The data set underpinning the tree of Anophelinae comprised three mitochondrial genes (*COI*, *ND2*, *ND3*) sequenced for 19 ingroup and 2 outgroup species. The subgenera *Cellia*, *Nyssorhynchus*, *Anopheles*, *Lophopodomyia*, *Kerteszia* and *Stethomyia* each had 2−4 representatives. Genbank accession numbers of the sequences used in the two data sets are presented in Supplementary Tables 4 and 5. The sequences were aligned in MEGA 7 ^70^. Tree topologies were obtained by ML analyses. Heuristic searches were performed in PAUP*4.0a151 using a GTR + G + I model. We also produced neighbour-joining (NJ) trees and estimated clade support using 1000 bootstrap resampling replicates. Estimations of divergence times and diversification rates were calculated using a fossilised birth−death model implemented in BEAST 2 ^26^. The following five fossil nodes were used for time-calibration: *Culex pipiens* (55.8 Mya), *Culiseta gedanica* (55.8 Mya), *Ochlerotatus serafini* (55.8 Mya), *Toxorhynchites mexicanus* (28.5 Mya), *Anopheles dominicanus* (40.4 Mya). The following parameters were used during the two analyses: GTR substitution site model, relaxed clock exponential model, running two MCMC chains of 200,000,000 generations with 100,000 sampled and 25% burned-in.

## Electronic supplementary material

Supplementary Information

Description of Supplementary Data

Supplementary Data 1

Supplementary Data 2

Supplementary Data 3

## Data Availability

All data generated or analysed during this study are included in this published article (and its Supplementary Information files).
