# Supplementary material for: Elevated atmospheric CO2 promoted speciation in mosquitoes (Diptera, Culicidae)
Source: Commun Biol. 2018 Nov 5;1:182. doi: 10.1038/s42003-018-0191-7 (PMC6218564; doi:10.1038/s42003-018-0191-7)
Supplement: Supplementary file 1 — Supplementary Information [file 42003_2018_191_MOESM1_ESM.pdf]

Supplementary Figure 1.

Supertree of Culicidae, with tribes indicated. Branches of Culicinae are shaded in grey, while branches of Anophelinae (approximately the top right quadrant) constitute the remainder.

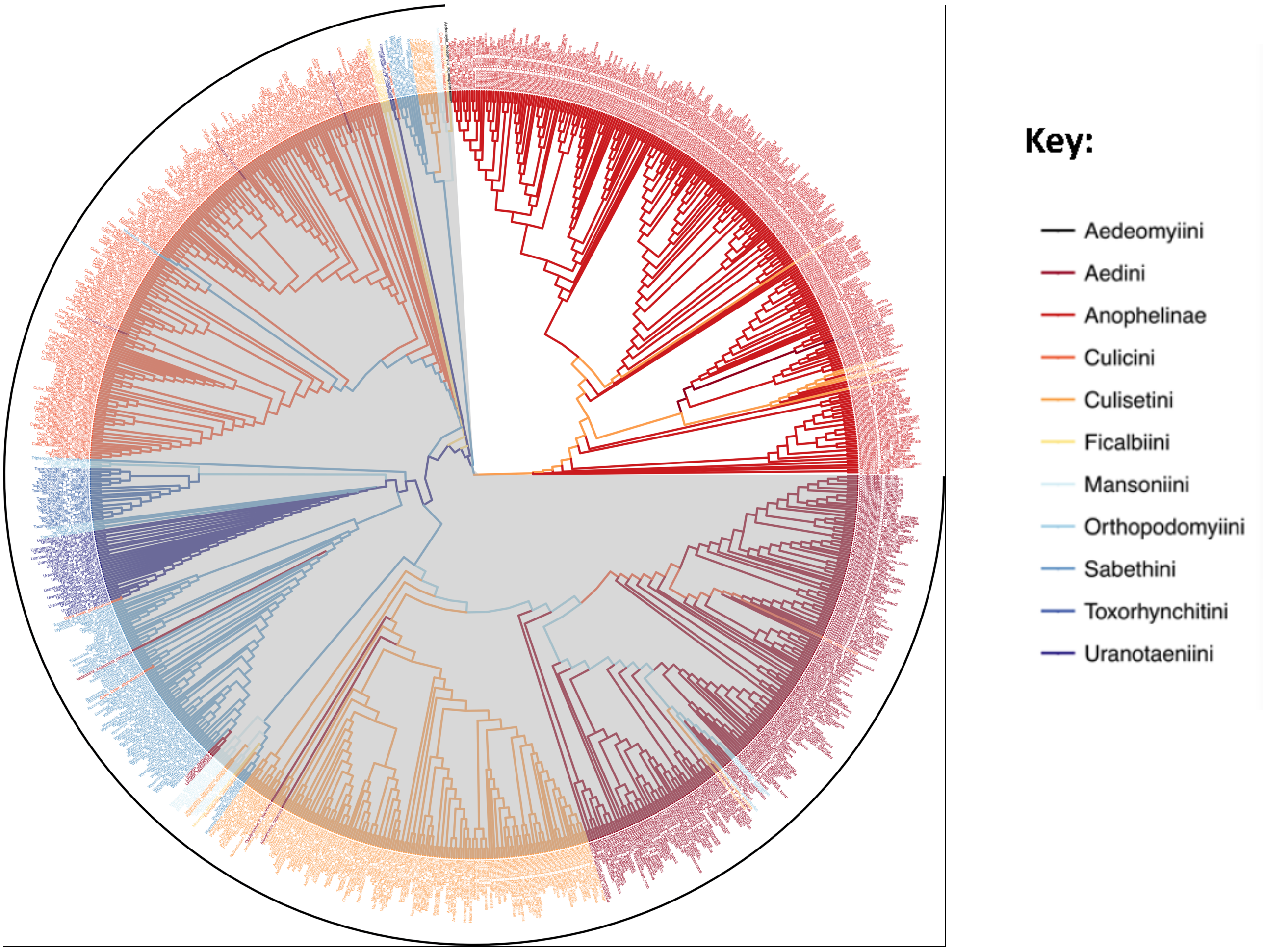

Supplementary Table 1.
The timing of speciation rate shifts for all Culicidae, vector and non-vector species (mya).

Culicidae	Vector	Non-Vector
**194.83**	194.83	158.33
**194.55**	194.62	130.76
**194.39**	194.55	108.54
**184.07**	186.74	100.73
**182.02**	184.07	69.09
**174.76**	174.76	29.71
**158.33**	154.64	26.66
**154.64**	153.79	26.45
**153.79**	151.30	25.81
**108.54**	27.32	24.81
**29.71**	26.45	21.80
**26.66**	20.75	20.75
**26.45**		
**25.81**		
**25.40**		
**24.81**		
**21.80**		
**20.75**		

Supplementary Table 2.

Mean speciation and extinction rate through time of groups of Culicidae.

CATEGORY	Average rate through time (lineage/myr)	
	λ (speciation rate)	μ (extinction rate)
**Culicidae**	0.0314	0.0085
**Vector**	0.0355	0.0061
**Non-Vector**	0.0316	0.0090
**Culicinae**	0.0283	0.0087
**Anophelinae**	0.0772	0.0063

Supplementary Table 3.

Transfer Entropy signals and the results of the significance tests.

CATEGORY	Transfer entropy	Confidence Interval 5%	Confidence Interval 50%	Confidence Interval 95%
PCO_2 -> mosquito speciation	-1.949549	-0.225023051	-0.003545748	0.261454000
PCO_2 -> mammal speciation	-1.861004	-0.246155865	0.004749652	0.267307701
Temperature -> mammal speciation	-0.7043328	-0.067518599	0.006100759	0.081754266
Temperature -> mosquito speciation	-0.8035503	-0.064090864	0.001473601	0.066812981
Mammal speciation -> mosquito speciation	0.2217302	-0.144325187	0.006450838	0.183470494

Supplementary Table 4. Genbank accession numbers of the sequences used in building the phylogenetic tree of Culicidae.

Names	CAD	white	hunchback	catalase	arginine_kinase	enolase
Aedeomyia_squamipennis	GQ906830.1	AF318199.1	GQ906902.1	GQ906855.1	GQ906806.1	GQ906879.1
Aedes_aegypti	NC_035107.1/gene=LOC5572005	NC_035107.1/gene=LOC23687419	NC_035109.1/gene=LOC5567202	NC_035108.1/gene=LOC5577893	NC_035108.1/gene=LOC5571596	NC_035108.1/gene=LOC5571664
Anopheles_atroparvus	GQ906831.1	GQ906927.1	GQ906903.1	GQ906856.1	GQ906831.1	GQ906903.1
Armigeres_subalbatus	GQ906832.1	AF318200.1	GQ906904.1	EU210286.1	MG232402.1	MG232516.1
Bironella_gracilis	GQ906833.1		GQ906905.1	GQ906857.1	GQ906808.1	GQ906881.1
Chaoborus_astictopus	GQ906853.1	U73838.1	GQ906925.1	GQ906877.1	GQ906828.1	GQ906900.1
Coquillettidia_perturbans	GQ906835.1		GQ906907.1	GQ906859.1	GQ906810.1	GQ906883.1
Culex_quinquefasciatus	NW_001886843.1	NW_001886814.1	NW_001887131.1	XM_001848521.1	NW_001886878.1	NW_001886711.1
Culiseta_inornata	GQ906834.1	GQ906928.1	GQ906906.1	GQ906858.1	GQ906809.1	GQ906882.1
Eretmapodites_quinquevittatus	GQ906836.1	GQ906929.1	GQ906908.1	GQ906860.1	GQ906811.1	GQ906884.1
Eucorethra_underwoodi	GQ906854.1	U73833.1	GQ906926.1	GQ906878.1	GQ906829.1	GQ906901.1
Haemagogus_equinus	GQ906861.1	U73834.1	GQ906909.1	GQ906861.1	GQ906812.1	GQ906885.1
Limatus_durhami	GQ906838.1	GQ906930.1	GQ906910.1	GQ906862.1	GQ906813.1	GQ906886.1
Malaya_genurostris	GQ906839.1	GQ906931.1	GQ906911.1	GQ906863.1	GQ906814.1	GQ906887.1
Maorigoeldia_argyropus	GQ906840.1	GQ906932.1	GQ906912.1	GQ906864.1	GQ906815.1	GQ906888.1
Mimomyia_luzonensis	GQ906841.1	GQ906933.1	GQ906913.1	GQ906865.1	GQ906816.1	GQ906889.1
Ochlerotatus_triseriatus	GQ906842.1	U73827.1	GQ906914.1	GQ906866.1	GQ906842.1	GQ906890.1
Opifex_fuscus	GQ906843.1	GQ906934.1	GQ906915.1	GQ906867.1	GQ906818.1	GQ906891.1
Orthopodomyia alba	GQ906844.1	AF318206.1	GQ906916.1	GQ906868.1	GQ906819.1	GQ906892.1
Psorophora_ferox	GQ906845.1	GQ906935.1	GQ906917.1	GQ906869.1	GQ906820.1	GQ906893.1
Sabethes_cyaneus	GQ906846.1	U73835.1	GQ906918.1	GQ906870.1	GQ906821.1	
Shannoniana_fluviatilis	GQ906847.1	GQ906936.1	GQ906919.1	GQ906871.1	GQ906822.1	GQ906894.1
Trichoprosopon_digitatum	GQ906849.1	GQ906937.1	GQ906921.1	GQ906873.1	GQ906873.1	GQ906896.1
Tripteroides_bambusa	GQ906850.1	U73837.1	GQ906922.1	GQ906874.1	GQ906825.1	GQ906897.1
Uranotaenia_sapphirina	GQ906851.1	AF318209.1	GQ906923.1	GQ906875.1	GQ906826.1	GQ906898.1
Wyeomyia_smithii	GQ906852.1	AY055811.1	GQ906924.1	GQ906876.1	GQ906827.1	GQ906899.1

Supplementary Table 5. Genbank accession numbers of the sequences used in building the phylogenetic tree of Anophelinae.

Name	Accession number of the complete mitochondrion genome
Anopheles_Anopheles_eiseni	MF381706
Anopheles_Anopheles_forattinii	MF381664
Anopheles_Anopheles_minor	MF381684
Anopheles_Anopheles_quadrimaculatus	L04272
Anopheles_Cellia_farauti	JX219735
Anopheles_Cellia_gambiae	MG930872
Anopheles_Cellia_punctulatus	JX219738
Anopheles_Kerteszia_cruzii	KU551284
Anopheles_Kerteszia_homunculus	KU551283
Anopheles_Kerteszia_laneanus	KU551288
Anopheles_Lophopodomyia_gilesi	MF381630
Anopheles_Lophopodomyia_pseudotibiamaculatus	MF381737
Anopheles_Nyssorhynchus_albitarsis	HQ335346
Anopheles_Nyssorhynchus_marajoara	MF381646
Anopheles_Nyssorhynchus_triannulatus	MF381730
Anopheles_Stethomyia_kompi	MF381721
Anopheles_Stethomyia_nimbus	MF381659
Dixella_aestivalis	KT878382
Bironella_hollandi	MF381612
Aedes_aegypti	EU352212

Supplementary Note 1.

The settings file used for our BAMM analyses.

```
# BAMM configuration file for speciation/extinction analysis
# ==========================================================
#
# Format
# ------
#
#     - Each option is specified as: option_name = option_value
#     - Comments start with # and go to the end of the line
#     - True is specified with "1" and False with "0"


################################################################################
####
# GENERAL SETUP AND DATA INPUT
################################################################################
####

modeltype = speciationextinction
# Specify "speciationextinction" or "trait" analysis

treefile = tctreemosquito.tre
# File name of the phylogenetic tree to be analyzed

runInfoFilename = run_info.txt
# File name to output general information about this run

sampleFromPriorOnly = 0
# Whether to perform analysis sampling from prior only (no likelihoods computed)

runMCMC = 1
# Whether to perform the MCMC simulation. If runMCMC = 0, the program will only
# check whether the data file can be read and the initial likelihood computed

simulatePriorShifts = 1
# Whether to simulate the prior distribution of the number of shift events,
# given the hyperprior on the Poisson rate parameter. This is necessary to
# compute Bayes factors

loadEventData = 0
# Whether to load a previous event data file

eventDataInfile = event_data_in.txt
# File name of the event data file to load, used only if loadEventData = 1

initializeModel = 1
# Whether to initialize (but not run) the MCMC. If initializeModel = 0, the
# program will only ensure that the data files (e.g., treefile) can be read

useGlobalSamplingProbability = 0
# Whether to use a "global" sampling probability. If False (0), expects a file
# name for species-specific sampling probabilities (see sampleProbsFilename)
```

```
globalSamplingFraction = 0
# The sampling probability. If useGlobalSamplingFraction = 0, this is ignored
# and BAMM looks for a file name with species-specific sampling fractions

sampleProbsFilename = mossampling.txt
# File name containing species-specific sampling fractions

# seed = -1
# Seed for the random number generator.
# If not specified (or is -1), a seed is obtained from the system clock

overwrite = 1
# If True (1), the program will overwrite any output files in the current
# directory (if present)


############################################################################
####
# PRIORS
############################################################################
####

poissonRatePrior = 1.0
# The rate parameter of the exponential prior on the rate parameter of the
# Poisson process. Smaller values favor greater numbers of distinct
# evolutionary regimes on the tree
# Suggested values:
#    poissonRatePrior = 1.0 for small trees (< 500 tips)
#     poissonRatePrior = 0.1 or even 0.02 for large trees (> 5000 tips)

lambdaInitPrior = 1.0
# Prior (rate parameter of exponential) on the initial lambda value for rate
# regimes

lambdaShiftPrior = 0.05
# Prior (std dev of normal) on lambda shift parameter for rate regimes
# You cannot adjust the mean of this distribution (fixed at zero, which is
# equal to a constant rate diversification process)

muInitPrior = 1.0
# Prior (rate parameter of exponential) on extinction rates

lambdaIsTimeVariablePrior = 1
# Prior (probability) of the time mode being time-variable (vs. time-constant)


############################################################################
####
# MCMC SIMULATION SETTINGS & OUTPUT OPTIONS
############################################################################
####

numberOfGenerations = 20000000
# Number of generations to perform MCMC simulation

mcmcOutfile = mcmc_out.txt
# File name for the MCMC output, which only includes summary information about
# MCMC simulation (e.g., log-likelihoods, log-prior, number of processes)
```

```
mcmcWriteFreq = 2000
# Frequency in which to write the MCMC output to a file

eventDataOutfile = event_data.txt
# The raw event data (these are the main results). ALL of the results are
# contained in this file, and all branch-specific speciation rates, shift
# positions, marginal distributions etc can be reconstructed from this output.
# See R package BAMMtools for working with this output

eventDataWriteFreq = 2000
# Frequency in which to write the event data to a file

printFreq = 1000
# Frequency in which to print MCMC status to the screen

acceptanceResetFreq = 2000
# Frequency in which to reset the acceptance rate calculation
# The acceptance rate is output to both the MCMC data file and the screen

# outName = BAMM
# Optional name that will be prefixed on all output files (separated with "_")
# If commented out, no prefix will be used


#############################################################################
####
# OPERATORS: MCMC SCALING OPERATORS
#############################################################################
####

updateLambdaInitScale = 2.0
# Scale parameter for updating the initial speciation rate for each process

updateLambdaShiftScale = 0.1
# Scale parameter for the exponential change parameter for speciation

updateMuInitScale = 2.0
# Scale parameter for updating initial extinction rate for each process

updateEventLocationScale = 0.05
# Scale parameter for updating LOCAL moves of events on the tree
# This defines the width of the sliding window proposal

updateEventRateScale = 4.0
# Scale parameter (proportional shrinking/expanding) for updating
# the rate parameter of the Poisson process


#############################################################################
####
# OPERATORS: MCMC MOVE FREQUENCIES
#############################################################################
####

updateRateEventNumber = 0.1
# Relative frequency of MCMC moves that change the number of events
```

```
updateRateEventPosition = 1
# Relative frequency of MCMC moves that change the location of an event on the
# tree

updateRateEventRate = 1
# Relative frequency of MCMC moves that change the rate at which events occur

updateRateLambda0 = 1
# Relative frequency of MCMC moves that change the initial speciation rate
# associated with an event

updateRateLambdaShift = 1
# Relative frequency of MCMC moves that change the exponential shift parameter
# of the speciation rate associated with an event

updateRateMu0 = 1
# Relative frequency of MCMC moves that change the extinction rate for a given
# event

updateRateLambdaTimeMode = 0
# Relative frequency of MCMC moves that flip the time mode
# (time-constant <=> time-variable)

localGlobalMoveRatio = 10.0
# Ratio of local to global moves of events


############################################################################
####
# INITIAL PARAMETER VALUES
############################################################################
####

lambdaInit0 = 0.032
# Initial speciation rate (at the root of the tree)

lambdaShift0 = 0
# Initial shift parameter for the root process

muInit0 = 0.005
# Initial value of extinction (at the root)

initialNumberEvents = 0
# Initial number of non-root processes


############################################################################
####
# METROPOLIS COUPLED MCMC
############################################################################
####

numberOfChains = 4
# Number of Markov chains to run

deltaT = 0.01
# Temperature increment parameter. This value should be > 0
# The temperature for the i-th chain is computed as 1 / [1 + deltaT * (i - 1)]
```

```
swapPeriod = 1000
# Number of generations in which to propose a chain swap

chainSwapFileName = chain_swap.txt
# File name in which to output data about each chain swap proposal.
# The format of each line is [generation],[rank_1],[rank_2],[swap_accepted]
# where [generation] is the generation in which the swap proposal was made,
# [rank_1] and [rank_2] are the chains that were chosen, and [swap_accepted] is
# whether the swap was made. The cold chain has a rank of 1.


############################################################################
####
# NUMERICAL AND OTHER PARAMETERS
############################################################################
####

minCladeSizeForShift = 5
# Allows you to constrain location of possible rate-change events to occur
# only on branches with at least this many descendant tips. A value of 1
# allows shifts to occur on all branches.

segLength = 0.02
# Controls the "grain" of the likelihood calculations. Approximates the
# continuous-time change in diversification rates by breaking each branch into
# a constant-rate diversification segments, with each segment given a length
# determined by segLength. segLength is in units of the root-to-tip distance of
# the tree. So, if the segLength parameter is 0.01, and the crown age of your
# tree is 50, the "step size" of the constant rate approximation will be 0.5.
# If the value is greater than the branch length (e.g., you have a branch of
# length < 0.5 in the preceding example) BAMM will not break the branch into
# segments but use the mean rate across the entire branch.

Command line: bamm -c diversification.txt
Git commit id: 462bc39af9d4c4b4dd81a481f32c953e93a076e7
Random seed: 1497634665
Start time: Fri Jun 16 18:37:45 2017

Current parameter settings:
acceptanceInfoFileName    acceptance_info.txt
acceptanceResetFreq    2000
alwaysRecomputeE0    0
autotune    0
branchRatesWriteFreq    0
chainSwapFileName    chain_swap.txt
checkUltrametric    1
combineExtinctionAtNodes    if_different
conditionOnSurvival    -1
deltaT    0.01
eventDataInfile    event_data_in.txt
eventDataOutfile    event_data.txt
eventDataWriteFreq    2000
expectedNumberOfShifts    0.0
extinctionProbMax    0.9999
fastSimulatePriorExperimental    0
fastSimulatePrior_BurnIn    0.05
fastSimulatePrior_Generations    5000000
```

```
fastSimulatePrior_SampleFreq   50
globalSamplingFraction   0
initialNumberEvents   0
initializeModel   1
lambdaInit0   0.032
lambdaInitPrior   1.0
lambdaInitRootPrior   -1.0
lambdaIsTimeVariablePrior   1
lambdaOutfile   lambda_rates.txt
lambdaShift0   0
lambdaShiftPrior   0.05
lambdaShiftRootPrior   -1.0
loadEventData   0
localGlobalMoveRatio   10.0
maxNumberEvents   5000
mcmcOutfile   mcmc_out.txt
mcmcWriteFreq   2000
minCladeSizeForShift   5
modeltype   speciationextinction
muInit0   0.005
muInitPrior   1.0
muInitRootPrior   -1.0
muOutfile   mu_rates.txt
muShift0   0.0
muShiftPrior   1.0
muShiftRootPrior   -1.0
numberOccurrences   0
numberOfChains   4
numberOfGenerations   20000000
observationTime   -1
outName
outputAcceptanceInfo   0
overwrite   1
poissonRatePrior   1.0
preservationRateInit   0
preservationRatePrior   1.0
printFreq   1000
priorOutputFileName   prior_probs.txt
priorSim_IntervalGenerations   5000
runInfoFilename   run_info.txt
runMCMC   1
sampleFromPriorOnly   0
sampleProbsFilename   mossampling.txt
seed   -1
segLength   0.02
simulatePriorShifts   1
swapPeriod   1000
treefile   tctreemosquito.tre
updateEventLocationScale   0.05
updateEventRateScale   4.0
updateLambdaInitScale   2.0
updateLambdaShiftScale   0.1
updateMuInitScale   2.0
updateMuShiftScale   0.0
updatePreservationRateScale   1.0
updateRateEventNumber   0.1
updateRateEventNumberForBranch   0.0
updateRateEventPosition   1
```

```
updateRateEventRate    1
updateRateLambda0    1
updateRateLambdaShift    1
updateRateLambdaTimeMode    0
updateRateMu0    1
updateRateMuShift    0.0
updateRatePreservationRate    -1
useGlobalSamplingProbability    0
validateEventConfiguration    0
writeMeanBranchLengthTrees    0
End time: Sat Jun 17 05:55:06 2017
```

Supplementary Note 2.

The sampling file used for our BAMM analyses.

```
1.0
Culex_Oculeomyia_annulioris	Culicinae	0.245516792
Culex_Melanoconion_pilosus	Culicinae	0.245516792
Culex_Melanoconion_erraticus	Culicinae	0.245516792
Culex_Culex_duttoni	Culicinae	0.245516792
Culex_Culex_alani	Culicinae	0.245516792
Culex_Culex_plicatus	Culicinae	0.245516792
Culex_Culex_pipiens	Culicinae	0.245516792
Culex_Culex_australicus	Culicinae	0.245516792
Culex_Culex_hutchinsoni	Culicinae	0.245516792
Culex_C_p_quinquefasciatus	Culicinae	0.245516792
Culex_Culex_pipiens_pallens	Culicinae	0.245516792
Culex_Culex_pipiens_molestus	Culicinae	0.245516792
Culex_Culex_pipiens_pipiens	Culicinae	0.245516792
Culex_Culex_fuscocephala	Culicinae	0.245516792
Culex_Culex_curvibrachius	Culicinae	0.245516792
Culex_Culex_trifilatus	Culicinae	0.245516792
Culex_Culex_torrentium	Culicinae	0.245516792
Culex_Culiciomyia_nigropunctatu	Culicinae	0.245516792
Culex_Culiciomyia_pallidothorax	Culicinae	0.245516792
Culex_Culex_vagans	Culicinae	0.245516792
Culex_Culiciomyia_ryukyensis	Culicinae	0.245516792
Culex_Culex_thriambus	Culicinae	0.245516792
Culex_Culex_whitmorei	Culicinae	0.245516792
Culex_Oculeomyia_poicilipes	Culicinae	0.245516792
Culex_Oculeomyia_infula	Culicinae	0.245516792
Phagomyia_khazani	Culicinae	0.245516792
Culex_Culiciomyia_nebulosus	Culicinae	0.245516792
Culex_O_bitaeniorhynchus	Culicinae	0.245516792
Culex_Culex_afridii	Culicinae	0.245516792
Culex_Culex_annulirostris	Culicinae	0.245516792
Culex_Culex_mimeticus	Culicinae	0.245516792
Culex_Culex_mimulus	Culicinae	0.245516792
Culex_Culex_tritaeniorhynchus	Culicinae	0.245516792
Culex_Culex_palpalis	Culicinae	0.245516792
Culex_Culex_annulus	Culicinae	0.245516792
Culex_Culex_vishnui	Culicinae	0.245516792
Culex_Culex_pseudovishnui	Culicinae	0.245516792
Culex_Culex_sitiens	Culicinae	0.245516792
Culex_Culex_gelidus	Culicinae	0.245516792
Culex_Culex_cuyanus	Culicinae	0.245516792
Culex_Culex_lygrus	Culicinae	0.245516792
Culex_Culex_interrogator	Culicinae	0.245516792
Culex_Culex_restuans	Culicinae	0.245516792
Culex_Culex_tarsalis	Culicinae	0.245516792
Culex_Culex_habilitator	Culicinae	0.245516792
Culex_Culex_bonneae	Culicinae	0.245516792
Culex_Culex_saltanensis	Culicinae	0.245516792
Culex_Culex_maracayensis	Culicinae	0.245516792
Culex_Culex_covagarciai	Culicinae	0.245516792
Culex_Culex_maxi	Culicinae	0.245516792
Culex_Culex_brevispinosus	Culicinae	0.245516792
Culex_Culex_paramaxi	Culicinae	0.245516792
Culex_Culex_coronator	Culicinae	0.245516792
```

Culex_Culex_usquatus	Culicinae	0.245516792
Culex_Culex_camposi	Culicinae	0.245516792
Culex_Culex_usquatissimus	Culicinae	0.245516792
Culex_Culex_surinamensis	Culicinae	0.245516792
Culex_Culex_interfor	Culicinae	0.245516792
Culex_Culex_brethesi	Culicinae	0.245516792
Culex_Culex_spinosus	Culicinae	0.245516792
Culex_Culex_ameliae	Culicinae	0.245516792
Culex_Culex_eduardoi	Culicinae	0.245516792
CulexSP1	Culicinae	0.245516792
CulexSP2	Culicinae	0.245516792
Culex_Culex_abnormalis	Culicinae	0.245516792
Culex_Culex_nigripalpus	Culicinae	0.245516792
Culex_Culex_foliaceus	Culicinae	0.245516792
Culex_Culex_inflictus	Culicinae	0.245516792
Culex_Culex_chitae	Culicinae	0.245516792
Culex_Culex_chidesteri	Culicinae	0.245516792
Culex_Culex_guayasi	Culicinae	0.245516792
Culex_Culex_diplophyllum	Culicinae	0.245516792
Culex_Culex_lahillei	Culicinae	0.245516792
Culex_Culex_carcinoxenus	Culicinae	0.245516792
Culex_Culex_bidens	Culicinae	0.245516792
Culex_Culex_quitensis	Culicinae	0.245516792
Culex_Culex_levicastilloi	Culicinae	0.245516792
Culex_Culex_archegus	Culicinae	0.245516792
Culex_Culex_declarator	Culicinae	0.245516792
Culex_Culex_bitaeniorhynchus	Culicinae	0.245516792
Culex_Culex_mollis	Culicinae	0.245516792
Culex_Culex_tatoi	Culicinae	0.245516792
Culex_Culex_fernandezi	Culicinae	0.245516792
Culex_Culex_dolosus	Culicinae	0.245516792
Culex_Culex_erythrothorax	Culicinae	0.245516792
Culex_Culex_salinarius	Culicinae	0.245516792
Culex_Culex_articularis	Culicinae	0.245516792
Culex_Culex_acharistus	Culicinae	0.245516792
Culex_Culex_janitor	Culicinae	0.245516792
Culex_Phenacomyia_corniger	Culicinae	0.245516792
Culex_Phenacomyia_lactator	Culicinae	0.245516792
Culex_Phytotelmatomyia_castroi	Culicinae	0.245516792
Culex_Phytotelmatomyia_hepperi	Culicinae	0.245516792
Culex_Sirivanakarnius_boninensi	Culicinae	0.245516792
Culex_Neoculex_territans	Culicinae	0.245516792
Culex_Nicaromyia_nicaroensis	Culicinae	0.245516792
Culex_Neoculex_martinii	Culicinae	0.245516792
Culex_Tinolestes_latisquama	Culicinae	0.245516792
Galindomyia_leei	Culicinae	0.245516792
Deinocerites_cancer	Culicinae	0.245516792
Deinocerites_mathesoni	Culicinae	0.245516792
Deinocerites_magnus	Culicinae	0.245516792
Culex_Melanoconion_taeniopus	Culicinae	0.245516792
Culex_Belkinomyia_eldridgei	Culicinae	0.245516792
Culex_Aedinus_amazonensis	Culicinae	0.245516792
Culex_Aedinus_accelerans	Culicinae	0.245516792
Culex_Anoediporpa_conservator	Culicinae	0.245516792
Culex_Anoediporpa_originator	Culicinae	0.245516792
Culex_Melanoconion_spissipes	Culicinae	0.245516792
Culex_Melanoconion_atratus	Culicinae	0.245516792
Culex_Melanoconion_sacchettae	Culicinae	0.245516792

Culex_Melanoconion_gnomatos	Culicinae	0.245516792
Culex_Melanoconion_portesi	Culicinae	0.245516792
Culex_Melanoconion_vomerifer	Culicinae	0.245516792
Culex_Melanoconion_pedroi	Culicinae	0.245516792
Culex_Melanoconion_ribeirensis	Culicinae	0.245516792
Culex_Melanoconion_adamesi	Culicinae	0.245516792
Culex_Microculex_imitator	Culicinae	0.245516792
Culex_Microculex_davisi	Culicinae	0.245516792
Culex_Micraedes_bisulcatus	Culicinae	0.245516792
Culex_Micraedes_antillummagnoru	Culicinae	0.245516792
Culex_Carrollia_infoliatus	Culicinae	0.245516792
Culex_Carrollia_iridescens	Culicinae	0.245516792
Culex_Neoculex_leonardi	Culicinae	0.245516792
Culex_Neoculex_apicalis	Culicinae	0.245516792
Culex_Lasiosiphon_adairi	Culicinae	0.245516792
Culex_Afroculex_lineatus	Culicinae	0.245516792
Culex_Maillotia_hortensis	Culicinae	0.245516792
Culex_Maillotia_arbieeni	Culicinae	0.245516792
Culex_Eumelanomyia_foliatus	Culicinae	0.245516792
Culex_E_inconspicuosus	Culicinae	0.245516792
Culex_L_rubithoracis	Culicinae	0.245516792
Culex_Lophoceraomyia_spiculosus	Culicinae	0.245516792
Culex_Lophoceraomyia_minor	Culicinae	0.245516792
Culex_Lophoceraomyia_uniformis	Culicinae	0.245516792
Culex_Lophoceraomyia_infantulus	Culicinae	0.245516792
Culex_Lophoceraomyia_cinctellus	Culicinae	0.245516792
Culex_Lophoceraomyia_peytoni	Culicinae	0.245516792
Culex_Lophoceraomyia_minutissim	Culicinae	0.245516792
Culex_Lophoceraomyia_variatus	Culicinae	0.245516792
Culex_Lophoceraomyia_rubithorac	Culicinae	0.245516792
Culex_Lophoceraomyia_tuberis	Culicinae	0.245516792
Culex_Lophoceraomyia_bicornutus	Culicinae	0.245516792
Culex_Eumelanomyia_malayi	Culicinae	0.245516792
Culex_Eumelanomyia_macrostylus	Culicinae	0.245516792
Culex_Eumelanomyia_richei	Culicinae	0.245516792
Culex_Eumelanomyia_okinawae	Culicinae	0.245516792
Culex_E_hayashii_ryukyuanus	Culicinae	0.245516792
Lorrainea_fumidus	Culicinae	0.245516792
Culex_Eumelanomyia_brevipalpis	Culicinae	0.245516792
Culex_Anoedioporpa_browni	Culicinae	0.245516792
Culex_Phytotelmatomyia_renatoi	Culicinae	0.245516792
Culex_Barraudius_modestus	Culicinae	0.245516792
Culex_Barraudius_pusillus	Culicinae	0.245516792
Culex_Acalleomyia_obscurus	Culicinae	0.245516792
Culex_Melanoconion_zeteki	Culicinae	0.245516792
Culex_Culiciomyia_kyotoensis	Culicinae	0.245516792
Culex_Culiciomyia_fragilis	Culicinae	0.245516792
Culex_Melanoconion_caudelli	Culicinae	0.245516792
Culex_Culex_apicinus	Culicinae	0.245516792
Culex_Allimanta_tramazayguesi	Culicinae	0.245516792
Culex_Melanoconion_ybarmis	Culicinae	0.245516792
Culex_Culex_scheuberi	Culicinae	0.245516792
Culex_Melanoconion_aliciae	Culicinae	0.245516792
Culex_Melanoconion_dyius	Culicinae	0.245516792
Culex_Carrollia_bonnei	Culicinae	0.245516792
Culex_Carrollia_urichii	Culicinae	0.245516792
Culex_Culex_atriceps	Culicinae	0.245516792
Culex_Culex_marquesensis	Culicinae	0.245516792

Culex_Kitzmilleria_moucheti	Culicinae	0.245516792
Culex_Acallyntrum_axillicola	Culicinae	0.245516792
Culex_Acallyntrum_perkinsi	Culicinae	0.245516792
Culex_Carrollia_rausseoi	Culicinae	0.245516792
Culex_Carrollia_bihaicolus	Culicinae	0.245516792
Culex_Culex_antennatus	Culicinae	0.245516792
Culex_Anoedioporpa_bamborum	Culicinae	0.245516792
Culex_Culex_laticinctus	Culicinae	0.245516792
Culex_Microculex_microphylus	Culicinae	0.245516792
Culex_Microculex_chryselatus	Culicinae	0.245516792
Culex_Microculex_pleuristriatus	Culicinae	0.245516792
Culex_Melanoconion_dunni	Culicinae	0.245516792
Culex_Melanoconion_albinensis	Culicinae	0.245516792
Culex_Melanoconion_nicceriensis	Culicinae	0.245516792
Culex_Culiciomyia_cinereus	Culicinae	0.245516792
Deinocerites_melanophylum	Culicinae	0.245516792
Culex_Culex_decens	Culicinae	0.245516792
Wyeomyia_Phoniomyia	Culicinae	0.245516792
Culex_Culex_theileri	Culicinae	0.245516792
Culex_Culex_univittatus	Culicinae	0.245516792
Culex_Culex_perexiguus	Culicinae	0.245516792
Culex_Culex_mirificus	Culicinae	0.245516792
Culex_Culex_simpsoni	Culicinae	0.245516792
Culex_Culex_sinaiticus	Culicinae	0.245516792
Howardina_bahamensis	Culicinae	0.245516792
Culex_Culex_thalassius	Culicinae	0.245516792
Culex_Culex_edwardsi	Culicinae	0.245516792
Culex_Culex_barraudi	Culicinae	0.245516792
Culex_Melanoconion_serratimarge	Culicinae	0.245516792
Lutzia_Metalutzia_fuscana	Culicinae	0.245516792
Lutzia_Metalutzia_halifaxii	Culicinae	0.245516792
Lutzia_Lutzia_allostigma	Culicinae	0.245516792
Lutzia_Metalutzia_tigripes	Culicinae	0.245516792
Lutzia_Lutzia_bigoti	Culicinae	0.245516792
Culex_Melanoconion_trifidus	Culicinae	0.245516792
Tripteroides_Trich_nepenthicola	Culicinae	0.245516792
Orthopodomyia_kummi	Culicinae	0.245516792
Orthopodomyia_signifera	Culicinae	0.245516792
Orthopodomyia_alba	Culicinae	0.245516792
Toxorhynchites_T_towadensis	Culicinae	0.245516792
Toxorhynchites_T_bickleyi	Culicinae	0.245516792
Toxorhynchites_T_manopi	Culicinae	0.245516792
Toxorhynchites_T_sunthorni	Culicinae	0.245516792
Toxorhynchites_T_inornatus	Culicinae	0.245516792
Toxorhynchites_T_splendens	Culicinae	0.245516792
Toxorhynchites_T_amboinensis	Culicinae	0.245516792
Toxorhynchites_T_quasiferox	Culicinae	0.245516792
Toxorhynchites_T_nepenthis	Culicinae	0.245516792
Toxorhynchites_T_funestus	Culicinae	0.245516792
Toxorhynchites_T_magnificus	Culicinae	0.245516792
Toxorhynchites_T_klossi	Culicinae	0.245516792
Toxorhynchites_T_metallicus	Culicinae	0.245516792
Toxorhynchites_T_leicesteri	Culicinae	0.245516792
Toxorhynchites_T_gravelyi	Culicinae	0.245516792
Toxorhynchites_T_m_yaeyamae	Culicinae	0.245516792
Toxorhynchites_T_gigantulus	Culicinae	0.245516792
Toxorhynchites_T_minimus	Culicinae	0.245516792
Toxorhynchites_T_acaudatus	Culicinae	0.245516792

Toxorhynchites_T_nigripes	Culicinae	0.245516792
Toxorhynchites_T_ater	Culicinae	0.245516792
Toxorhynchites_T_nepenthicola	Culicinae	0.245516792
Tripteroides_R_aranoides	Culicinae	0.245516792
Malaya_trichorostris	Culicinae	0.245516792
Uranotaenia_Uranotaenia_lowii	Culicinae	0.245516792
Uranotaenia_U_alboannulata	Culicinae	0.245516792
Uranotaenia_Uranotaenia_testace	Culicinae	0.245516792
Uranotaenia_U_annandalei	Culicinae	0.245516792
Uranotaenia_Uranotaenia_hebes	Culicinae	0.245516792
Uranotaenia_P_nivipleura	Culicinae	0.245516792
Uranotaenia_P_leiboensis	Culicinae	0.245516792
Uranotaenia_Pseudoficalbia_lui	Culicinae	0.245516792
Uranotaenia_P_lutescens	Culicinae	0.245516792
Uranotaenia_P_novobscura	Culicinae	0.245516792
Uranotaenia_P_obscura	Culicinae	0.245516792
Uranotaenia_P_bicolor	Culicinae	0.245516792
Uranotaenia_Pseudoficalbia_atra	Culicinae	0.245516792
Uranotaenia_P_unguiculata	Culicinae	0.245516792
Uranotaenia_P_maxima	Culicinae	0.245516792
Uranotaenia_Pseudoficalbia_koli	Culicinae	0.245516792
Uranotaenia_P_yaeyamana	Culicinae	0.245516792
Uranotaenia_P_jacksoni	Culicinae	0.245516792
Uranotaenia_P_abdita	Culicinae	0.245516792
Uranotaenia_P_enigmatica	Culicinae	0.245516792
Uranotaenia_P_spiculosa	Culicinae	0.245516792
Uranotaenia_P_recondita	Culicinae	0.245516792
Culex_Culex_guizhouensis	Culicinae	0.245516792
Uranotaenia_Uranotaenia_sociali	Culicinae	0.245516792
Uranotaenia_U_sapphirina	Culicinae	0.245516792
Uranotaenia_U_macfarlanei	Culicinae	0.245516792
Uranotaenia_P_jinhongensis	Culicinae	0.245516792
Uranotaenia_U_calosomata	Culicinae	0.245516792
Uranotaenia_Uranotaenia_somboon	Culicinae	0.245516792
Uranotaenia_U_lateralis	Culicinae	0.245516792
Uranotaenia_Uranotaenia_edwards	Culicinae	0.245516792
Topomyia_Suaymyiasp	Culicinae	0.245516792
Topomyia_Topomyia_lindsayi	Culicinae	0.245516792
Tripteroides_T_caeruleocephalus	Culicinae	0.245516792
Aedeomyia_Aedeomyia_catasticta	Culicinae	0.245516792
Maorigoeldia_argyropus	Culicinae	0.245516792
Runchomyia_Runchomyia_theobaldi	Culicinae	0.245516792
Wyeomyia_Decamyia_felicia	Culicinae	0.245516792
Wyeomyia_Decamyia_ulocoma	Culicinae	0.245516792
Wyeomyia_Wyeomyia_smithii	Culicinae	0.245516792
Wyeomyia_Wyeomyia_vanduzeei	Culicinae	0.245516792
Wyeomyia_Spilonympha_finlayi	Culicinae	0.245516792
Wyeomyia_Spilonympha_forcipenis	Culicinae	0.245516792
Wyeomyia_Spilonympha_bourrouli	Culicinae	0.245516792
Wyeomyia_Spilonympha_mystes	Culicinae	0.245516792
Wyeomyia_Spilonympha_aningae	Culicinae	0.245516792
Wyeomyia_Wyeomyia_grayii	Culicinae	0.245516792
Wyeomyia_albosquamata	Culicinae	0.245516792
Wyeomyia_argenteorostris	Culicinae	0.245516792
Wyeomyia_Hystatomyia_sp1	Culicinae	0.245516792
Wyeomyia_Dodecamyia_aphobema	Culicinae	0.245516792
Wyeomyia_Exallomyia_tarsata	Culicinae	0.245516792
Wyeomyia_Nunezia_bicornis	Culicinae	0.245516792

Wyeomyia_Phoniomyia_galvaoi	Culicinae	0.245516792
Wyeomyia_Dendromyia_sp1	Culicinae	0.245516792
Wyeomyia_Wyeomyia_medioalbipes	Culicinae	0.245516792
Wyeomyia_Cruzmyia_dyari	Culicinae	0.245516792
Limatus_durhamii	Culicinae	0.245516792
Limatus_asulleptus	Culicinae	0.245516792
Limatus_flavisetosus	Culicinae	0.245516792
Wyeomyia_Wyeomyia_codiocampa	Culicinae	0.245516792
Wyeomyia_Wyeomyia_oblita	Culicinae	0.245516792
Wyeomyia_Wyeomyia_lutzi	Culicinae	0.245516792
Wyeomyia_Zinzala_zinzala	Culicinae	0.245516792
Wyeomyia_Wyeomyia_arthrostigma	Culicinae	0.245516792
Wyeomyia_Triamyia_aporonoma	Culicinae	0.245516792
Wyeomyia_melanocephala	Culicinae	0.245516792
Wyeomyia_Wyeomyia_mitchellii	Culicinae	0.245516792
Wyeomyia_Menolepis_leucostigma	Culicinae	0.245516792
Wyeomyia_Decamyia_pseudopecten	Culicinae	0.245516792
Wyeomyia_Dendromyia_testei	Culicinae	0.245516792
Wyeomyia_D_luteoventralis	Culicinae	0.245516792
Wyeomyia_Dendromyia_ypsipola	Culicinae	0.245516792
Wyeomyia_occulta	Culicinae	0.245516792
Wyeomyia_Prosopolepis_confusa	Culicinae	0.245516792
Wyeomyia_Wyeomyia_sabethea	Culicinae	0.245516792
Wyeomyia_clasoleuca	Culicinae	0.245516792
Wyeomyia_phroso	Culicinae	0.245516792
Sabethes_Sabethes_cyaneus	Culicinae	0.245516792
Sabethes_S_chloropterus	Culicinae	0.245516792
Sabethes_Sabethinus_intermedius	Culicinae	0.245516792
Isostomyia_perturbans	Culicinae	0.245516792
Runchomyia_Ctenogoeldia_magna	Culicinae	0.245516792
Runchomyia_Runchomyia_reversa	Culicinae	0.245516792
Culex_Culex_stigmatosoma	Culicinae	0.245516792
Johnbelkinia_longipes	Culicinae	0.245516792
Shannoniana_fluviatilis	Culicinae	0.245516792
Shannoniana_moralesi	Culicinae	0.245516792
Trichoprosopon_digitatum	Culicinae	0.245516792
Trichoprosopon_pallidiventer	Culicinae	0.245516792
Kimia_decorabilis	Culicinae	0.245516792
Tripteroides_Rachisoura_adentat	Culicinae	0.245516792
Tripteroides_R_mathesoni	Culicinae	0.245516792
Tripteroides_Rach_fuscipleura	Culicinae	0.245516792
Tripteroides_Rachisoura_stonei	Culicinae	0.245516792
Tripteroides_R_brevirhynchus	Culicinae	0.245516792
Tripteroides_T_similis	Culicinae	0.245516792
Tripteroides_T_tarsalis	Culicinae	0.245516792
Wyeomyia_Phoniomyia_splendida	Culicinae	0.245516792
Malaya_genurostris	Culicinae	0.245516792
Onirion_personatum	Culicinae	0.245516792
Onirion_aenigma	Culicinae	0.245516792
Onirion_regale	Culicinae	0.245516792
Onirion_brucei	Culicinae	0.245516792
Onirion_impuris	Culicinae	0.245516792
Onirion_celatum	Culicinae	0.245516792
Malaya_jacobsoni	Culicinae	0.245516792
Onirion_sirivanakarni	Culicinae	0.245516792
Topomyia_Suaymyia_yanbarensis	Culicinae	0.245516792
Topomyia_Suaymyia_houghtoni	Culicinae	0.245516792
Aedes_Protomacleaya_zoosophus	Culicinae	0.245516792

Acartomyia_zammitii	Culicinae	0.245516792
Ochlerotatus_theobaldi	Culicinae	0.245516792
Ochlerotatus_C_fulvus	Culicinae	0.245516792
Ochlerotatus_procax	Culicinae	0.245516792
Culex_Culex_murrelli	Culicinae	0.245516792
Ochlerotatus_thibaulti	Culicinae	0.245516792
Ochlerotatus_Protoculex_atlanti	Culicinae	0.245516792
Ochlerotatus_atlanticus	Culicinae	0.245516792
Ochlerotatus_P_tormentor	Culicinae	0.245516792
Ochlerotatus_Protoculex_dupreei	Culicinae	0.245516792
Ochlerotatus_serratus_serratus	Culicinae	0.245516792
Ochlerotatus_Protoculex_pertina	Culicinae	0.245516792
Ochlerotatus_Chry_fulvus_fulvus	Culicinae	0.245516792
Ochlerotatus_Chry_fulvus_pallen	Culicinae	0.245516792
Ochlerotatus_scapu_confirmatus	Culicinae	0.245516792
Ochlerotatus_O_infirmatus	Culicinae	0.245516792
Ochlerotatus_Ochlerotatus_crini	Culicinae	0.245516792
Ochlerotatus_Buvirilia_edgari	Culicinae	0.245516792
Sallumia_hortator	Culicinae	0.245516792
Levua_geoskusea	Culicinae	0.245516792
Rhinoskusea_longirostris	Culicinae	0.245516792
Rhinoskusea_wardi	Culicinae	0.245516792
Geoskusea_baisasi	Culicinae	0.245516792
Geoskusea_longiforceps	Culicinae	0.245516792
Cancraedes_masculinus	Culicinae	0.245516792
Catageiomyia_irritans	Culicinae	0.245516792
Catageiomyia_tarsalis	Culicinae	0.245516792
Aedimorphus_argenteopunctatus	Culicinae	0.245516792
Elpeytonius_simulans	Culicinae	0.245516792
Elpeytonius_apicoannulatus	Culicinae	0.245516792
Downsiomyia_nipponicus	Culicinae	0.245516792
Ochlerotatus_dufouri	Culicinae	0.245516792
Ochlerotatus_R_provocans	Culicinae	0.245516792
Ochlerotatus_tahoensis	Culicinae	0.245516792
Ochlerotatus_C_cana_canadensis	Culicinae	0.245516792
Ochlerotatus_Woodius_intrudens	Culicinae	0.245516792
Ochlerotatus_Woodius_diantaeus	Culicinae	0.245516792
Ochlerotatus_ratcliffei	Culicinae	0.245516792
Ochlerotatus_communis	Culicinae	0.245516792
Ochlerotatus_pionips	Culicinae	0.245516792
Ochlerotatus_punctor	Culicinae	0.245516792
Ochlerotatus_andersoni	Culicinae	0.245516792
Ochlerotatus_abserratus	Culicinae	0.245516792
Ochlerotatus_implicatus	Culicinae	0.245516792
Ochlerotatus_cataphylla	Culicinae	0.245516792
Ochlerotatus_cantator	Culicinae	0.245516792
Ochlerotatus_riparius	Culicinae	0.245516792
Ochlerotatus_euedes	Culicinae	0.245516792
Ochlerotatus_stimulans	Culicinae	0.245516792
Ochlerotatus_cantans	Culicinae	0.245516792
Ochlerotatus_annulipes	Culicinae	0.245516792
Ochlerotatus_excrucians	Culicinae	0.245516792
Ochlerotatus_calumnior	Culicinae	0.245516792
Ochlerotatus_Empihals_vigilax	Culicinae	0.245516792
Ochlerotatus_C_mitchellae	Culicinae	0.245516792
Ochlerotatus_C_taeniorhynchus	Culicinae	0.245516792
Ochlerotatus_Gilesia_aculeatus	Culicinae	0.245516792
Aedes_Protomacleaya_hendersoni	Culicinae	0.245516792

Ochlerotatus_Gilesia_mcdonaldi	Culicinae	0.245516792
Ochlerotatus_P_flavifrons	Culicinae	0.245516792
Ochlerotatus_P_calcariae	Culicinae	0.245516792
Ochlerotatus_churchillensis	Culicinae	0.245516792
Ochlerotatus_pullatus	Culicinae	0.245516792
Ochlerotatus_Rusticoidus_refiki	Culicinae	0.245516792
Ochlerotatus_R_rusticus	Culicinae	0.245516792
Ochlerotatus_detritus	Culicinae	0.245516792
Ochlerotatus_melanimon	Culicinae	0.245516792
Ochlerotatus_caspius	Culicinae	0.245516792
Ochlerotatus_Rusticoidus_bicris	Culicinae	0.245516792
Acartomyia_mariae	Culicinae	0.245516792
Ochlerotatus_ventrovittis	Culicinae	0.245516792
Ochlerotatus_dorsalis	Culicinae	0.245516792
Ochlerotatus_albisfasciatus	Culicinae	0.245516792
Ochlerotatus_impiger	Culicinae	0.245516792
Ochlerotatus_hexodontus	Culicinae	0.245516792
Ochlerotatus_spencerii_spenceri	Culicinae	0.245516792
Ochlerotatus_Juppius_caballus	Culicinae	0.245516792
Ochlerotatus_L_spilotus	Culicinae	0.245516792
Ochlerotatus_flavescens	Culicinae	0.245516792
Ochlerotatus_kasachstanicus	Culicinae	0.245516792
Ochlerotatus_fitchii	Culicinae	0.245516792
Ochlerotatus_grossbecki	Culicinae	0.245516792
Aedes_Protomacleaya_brelandi	Culicinae	0.245516792
Aedes_Protomacleaya_triseriatus	Culicinae	0.245516792
Ochlerotatus_O_trivittatus	Culicinae	0.245516792
Ochlerotatus_sticticus	Culicinae	0.245516792
Ochlerotatus_nivalis	Culicinae	0.245516792
Ochlerotatus_C_sollicitans	Culicinae	0.245516792
Ochlerotatus_C_nigromaculis	Culicinae	0.245516792
Ochlerotatus_P_postspiraculosus	Culicinae	0.245516792
Ochlerotatus_P_bancroftianus	Culicinae	0.245516792
Mucidus_Mucidus_laniger	Culicinae	0.245516792
Rhinoskusea_portonovoensis	Culicinae	0.245516792
Mucidus_Mucidus_alternans	Culicinae	0.245516792
Ochlerotatus_Coetzeemyia_fryeri	Culicinae	0.245516792
Mucidus_P_aurantius_aurantius	Culicinae	0.245516792
Mucidus_Pardomyia_quadripunctis	Culicinae	0.245516792
Phagomyia_watasei	Culicinae	0.245516792
Himalaius_gilli	Culicinae	0.245516792
Bruceharrisonius_greenii	Culicinae	0.245516792
Bruceharrisonius_alektorovi	Culicinae	0.245516792
Bruceharrisonius_okinawanus	Culicinae	0.245516792
Bruceharrisonius_taiwanus	Culicinae	0.245516792
Ochlerotatus_Protomacleaya_knab	Culicinae	0.245516792
Georgecraigius_G_atropalpus	Culicinae	0.245516792
Georgecraigius_G_epactius	Culicinae	0.245516792
Georgecraigius_H_fluviatilis	Culicinae	0.245516792
Patmarksia_papuensis	Culicinae	0.245516792
Patmarksia_argyronotum	Culicinae	0.245516792
Patmarksia_subalbitarsis	Culicinae	0.245516792
Patmarksia_argenteitarsis	Culicinae	0.245516792
Dobrotworskyius_alboannulatus	Culicinae	0.245516792
Dobrotworskyius_tubbutiensis	Culicinae	0.245516792
Dobrotworskyius_rubrithorax	Culicinae	0.245516792
Collessius_Collessius_hatorii	Culicinae	0.245516792
Collessius_A_tonkinensis	Culicinae	0.245516792

Hulecoeteomyia_formosensis Culicinae 0.245516792
Hulecoeteomyia_japon_japonicus Culicinae 0.245516792
Hulecoeteomyia_j_amamiensis Culicinae 0.245516792
Hulecoeteomyia_koreicus Culicinae 0.245516792
Hulecoeteomyia_j_yayaemensis Culicinae 0.245516792
Hulecoeteomyia_j_shintienensis Culicinae 0.245516792
Armigeres_Armigeres_jugraensis Culicinae 0.245516792
Hulecoeteomyia_chrysolineatus Culicinae 0.245516792
Hulecoeteomyia_sherki Culicinae 0.245516792
Gilesius_pulchriventer Culicinae 0.245516792
Collessius_C_macfarlanei Culicinae 0.245516792
Collessius_Collessius_elsiae Culicinae 0.245516792
Alloeomyia_pseudotaeniatus Culicinae 0.245516792
Collessius_Alloeomyia_banksi Culicinae 0.245516792
Tanakaius_togoi Culicinae 0.245516792
Tanakaius_savoryi Culicinae 0.245516792
Rampamyia_notoscripta Culicinae 0.245516792
Rampamyia_albilabris Culicinae 0.245516792
Haemagogus_H_spegazzinii Culicinae 0.245516792
Ochlerotatus_Proto_triseriatus Culicinae 0.245516792
Haemagogus_mesodentatus Culicinae 0.245516792
Downsiomyia_novoniveus Culicinae 0.245516792
Downsiomyia_nivea Culicinae 0.245516792
Downsiomyia_leonis Culicinae 0.245516792
Haemagogus_Haemagogus_splendens Culicinae 0.245516792
Haemagogus_Haemagogus_equinus Culicinae 0.245516792
Haemagogus_C_leucocelaenus Culicinae 0.245516792
Haemagogus_Cono_leucotaeniatus Culicinae 0.245516792
Finlaya_poicilius Culicinae 0.245516792
Finlaya_kochi Culicinae 0.245516792
Downsiomyia_omorii Culicinae 0.245516792
Howardina_fulvithorax Culicinae 0.245516792
Howardina_walkeri Culicinae 0.245516792
Howardina_sexlineata Culicinae 0.245516792
Dahliana_geniculata Culicinae 0.245516792
Dahliana_echinus Culicinae 0.245516792
Haemagogus_H_janthinomys Culicinae 0.245516792
Ochlerotatus_P_hendersoni Culicinae 0.245516792
Ochlerotatus_P_brelandi Culicinae 0.245516792
Aedes_oreophilus Culicinae 0.245516792
Ochlerotatus_P_burgeri Culicinae 0.245516792
Ochlerotatus_Protomacleaya_komp Culicinae 0.245516792
Ochlerotatus_P_homoeopus Culicinae 0.245516792
Ochlerotatus_P_berlini Culicinae 0.245516792
Ochlerotatus_P_terrens Culicinae 0.245516792
Ochlerotatus_P_galindoi Culicinae 0.245516792
Orthopodomyia_anopheloides Culicinae 0.245516792
Lewnielsenius_muelleri Culicinae 0.245516792
Abraedes_papago Culicinae 0.245516792
Aztecaedes_ramirezi Culicinae 0.245516792
Kompia_purpureipes Culicinae 0.245516792
Gymnometopa_mediovittata Culicinae 0.245516792
Orthopodomyia_fascipes Culicinae 0.245516792
Orthopodomyia_pulcripalpis Culicinae 0.245516792
Culiseta_A_longiareolata Culicinae 0.245516792
Ochlerotatus_P_zoosophus Culicinae 0.245516792
Culiseta_Culiseta_annulata Culicinae 0.245516792
Hopkinsius_Hopkinsius_ingrami Culicinae 0.245516792

Hopkinsius_Hopkinsius_embuensis	Culicinae	0.245516792
Hopkinsius_Yamada_albocinctus	Culicinae	0.245516792
Hopkinsius_Yamada_seoulensis	Culicinae	0.245516792
Phagomyia_gubernatoris	Culicinae	0.245516792
Phagomyia_assamensis	Culicinae	0.245516792
Phagomyia_prominens	Culicinae	0.245516792
Bruceharrisonius_aureostriatus	Culicinae	0.245516792
Ochlerotatus_camptorhynchus	Culicinae	0.245516792
Jihlienius_chungi	Culicinae	0.245516792
Molpemyia_pecuniosa	Culicinae	0.245516792
Luius_fengi	Culicinae	0.245516792
Macleaya_C_wattensis	Culicinae	0.245516792
Macleaya_Macleaya_tremula	Culicinae	0.245516792
Kenknightia_biocellatus	Culicinae	0.245516792
Aedes_crossi	Culicinae	0.245516792
Mucidus_Mucidus_grahamii	Culicinae	0.245516792
Kenknightia_dissimilis	Culicinae	0.245516792
Kenknightia_harbachi	Culicinae	0.245516792
Zavortinkius_fulgens	Culicinae	0.245516792
Zavortinkius_longipalpis	Culicinae	0.245516792
Vansomerenis_pulchrithorax	Culicinae	0.245516792
Vansomerenis_luteostriatus	Culicinae	0.245516792
Aedes_keefei	Culicinae	0.245516792
Aedes_candidoscutellum	Culicinae	0.245516792
Ochlerotatus_Woodius_sticticus	Culicinae	0.245516792
Neomelaniconion_circumluteolus	Culicinae	0.245516792
Aedes_sp1	Culicinae	0.245516792
Ochlerotatus_P_zavortinki	Culicinae	0.245516792
Albuginosus_capensis	Culicinae	0.245516792
Dendroskusea_periskelata	Culicinae	0.245516792
Dendroskusea_microptera	Culicinae	0.245516792
Dendroskusea_reginae	Culicinae	0.245516792
Stegomyia_Actinothrix_edwardsi	Culicinae	0.245516792
Stegomyia_Zoromorphus_futunae	Culicinae	0.245516792
Stegomyia_Bohartius_saipanensis	Culicinae	0.245516792
Stegomyia_Bohartius_pandani	Culicinae	0.245516792
Stegomyia_marshallensis	Culicinae	0.245516792
Stegomyia_hakanssoni	Culicinae	0.245516792
Stegomyia_guamensis	Culicinae	0.245516792
Stegomyia_w_alba	Culicinae	0.245516792
Stegomyia_gardnerii_gardnerii	Culicinae	0.245516792
Stegomyia_Xyele_desmotes	Culicinae	0.245516792
Stegomyia_H_mediopunctata	Culicinae	0.245516792
Stegomyia_subalbopicta	Culicinae	0.245516792
Stegomyia_Huangmyia_perplexa	Culicinae	0.245516792
Stegomyia_Heter_annandalei	Culicinae	0.245516792
Cancraedes_indonesiae	Culicinae	0.245516792
Stegomyia_Heteraspidion_craggi	Culicinae	0.245516792
Stegomyia_flavopicta_flavopicta	Culicinae	0.245516792
Stegomyia_flavopicta_miyarai	Culicinae	0.245516792
Stegomyia_flavopicta_downsi	Culicinae	0.245516792
Stegomyia_katherinensis	Culicinae	0.245516792
Stegomyia_albopicta	Culicinae	0.245516792
Stegomyia_maehleri	Culicinae	0.245516792
Stegomyia_riversi	Culicinae	0.245516792
Stegomyia_dybasi	Culicinae	0.245516792
Stegomyia_palauensis	Culicinae	0.245516792
Stegomyia_scutellaris	Culicinae	0.245516792

Stegomyia_hensilli	Culicinae	0.245516792
Stegomyia_chemulpoensis	Culicinae	0.245516792
Stegomyia_wadai	Culicinae	0.245516792
Stegomyia_poweri	Culicinae	0.245516792
Stegomyia_Mukwaya_bromeliae	Culicinae	0.245516792
Stegomyia_Mukwaya_woodi	Culicinae	0.245516792
Stegomyia_Mukwaya_simpsoni	Culicinae	0.245516792
Stegomyia_Mukwaya_mascarensis	Culicinae	0.245516792
Stegomyia_apicoargentea	Culicinae	0.245516792
Stegomyia_Stegomyia_pia	Culicinae	0.245516792
Stegomyia_Stegomyia_aegypti	Culicinae	0.245516792
Stegomyia_dendrophila	Culicinae	0.245516792
Stegomyia_deboeri	Culicinae	0.245516792
Stegomyia_luteocephala	Culicinae	0.245516792
Stegomyia_africana	Culicinae	0.245516792
Stegomyia_metallica	Culicinae	0.245516792
Heizmannia_proxima	Culicinae	0.245516792
Scutomyia_arboricola	Culicinae	0.245516792
Scutomyia_albolineata	Culicinae	0.245516792
Heizmannia_reidi	Culicinae	0.245516792
Heizmannia_chengi	Culicinae	0.245516792
Heizmannia_menglianensis	Culicinae	0.245516792
Heizmannia_lii	Culicinae	0.245516792
Cornetius_cozi	Culicinae	0.245516792
Lorrainea_dasyorrhus	Culicinae	0.245516792
Lorrainea_amesii	Culicinae	0.245516792
Udaya_subsimilis	Culicinae	0.245516792
Udaya_lucaris	Culicinae	0.245516792
Zeugnomyia_lawtoni	Culicinae	0.245516792
Zeugnomyia_gracilis	Culicinae	0.245516792
Heizmannia_M_catesi	Culicinae	0.245516792
Heizmannia_Mattinglyia_achaetae	Culicinae	0.245516792
Heizmannia_H_scintillans	Culicinae	0.245516792
Heizmannia_Heizmannia_complex	Culicinae	0.245516792
Armigeres_L_longipalpis	Culicinae	0.245516792
Armigeres_Leicesteria_flavus	Culicinae	0.245516792
Armigeres_Armigeres_breinli	Culicinae	0.245516792
Armigeres_Armigeres_subalbatus	Culicinae	0.245516792
Eretmapodites_semisimplicipes	Culicinae	0.245516792
Eretmapodites_quinquevittatus	Culicinae	0.245516792
Alanstonea_brevitibia	Culicinae	0.245516792
Pseudarmigeres_argenteo_dunni	Culicinae	0.245516792
Pseudarmigeres_michaelikati	Culicinae	0.245516792
Petermattinglyius_A_whartoni	Culicinae	0.245516792
Petermattinglyius_P_franciscoi	Culicinae	0.245516792
Petermattinglyius_P_iyengari	Culicinae	0.245516792
Petermattinglyius_P_scanloni	Culicinae	0.245516792
Diceromyia_furcifer	Culicinae	0.245516792
Diceromyia_taylori	Culicinae	0.245516792
Borichinda_B_cavernicola	Culicinae	0.245516792
Stegomyia_galloisi	Culicinae	0.245516792
Aedimorphus_cumminsii	Culicinae	0.245516792
Aedimorphus_vexans_vexans	Culicinae	0.245516792
Aedimorphus_vexans_nipponii	Culicinae	0.245516792
Aedimorphus_vexans_arabiensis	Culicinae	0.245516792
Aedimorphus_dentatus	Culicinae	0.245516792
Aedimorphus_ochraceus	Culicinae	0.245516792
Aedimorphus_trimaculatus	Culicinae	0.245516792

Aedimorphus_caecus	Culicinae	0.245516792
Aedimorphus_orbitae	Culicinae	0.245516792
Aedimorphus_mediolineatus	Culicinae	0.245516792
Aedimorphus_pampangensis	Culicinae	0.245516792
Aedimorphus_eritreae	Culicinae	0.245516792
Aedimorphus_dalzieli	Culicinae	0.245516792
Aedimorphus_culicinus	Culicinae	0.245516792
Aedimorphus_alboscutellatus	Culicinae	0.245516792
Aedimorphus_pallidostriatus	Culicinae	0.245516792
Aedimorphus_taeniorhynchoides	Culicinae	0.245516792
Aedimorphus_pipersalatus	Culicinae	0.245516792
Aedimorphus_domesticus	Culicinae	0.245516792
Aedimorphus_punctifemoris	Culicinae	0.245516792
Aedimorphus_gibbinsi	Culicinae	0.245516792
Aedimorphus_quadrivittatus	Culicinae	0.245516792
Edwardsaedes_imprimens	Culicinae	0.245516792
Edwardsaedes_bekkui	Culicinae	0.245516792
Neomelaniconion_palpale	Culicinae	0.245516792
Neomelaniconion_lineatopennis	Culicinae	0.245516792
Verrallina_Harbachius_yusafi	Culicinae	0.245516792
Verrallina_Harbachius_nobukonis	Culicinae	0.245516792
Verrallina_Verrallina_butleri	Culicinae	0.245516792
Verrallina_Verrallina_carmenti	Culicinae	0.245516792
Verrallina_N_pseudomediofasciat	Culicinae	0.245516792
Verrallina_Neomacleaya_indica	Culicinae	0.245516792
Paraedes_barraudi	Culicinae	0.245516792
Paraedes_ostentatio	Culicinae	0.245516792
Aedes_esoensis	Culicinae	0.245516792
Aedes_cinereus	Culicinae	0.245516792
Aedimorphus_albocephalus	Culicinae	0.245516792
Bifidistylus_lamborni	Culicinae	0.245516792
Albuginosus_ngong	Culicinae	0.245516792
Albuginosus_marshallii	Culicinae	0.245516792
Christophersiomyia_gombakensis	Culicinae	0.245516792
Christophersiomyia_thomsoni	Culicinae	0.245516792
Huaedes_wauensis	Culicinae	0.245516792
Tewarius_Agastyai_reubenae	Culicinae	0.245516792
Tewarius_Agastyai_agastyai	Culicinae	0.245516792
Skusea_pembaensis	Culicinae	0.245516792
Indusius_pulverulentus	Culicinae	0.245516792
Cancraedes_cancricomes	Culicinae	0.245516792
Cancraedes_penghuensis	Culicinae	0.245516792
Fredwardsius_vittatus	Culicinae	0.245516792
Isoaedes_cavaticus	Culicinae	0.245516792
Jarnellius_Jarnellius_varipalpu	Culicinae	0.245516792
Jarnellius_deserticola	Culicinae	0.245516792
Jarnellius_laguna	Culicinae	0.245516792
Jarnellius_monticola	Culicinae	0.245516792
Halaedes_ashworthi	Culicinae	0.245516792
Halaedes_wardangensis	Culicinae	0.245516792
Nothoskusea_Nothoskusea_chatham	Culicinae	0.245516792
Opifex_Opifex_fuscus	Culicinae	0.245516792
Psorophora_Grabhamia_columbiae	Culicinae	0.245516792
Psorophora_Grabhamia_signipenni	Culicinae	0.245516792
Psorophora_J_longipalpus	Culicinae	0.245516792
Psorophora_Psorophora_ciliata	Culicinae	0.245516792
Psorophora_Psorophora_howardii	Culicinae	0.245516792
Psorophora_Janthinosoma_ferox	Culicinae	0.245516792

Psorophora_Grabhamia_cingulata	Culicinae	0.245516792
Psorophora_Jan_cyanescens	Culicinae	0.245516792
Psorophora_Jan_confinnis	Culicinae	0.245516792
Mimomyia_Mimomyia_chamberlaini	Culicinae	0.245516792
Eretmapodites_intermedius	Culicinae	0.245516792
Wyeomyia_sp1	Culicinae	0.245516792
Wyeomyia_negrensis	Culicinae	0.245516792
Wyeomyia_chalcocephala	Culicinae	0.245516792
Wyeomyia_Dendromyia_complosa	Culicinae	0.245516792
Coquillettidia_C_maculipennis	Culicinae	0.245516792
Coquillettidia_C_crassipes	Culicinae	0.245516792
Coquillettidia_R_venezuelensis	Culicinae	0.245516792
Heizmannia_Heizmannia_chandi	Culicinae	0.245516792
Heizmannia_Mattinglyia_discrepa	Culicinae	0.245516792
Mansonia_Mansonioides_africana	Culicinae	0.245516792
Mansonia_Mansonioides_bonneae	Culicinae	0.245516792
Mansonia_Mansonia_titillans	Culicinae	0.245516792
Mansonia_Mansonia_flaveola	Culicinae	0.245516792
Mansonia_Mansonioides_uniformis	Culicinae	0.245516792
Mansonia_Mansonioides_annulifer	Culicinae	0.245516792
Mimomyia_Etorleptiomyia_elegans	Culicinae	0.245516792
Mimomyia_Mimomyia_plumosa	Culicinae	0.245516792
Ficalbia_minima	Culicinae	0.245516792
Ficalbia_malfeyti	Culicinae	0.245516792
Culex_Culex_minor	Culicinae	0.245516792
Uranotaenia_U_pallidocephala	Culicinae	0.245516792
Uranotaenia_P_colocasiae	Culicinae	0.245516792
Culex_Melanoconion_inhibitator	Culicinae	0.245516792
Mansonia_Mansonioides_annulata	Culicinae	0.245516792
Coquillettidia_C_richiardii	Culicinae	0.245516792
Coquillettidia_C_perturbans	Culicinae	0.245516792
Aedeomyia_Aedeomyia_squamipenni	Culicinae	0.245516792
Mimomyia_E_luzonensis	Culicinae	0.245516792
Culiseta_Culiseta_bergrothi	Culicinae	0.245516792
Culiseta_Culiseta_inornata	Culicinae	0.245516792
Culiseta_Culicella_nipponica	Culicinae	0.245516792
Culiseta_Culiseta_impatiens	Culicinae	0.245516792
Culiseta_Culicella_ochroptera	Culicinae	0.245516792
Culiseta_Climacura_melanura	Culicinae	0.245516792
Culiseta_Culiseta_morsitans	Culicinae	0.245516792
Culiseta_Culiseta_minnesotae	Culicinae	0.245516792
Wyeomyia_Antunesmyia_alani	Culicinae	0.245516792
Wyeomyia_surinamensis	Culicinae	0.245516792
Wyeomyia_flui	Culicinae	0.245516792
Wyeomyia_Phoniomyia_palmata	Culicinae	0.245516792
Wyeomyia_Dendromyia_jocosa	Culicinae	0.245516792
Wyeomyia_Spilonympha_howardi	Culicinae	0.245516792
Wyeomyia_Phoniomyia_edwardsi	Culicinae	0.245516792
Wyeomyia_Wyeomyia_limai	Culicinae	0.245516792
Wyeomyia_Triamyia_staminifera	Culicinae	0.245516792
Anopheles_Anopheles_daciae	Anophelinae	0.520491803
Anopheles_Lophopodomyia_gilesi	Anophelinae	0.520491803
Anopheles_Anopheles_forattinii	Anophelinae	0.520491803
Anopheles_Anopheles_minor	Anophelinae	0.520491803
Anopheles_A_quadrimaculatus	Anophelinae	0.520491803
Anopheles_Anopheles_xui	Anophelinae	0.520491803
Stegomyia_cretina	Culicinae	0.245516792
Anopheles_Anopheles_claviger	Anophelinae	0.520491803

Anopheles_Cellia_maculipalpis	Anophelinae	0.520491803
Anopheles_Anopheles_asiaticus	Anophelinae	0.520491803
Anopheles_Cellia_pretoriensis	Anophelinae	0.520491803
Anopheles_Anopheles_plumbeus	Anophelinae	0.520491803
Anopheles_Anopheles_interruptus	Anophelinae	0.520491803
Anopheles_L_oiketorakras	Anophelinae	0.520491803
Anopheles_Cellia_quadrimaculatu	Anophelinae	0.520491803
Anopheles_A_pseudobarbirostris	Anophelinae	0.520491803
Anopheles_Anopheles_earlei	Anophelinae	0.520491803
Anopheles_Anopheles_messeae	Anophelinae	0.520491803
Anopheles_Anopheles_persiensis	Anophelinae	0.520491803
Anopheles_Anopheles_artemievi	Anophelinae	0.520491803
Anopheles_Anopheles_barbumbrosu	Anophelinae	0.520491803
Anopheles_Anopheles_melanoon	Anophelinae	0.520491803
Anopheles_Anopheles_bancroftii	Anophelinae	0.520491803
Anopheles_Cellia_somalicus	Anophelinae	0.520491803
Anopheles_Anopheles_maculipenni	Anophelinae	0.520491803
Anopheles_Anopheles_barbirostri	Anophelinae	0.520491803
Anopheles_Anopheles_freyi	Anophelinae	0.520491803
Anopheles_Anopheles_koreicus	Anophelinae	0.520491803
Anopheles_Cellia_aitkenii	Anophelinae	0.520491803
Anopheles_Anopheles_pollicaris	Anophelinae	0.520491803
Anopheles_Anopheles_coustani	Anophelinae	0.520491803
Anopheles_Anopheles_crawfordi	Anophelinae	0.520491803
Anopheles_Anopheles_tenebrosus	Anophelinae	0.520491803
Anopheles_Anopheles_sinensis	Anophelinae	0.520491803
Anopheles_Anopheles_belenrae	Anophelinae	0.520491803
Anopheles_Anopheles_engarensis	Anophelinae	0.520491803
Anopheles_Anopheles_kleini	Anophelinae	0.520491803
Anopheles_Anopheles_lesteri	Anophelinae	0.520491803
Anopheles_A_anthropophagus	Anophelinae	0.520491803
Anopheles_Anopheles_sineroides	Anophelinae	0.520491803
Anopheles_A_kweiyangensis	Anophelinae	0.520491803
Anopheles_A_liangshanensis	Anophelinae	0.520491803
Anopheles_Anopheles_kunmingensi	Anophelinae	0.520491803
Anopheles_Anopheles_hyrcanus	Anophelinae	0.520491803
Anopheles_junlianensis	Anophelinae	0.520491803
Anopheles_yatsushiroensis	Anophelinae	0.520491803
Anopheles_Anopheles_pullus	Anophelinae	0.520491803
Anopheles_A_peditaeniatus	Anophelinae	0.520491803
Anopheles_Anopheles_argyropus	Anophelinae	0.520491803
Anopheles_Anopheles_nigerrimus	Anophelinae	0.520491803
Anopheles_Anopheles_nitidus	Anophelinae	0.520491803
Anopheles_Anopheles_pursati	Anophelinae	0.520491803
Anopheles_Anopheles_grabhamii	Anophelinae	0.520491803
Anopheles_Anopheles_punctimacul	Anophelinae	0.520491803
Anopheles_Anopheles_shannoni	Anophelinae	0.520491803
Anopheles_A_mediopunctatus	Anophelinae	0.520491803
Anopheles_Anopheles_aztecus	Anophelinae	0.520491803
Anopheles_Anopheles_intermedius	Anophelinae	0.520491803
Anopheles_Anopheles_montanus	Anophelinae	0.520491803
Anopheles_A_albotaeniatus	Anophelinae	0.520491803
Anopheles_Anopheles_umbrosus	Anophelinae	0.520491803
Anopheles_Anopheles_baezai	Anophelinae	0.520491803
Anopheles_Anopheles_letifer	Anophelinae	0.520491803
Anopheles_Anopheles_punctipenni	Anophelinae	0.520491803
Anopheles_Anopheles_crucians	Anophelinae	0.520491803
Anopheles_Anopheles_martinius	Anophelinae	0.520491803

Anopheles_Anopheles_sacharovi	Anophelinae	0.520491803
Anopheles_Anopheles_occidentali	Anophelinae	0.520491803
Anopheles_A_neomaculipalpus	Anophelinae	0.520491803
Anopheles_Anopheles_hermsi	Anophelinae	0.520491803
Anopheles_Anopheles_freeborni	Anophelinae	0.520491803
Anopheles_Anopheles_atropos	Anophelinae	0.520491803
Anopheles_C_quadriannulatus	Anophelinae	0.520491803
Anopheles_Anopheles_beklemishev	Anophelinae	0.520491803
Anopheles_Cellia_merus	Anophelinae	0.520491803
Anopheles_Cellia_bwambae	Anophelinae	0.520491803
Anopheles_Cellia_melas	Anophelinae	0.520491803
Anopheles_Anopheles_eiseni	Anophelinae	0.520491803
Anopheles_Anopheles_lindesayi	Anophelinae	0.520491803
Anopheles_Anopheles_labranchiae	Anophelinae	0.520491803
Anopheles_Anopheles_atroparvus	Anophelinae	0.520491803
Anopheles_Cellia_rufipes	Anophelinae	0.520491803
Anopheles_A_pseudopunctipennis	Anophelinae	0.520491803
Anopheles_Anopheles_hectoris	Anophelinae	0.520491803
Anopheles_Anopheles_pseudopictu	Anophelinae	0.520491803
Anopheles_St_acanthotorynus	Anophelinae	0.520491803
Anopheles_Cellia_yaeyamaensis	Anophelinae	0.520491803
Anopheles_Anopheles_saperoi	Anophelinae	0.520491803
Anopheles_Cellia_tessellatus	Anophelinae	0.520491803
Anopheles_Cellia_ardensis	Anophelinae	0.520491803
Anopheles_Cellia_leucosphyrus	Anophelinae	0.520491803
Anopheles_Cellia_latens	Anophelinae	0.520491803
Anopheles_Cellia_macarthuri	Anophelinae	0.520491803
Anopheles_Cellia_sulawesi	Anophelinae	0.520491803
Anopheles_Cellia_mirans	Anophelinae	0.520491803
Anopheles_Cellia_nemophilous	Anophelinae	0.520491803
Anopheles_Cellia_scanloni	Anophelinae	0.520491803
Anopheles_Cellia_cracens	Anophelinae	0.520491803
Anopheles_Cellia_takasagoensis	Anophelinae	0.520491803
Anopheles_Cellia_velegans	Anophelinae	0.520491803
Anopheles_Cellia_dirus	Anophelinae	0.520491803
Anopheles_Cellia_baimaii	Anophelinae	0.520491803
Anopheles_Cellia_balabacensis	Anophelinae	0.520491803
Anopheles_Cellia_kochi	Anophelinae	0.520491803
Anopheles_Cellia_clowi	Anophelinae	0.520491803
Anopheles_Cellia_novaguinensis	Anophelinae	0.520491803
Anopheles_Cellia_koliensis	Anophelinae	0.520491803
Anopheles_Cellia_punctulatus	Anophelinae	0.520491803
Anopheles_Cellia_torresiensis	Anophelinae	0.520491803
Anopheles_Cellia_hinesorum	Anophelinae	0.520491803
Anopheles_Anopheles_peryassui	Anophelinae	0.520491803
Anopheles_Cellia_farauti	Anophelinae	0.520491803
Anopheles_Cellia_irenicus	Anophelinae	0.520491803
Anopheles_Cellia_lungae	Anophelinae	0.520491803
Anopheles_Cellia_solomonis	Anophelinae	0.520491803
Anopheles_Cellia_meraukensis	Anophelinae	0.520491803
Anopheles_Cellia_annulipes	Anophelinae	0.520491803
Anopheles_Cellia_cinereus	Anophelinae	0.520491803
Anopheles_Cellia_mascarensis	Anophelinae	0.520491803
Anopheles_Cellia_distinctus	Anophelinae	0.520491803
Anopheles_Cellia_culicifacies	Anophelinae	0.520491803
Anopheles_Cellia_funestus	Anophelinae	0.520491803
Anopheles_Cellia_vaneedeni	Anophelinae	0.520491803
Anopheles_Cellia_parensis	Anophelinae	0.520491803

Anopheles_Cellia_flavirostris	Anophelinae	0.520491803
Anopheles_Cellia_leesoni	Anophelinae	0.520491803
Anopheles_Cellia_harrisoni	Anophelinae	0.520491803
Anopheles_Cellia_fluviatilis	Anophelinae	0.520491803
Anopheles_Cellia_varuna	Anophelinae	0.520491803
Anopheles_Cellia_pampanai	Anophelinae	0.520491803
Anopheles_Cellia_aconitus	Anophelinae	0.520491803
Anopheles_Cellia_filipinae	Anophelinae	0.520491803
Anopheles_Cellia_jeyporiensis	Anophelinae	0.520491803
Anopheles_Cellia_marshallii	Anophelinae	0.520491803
Anopheles_Cellia_demeilloni	Anophelinae	0.520491803
Anopheles_Cellia_apoci	Anophelinae	0.520491803
Anopheles_Cellia_sergentii	Anophelinae	0.520491803
Anopheles_Cellia_ainshamsi	Anophelinae	0.520491803
Anopheles_Cellia_dthali	Anophelinae	0.520491803
Anopheles_Cellia_ruarinus	Anophelinae	0.520491803
Anopheles_L_pseudotibiamaculatu	Anophelinae	0.520491803
Anopheles_Cellia_fuscivenosus	Anophelinae	0.520491803
Anopheles_Cellia_rivulorum	Anophelinae	0.520491803
Anopheles_Cellia_karwari	Anophelinae	0.520491803
Anopheles_Cellia_superpictus	Anophelinae	0.520491803
Anopheles_Cellia_splendidus	Anophelinae	0.520491803
Anopheles_Cellia_stephensi	Anophelinae	0.520491803
Anopheles_Cellia_pulcherrimus	Anophelinae	0.520491803
Anopheles_Cellia_annularis	Anophelinae	0.520491803
Anopheles_Cellia_pallidus	Anophelinae	0.520491803
Anopheles_Cellia_philippinensis	Anophelinae	0.520491803
Anopheles_Cellia_nivipes	Anophelinae	0.520491803
Anopheles_Cellia_pharoensis	Anophelinae	0.520491803
Anopheles_Cellia_jamesii	Anophelinae	0.520491803
Anopheles_Cellia_pseudojamesi	Anophelinae	0.520491803
Anopheles_Cellia_dravidicus	Anophelinae	0.520491803
Anopheles_Cellia_greeni	Anophelinae	0.520491803
Anopheles_Cellia_dispar	Anophelinae	0.520491803
Anopheles_Cellia_willmori	Anophelinae	0.520491803
Anopheles_Cellia_notanandai	Anophelinae	0.520491803
Anopheles_Cellia_sawadwongporni	Anophelinae	0.520491803
Anopheles_Cellia_pattoni	Anophelinae	0.520491803
Anopheles_Cellia_pseudowillmori	Anophelinae	0.520491803
Anopheles_Cellia_multicolor	Anophelinae	0.520491803
Anopheles_Cellia_sundaicus	Anophelinae	0.520491803
Anopheles_Cellia_indefinitus	Anophelinae	0.520491803
Anopheles_Cellia_limosus	Anophelinae	0.520491803
Anopheles_Cellia_ludlowae	Anophelinae	0.520491803
Anopheles_Cellia_parangensis	Anophelinae	0.520491803
Anopheles_Cellia_arabiensis	Anophelinae	0.520491803
Anopheles_Anopheles_bengalensis	Anophelinae	0.520491803
Anopheles_Anopheles_baileyi	Anophelinae	0.520491803
Anopheles_Cellia_gambiae	Anophelinae	0.520491803
Anopheles_N_argyritarsis	Anophelinae	0.520491803
Anopheles_Nyssorhynchus_darling	Anophelinae	0.520491803
Anopheles_N_triannulatus	Anophelinae	0.520491803
Anopheles_N_atacamensis	Anophelinae	0.520491803
Anopheles_N_albimanus	Anophelinae	0.520491803
Anopheles_N_braziliensis	Anophelinae	0.520491803
Anopheles_N_janconnae	Anophelinae	0.520491803
Anopheles_N_benarrochi	Anophelinae	0.520491803
Anopheles_Anopheles_fluminensis	Anophelinae	0.520491803

Anopheles_Nyssorhynchus_dunhami	Anophelinae	0.520491803
Anopheles_Nyssorhynchus_goeldii	Anophelinae	0.520491803
Anopheles_N_nuneztovari	Anophelinae	0.520491803
Anopheles_Nyssorhynchus_trinkae	Anophelinae	0.520491803
Anopheles_Nyssorhynchus_rangeli	Anophelinae	0.520491803
Anopheles_Nyssorhynchus_evansae	Anophelinae	0.520491803
Anopheles_Nyssorhynchus_konderi	Anophelinae	0.520491803
Anopheles_Nyssorhynchus_oswaldo	Anophelinae	0.520491803
Anopheles_sp1	Anophelinae	0.520491803
Anopheles_Nyssorhynchus_arthuri	Anophelinae	0.520491803
Anopheles_Nyssorhynchus_rondoni	Anophelinae	0.520491803
Anopheles_Nyssorhynchus_strodei	Anophelinae	0.520491803
Anopheles_Cellia_mangyanus	Anophelinae	0.520491803
Anopheles_N_oryzalimnetes	Anophelinae	0.520491803
Anopheles_N_albitarsis	Anophelinae	0.520491803
Anopheles_N_deaneorum	Anophelinae	0.520491803
Anopheles_Cellia_christyi	Anophelinae	0.520491803
Verrallina_Verrallina_dux	Culicinae	0.245516792
Anopheles_Nyssorhynchus_parvus	Anophelinae	0.520491803
Anopheles_Nyssorhynchus_guarani	Anophelinae	0.520491803
Anopheles_Nyssorhynchus_lutzii	Anophelinae	0.520491803
Anopheles_Nyssorhynchus_antunes	Anophelinae	0.520491803
Stegomyia_pseudoscutellaris	Culicinae	0.245516792
Anopheles_Anopheles_lanei	Anophelinae	0.520491803
Anopheles_Kerteszia_laneanus	Anophelinae	0.520491803
Anopheles_Kerteszia_cruzii	Anophelinae	0.520491803
Anopheles_Kerteszia_homunculus	Anophelinae	0.520491803
Anopheles_Kerteszia_bellator	Anophelinae	0.520491803
Anopheles_K_bambusicolus	Anophelinae	0.520491803
Anopheles_Kerteszia_lepidotus	Anophelinae	0.520491803
Anopheles_Kerteszia_pholidotus	Anophelinae	0.520491803
Anopheles_K_gonzalezrinconesi	Anophelinae	0.520491803
Anopheles_Kerteszia_boliviensis	Anophelinae	0.520491803
Anopheles_Kerteszia_rollai	Anophelinae	0.520491803
Anopheles_K_auyantepuiensis	Anophelinae	0.520491803
Anopheles_Kerteszia_neivai	Anophelinae	0.520491803
Stegomyia_polynesiensis	Culicinae	0.245516792
Anopheles_Anopheles_bradleyi	Anophelinae	0.520491803
Anopheles_Anopheles_annulipalpi	Anophelinae	0.520491803
Anopheles_Christya_implexus	Anophelinae	0.520491803
Stegomyia_pernotata	Culicinae	0.245516792
Stegomyia_tongae	Culicinae	0.245516792
Anopheles_L_squamifemur	Anophelinae	0.520491803
Stegomyia_cooki	Culicinae	0.245516792
Stegomyia_kesseli	Culicinae	0.245516792
Anopheles_Cellia_longirostris	Anophelinae	0.520491803
Anopheles_Cellia_amictus	Anophelinae	0.520491803
Anopheles_A_vestitipennis	Anophelinae	0.520491803
Anopheles_Kerteszia_hilli	Anophelinae	0.520491803
Anopheles_A_cucphuongensis	Anophelinae	0.520491803
Anopheles_Stethomyia_kompi	Anophelinae	0.520491803
Anopheles_Stethomyia_nimbus	Anophelinae	0.520491803
Anopheles_Cellia_confusus	Anophelinae	0.520491803
Anopheles_Anopheles_gracilis	Anophelinae	0.520491803
Anopheles_Anopheles_hollandi	Anophelinae	0.520491803
Bironella_Neobironella_confusa	Anophelinae	0.520491803
Bironella_Brugella_hollandi	Anophelinae	0.520491803
Bironella_Bironella_gracilis	Anophelinae	0.520491803

Anopheles_Anopheles_corethroide	Anophelinae	0.520491803
Anopheles_Cellia_nili	Anophelinae	0.520491803
Anopheles_Anopheles_algeriensis	Anophelinae	0.520491803
Anopheles_Anopheles_judithae	Anophelinae	0.520491803
Anopheles_Anopheles_barberi	Anophelinae	0.520491803
Anopheles_Anopheles_aitkenii	Anophelinae	0.520491803
Anopheles_A_insulaeflorum	Anophelinae	0.520491803
Anopheles_Anopheles_alongensis	Anophelinae	0.520491803
Anopheles_Cellia_ovengensis	Anophelinae	0.520491803
Anopheles_Anopheles_culiciformi	Anophelinae	0.520491803
Anopheles_Anopheles_sintonoides	Anophelinae	0.520491803
Anopheles_Cellia_epiroticus	Anophelinae	0.520491803
Anopheles_Cellia_carnevalei	Anophelinae	0.520491803
Anopheles_Cellia_theileri	Anophelinae	0.520491803
Anopheles_Cellia_wellcomei	Anophelinae	0.520491803
Anopheles_Cellia_moucheti	Anophelinae	0.520491803
Anopheles_Anopheles_walkeri	Anophelinae	0.520491803
Chagasia_fajardi	Anophelinae	0.520491803
Chagasia_bathana	Anophelinae	0.520491803

Supplementary References

Part1: Reference list of the 284 source papers and constituent trees analysed to produce our supertree.

1. Alam MT, Das MK, Dev V, Ansari MA, Sharma YD. PCR-RFLP method for the identification of four members of the *Anopheles annularis* group of mosquitoes (Diptera: Culicidae). *Transactions of the Royal Society of Tropical Medicine and Hygiene* 2007, **101**(3)**:** 239-244.

2. Ambrose L, Riginos C, Cooper RD, Leow KS, Ong W, Beebe NW. Population structure, mitochondrial polyphyly and the repeated loss of human biting ability in anopheline mosquitoes from the southwest Pacific. *Molecular ecology* 2012, **21**(17)**:** 4327-4343.

3. anderson JR, Grimstad PR, Severson DW. Chromosomal evolution among six mosquito species (Diptera: Culicidae) based on shared restriction fragment length polymorphisms. *Mol Phylogenet Evol* 2001, **20**(2)**:** 316-321.

4. andreasen MH, Ffrench-Constant RH. In situ hybridization to the Rdl locus on polytene chromosome 3L of Anopheles stephensi. *Medical and Veterinary Entomology* 2002, **16**(4)**:** 452-455.

5. Anthony TG, Harbach RE, Kitching IJ. Phylogeny of the *Pyretophorus* Series of *Anopheles* subgenus *Cellia* (Diptera: Culicidae). *Syst Entomol* 1999, **24**(2)**:** 193-205.

6. Arif-Ul-Hasan, Suguri S, Ahmed SMU, Fujimoto C, Harada M, Rahman SM*, et al.* Molecular phylogeography of *Culex quinquefasciatus* mosquitoes in central Bangladesh. *Acta tropica* 2009, **112**(2)**:** 106-114.

7. Bae YA, Ahn JS, Kim SH, Rhyu MG, Kong Y, Cho SY. PwRn1, a novel Ty3/gypsy-like retrotransposon of *Paragonimus westermani*: molecular characters and its differentially preserved mobile potential according to host chromosomal polyploidy. *BMC genomics* 2008, **9**.

8. Baimai V. Heterochromatin accumulation and karyotypic evolution in some dipteran insects. *Zool Stud* 1998, **37**(2)**:** 75-88.

9. Ballard JWO, Puslednik L, Wolff JN, Russell RC. Variation under nature: A sesquicentennial DNA barcoding perspective. *Chiang Mai J Sci* 2009, **36**(2)**:** 188-199.

10. Banerjee AK, Arora N, Murty US. How far is ITS2 reliable as a phylogenetic marker for the mosquito genera. *Electronic Journal of Biology* 2007, **3**(3)**:** 61-68.

11. Bargues MD, Latorre JM, Morchon R, Simon F, Escosa R, Aranda C*, et al.* rDNA sequences of *Anopheles* species from the Iberian Peninsula and an evaluation of the 18S rRNA gene as

phylogenetic marker in anophelinae. *Journal of Medical Entomology* 2006, **43**(3)**:** 508-517.

12. Beebe NW, Cooper RD. Distribution and evolution of the *Anopheles punctulatus* group (Diptera: Culicidae) in Australia and Papua New Guinea. *International journal for parasitology* 2002, **32**(5)**:** 563-574.

13. Beebe NW, Cooper RD, Morrison DA, Ellis JT. Subset partitioning of the ribosomal DNA small subunit and its effects on the phylogeny of the *Anopheles punctulatus* group. *Insect molecular biology* 2000, **9**(5)**:** 515-520.

14. Beebe NW, Cooper RD, Morrison DA, Ellis JT. A phylogenetic study of the *Anopheles punctulatus* group of malaria vectors comparing rDNA sequence alignments derived from the mitochondrial and nuclear small ribosomal subunits. *Mol Phylogenet Evol* 2000, **17**(3)**:** 430-436.

15. Beebe NW, Ellis JT, Cooper RD, Saul A. DNA sequence analysis of the ribosomal DNA ITS2 region for the *Anopheles punctulatus* group of mosquitoes. *Insect molecular biology* 1999, **8**(3)**:** 381-390.

16. Behbahani A, Dutton TJ, Davies N, Townson H, Sinkins SP. Population differentiation and *Wolbachia* phylogeny in mosquitoes of the *Aedes scutellaris* group. *Medical and Veterinary Entomology* 2005, **19**(1)**:** 66-71.

17. Behura SK, Haugen M, Flannery E, Sarro J, Tessier CR, Severson DW*, et al.* Comparative Genomic Analysis of *Drosophila melanogaster* and vector mosquito developmental genes. *PLoS One* 2011, **6**(7).

18. Besansky NJ. Complexities in the analysis of cryptic taxa within the genus *Anopheles*. *Parassitologia, Vol 41, Nos 1-3, September* 1999**:** 97-100.

19. Besansky NJ, Fahey GT. Utility of the white gene in estimating phylogenetic relationships among mosquitoes (Diptera: Culicidae). *Mol Biol Evol* 1997, **14**(4)**:** 442-454.

20. Besansky NJ, Powell JR, Caccone A, Hamm DM, Scott JA, Collins FH. Molecular Phylogeny of the *Anopheles gambiae* complex suggests genetic introgression between principal malaria vectors. *Proceedings of the National Academy of Sciences of the United States of America* 1994, **91**(15)**:** 6885-6888.

21. Bloem S. Enzymatic variation in the *varipalpus* group of *Aedes (Ochlerotatus)* (Diptera: Culicidae). *Ann Entomol Soc Am* 1991, **84**(3)**:** 217-227.

22. Borkent A. The frog-biting midges of the world (Corethrellidae: Diptera). *Zootaxa* 2008(1804)**:** 1-456.

23. Borkent A. The pupae of Culicomorpha-morphology and a New Phylogenetic Tree. *Zootaxa*

2012(3396): 1-+.

24. Borkent A, Bissett B. A revision of the Holarctic species of *Serromyia* Meigen (Diptera, Ceratopogonidae). *Syst Entomol* 1990, **15**(2): 153-217.

25. Borkent A, Grimaldi DA. The earliest fossil mosquito (Diptera: Culicidae), in mid-Cretaceous Burmese amber. *Ann Entomol Soc Am* 2004, **97**(5): 882-888.

26. Borkent CJ, Borkent A. Description and phylogenetic interpretation of chromatophore migration from larval air sacs to adult structures in some Chaoboridae (Diptera). *Can Entomol* 2008, **140**(6): 630-640.

27. Bourke BP, Foster PG, Bergo ES, Calado DC, Sallum MAM. Phylogenetic relationships among species of *Anopheles (Nyssorhynchus)* (Diptera, Culicidae) based on nuclear and mitochondrial gene sequences. *Acta tropica* 2010, **114**(2): 88-96.

28. Bourke BP, Oliveira TP, Suesdek L, Bergo ES, Sallum MAM. A multi-locus approach to barcoding in the *Anopheles strodei* subgroup (Diptera: Culicidae). *Parasites & vectors* 2013, **6**.

29. Bower JE, Cooper RD, Beebe NW. Internal repetition and intraindividual variation in the rDNA ITS1 of the *Anopheles punctulatus* group (Diptera: Culicidae): Multiple units and rates of turnover. *J Mol Evol* 2009, **68**(1): 66-79.

30. Bower JE, Dowton M, Cooper RD, Beebe NW. Intraspecific Concerted evolution of the rDNA ITS1 in *Anopheles farauti* sensu stricto (Diptera: Culicidae) reveals recent patterns of population structure. *J Mol Evol* 2008, **67**(4): 397-411.

31. Brochero HHL, Li C, Wilkerson RC. A newly recognized species in the *Anopheles (Nyssorhynchus) albitarsis* complex (Diptera: Culicidae) from Puerto Carreno, Colombia. *American Journal of Tropical Medicine and Hygiene* 2007, **76**(6): 1113-1117.

32. Brust RA, Ballard JWO, Driver F, Hartley DM, Galway NJ, Curran J. Molecular systematics, morphological analysis, and hybrid crossing identify a third taxon*, Aedes (Halaedes) wardangensis* sp.nov., of *the Aedes (Halaedes) australis* species-group (Diptera: Culicidae). *Can J Zool* 1998, **76**(7): 1236-1246.

33. Byrd BD, Harrison BA, Zavortink TJ, Wesson DM. Sequence, secondary structure, and phylogenetic analyses of the ribosomal iInternal transcribed spacer 2 (ITS2) in members of the North American signifera group of *Orthopodomyia* (Diptera: Culicidae). *Journal of Medical Entomology* 2012, **49**(6): 1189-1197.

34. Caccone A, Garcia BA, Powell JR. Evolution of the mitochondrial DNA control region in the *Anopheles gambiae* complex. *Insect molecular biology* 1996, **5**(1): 51-59.

35. Caccone A, Min GS, Powell JR. Multiple origins of cytologically identical chromosome inversions in the *Anopheles gambiae* complex. *Genetics* 1998, **150**(2)**:** 807-814.

36. Calado DC, Foster PG, Bergo ES, dos Santos CLS, Galardo AKR, Sallum MAM. Resurrection of *Anopheles goeldii* from synonymy with *Anopheles nuneztovari* (Diptera, Culicidae) and a new record for *Anopheles dunhami* in the Brazilian Amazon. *Memorias do Instituto Oswaldo Cruz* 2008, **103**(8)**:** 791-799.

37. Cameron EC, Wilkerson RC, Mogi M, Miyagi I, Toma T, Kim HC*, et al.* Molecular phylogenetics of *Aedes japonicus*, a disease vector that recently invaded Western Europe, North America, and the Hawaiian Islands. *Journal of Medical Entomology* 2010, **47**(4)**:** 527-535.

38. Chen B, Butlin RK, Harbach RE. Molecular phylogenetics of the Oriental members of the *Myzomyia* Series of *Anopheles* subgenus *Cellia* (Diptera: Culicidae) inferred from nuclear and mitochondrial DNA sequences. *Syst Entomol* 2003, **28**(1)**:** 57-69.

39. Chen B, Butlin RK, Pedro PM, Wang XZ, Harbach RE. Molecular variation, systematics and distribution of the *Anopheles fluviatilis* complex in southern Asia. *Medical and Veterinary Entomology* 2006, **20**(1)**:** 33-43.

40. Chen YY, Lin JW, Fan YC, Tu WC, Chang GJJ, Chiou SS. First detection of the Africa/Caribbean/Latin American subtype of *Culex flavivirus* in Asian country, Taiwan. *Comp Immunol Microb* 2013, **36**(4)**:** 387-396.

41. Choi KS, Koekemoer LL, Coetzee M. Population genetic structure of the major malaria vector *Anopheles funestus* s.s. and allied species in southern Africa. *Parasites & vectors* 2012, **5**.

42. Collucci E, Sallum MAM. Phylogenetic analysis of the subgenus *Kerteszia* of *Anopheles* (Diptera: Culicidae: Anophelinae) based on morphological characters. *Insect Syst Evol* 2003, **34**(4)**:** 361-372.

43. Collucci E, Sallum MAM. Cladistic analysis of the subgenus *Anopheles (Anopheles)* Meigen (Diptera: Culicidae) based on morphological characters. *Memorias do Instituto Oswaldo Cruz* 2007, **102**(3)**:** 277-291.

44. Conn JE, Mitchell SE, Cockburn AF. Mitochondrial DNA variation within and between two species of neotropical anopheline mosquitoes (Diptera: Culicidae). *J Hered* 1997, **88**(2)**:** 98-107.

45. Conn JE, Mitchell SE, Cockburn AF. Mitochondrial DNA analysis of the neotropical malaria vector *Anopheles nuneztovari. Genome* 1998, **41**(3)**:** 313-327.

46. Cook S, Diallo M, Sall AA, Cooper A, Holmes EC. Mitochondrial markers for molecular identification of *Aedes* mosquitoes (Diptera: Culicidae) involved in transmission of arboviral

disease in west Africa. *Journal of Medical Entomology* 2005, **42**(1)**:** 19-28.

47. Cook S, Ngo GL, Mcalister E, Harbach RE. *Bothaella manhi*, a new species of tribe Aedini (Diptera: Culicidae) from the Cuc Phuong National Park of Vietnam based on morphology and DNA sequence. *Zootaxa* 2010(2661)**:** 33-46.

48. Cooper RD, Edstein MD, Frances SP, Beebe NW. Malaria vectors of Timor-Leste. *Malaria journal* 2010, **9**.

49. Cooper RD, Waterson DGE, Bangs HJ, Beebe NW. Rediscovery of *Anopheles (Cellia) clowi* (Diptera: culicidae), a rarely recorded member of the *Anopheles punctulatus* group. *Journal of Medical Entomology* 2000, **37**(6)**:** 840-845.

50. Cywinska A, Hunter FF, Hebert PDN. Identifying Canadian mosquito species through DNA barcodes. *Medical and Veterinary Entomology* 2006, **20**(4)**:** 413-424.

51. de Almeida RW, Tovar FJ, Ferreira II, Leoncini O. Chymotrypsin genes in the malaria mosquitoes *Anopheles aquasalis* and *Anopheles darlingi*. *Insect biochemistry and molecular biology* 2003, **33**(3)**:** 307-315.

52. de Azevedo GM, Guimaraes-Marques GM, Bridi LC, Ohse KC, Vicentini R, Tadei W*, et al.* Phylogenetic analysis of the GST family in *Anopheles (Nyssorhynchus) darlingi*. *Acta tropica* 2014, **136:** 27-31.

53. Dehghan H, Sadraei J, Moosa-Kazemi SH, Baniani NA, Nowruzi F. The molecular and morphological variations of *Culex pipiens* complex (Diptera: Culicidae) in Iran. *Journal of vector borne diseases* 2013, **50**(2)**:** 111-120.

54. Deitz KC, Athrey G, Reddy MR, Overgaard HJ, Matias A, Jawara M*, et al.* Genetic isolation within the malaria mosquito *Anopheles melas*. *Molecular ecology* 2012, **21**(18)**:** 4498-4513.

55. Demari-Silva B, Vesgueiro FT, Sallum MAM, Marrelli MT. Taxonomic and Phylogenetic Relationships Between Species of the Genus Culex (Diptera: Culicidae) from Brazil Inferred from the Cytochrome c Oxidase I Mitochondrial Gene. *Journal of Medical Entomology* 2011, **48**(2)**:** 272-279.

56. Diao YP, Qi YM, Ma YJ, Xia A, Sharakhov I, Chen XG*, et al.* Next-Generation Sequencing Reveals Recent Horizontal Transfer of a DNA Transposon between Divergent Mosquitoes. *PLoS One* 2011, **6**(2).

57. Diaz-Nieto LM, Macia A, Parisi G, Farina JL, Vidal-Dominguez ME, Perotti MA*, et al.* Distribution of mosquitoes in the south east of Argentina and first report on the analysis based on 18S rDNA and COI sequences. *PLoS One* 2013, **8**(9).

58. Dinparast Djadid N, Sanati MH, Zare M, Hassanzehi A. rDNA-ITS2 identification of *Anopheles pulcherrimus* (Diptera: Culicidae): Genetic differences and phylogenetic relation with other Iranian vectors and its implications for malaria control. *Iranian biomedical journal* 2003, **7**(1)**:** 1-6.

59. Dixit J, Srivastava H, Sharma M, Das MK, Singh OP, Raghavendra K*, et al.* Phylogenetic inference of Indian malaria vectors from multilocus DNA sequences. *Infection Genetics and Evolution* 2010, **10**(6)**:** 755-763.

60. Djadid ND, Gholizadeh S, Aghajari M, Zehi AH, Raeisi A, Zakeri S. Genetic analysis of rDNA-ITS2 and RAPD loci in field populations of the malaria vector, *Anopheles stephensi* (Diptera: Culicidae): Implications for the control program in Iran. *Acta tropica* 2006, **97**(1)**:** 65-74.

61. Djadid ND, Gholizadeh S, Tafsiri E, Romi R, Gordeev M, Zakeri S. Molecular identification of Palearctic members of *Anopheles maculipennis* in northern Iran. *Malaria journal* 2007, **6**.

62. Djadid ND, Jazayeri H, Gholizadeh S, Rad SP, Zakeri S. First record of a new member of *Anopheles hyrcanus* group from Iran: molecular identification, diagnosis, phylogeny, status of kdr resistance and *Plasmodium* infection. *Journal of Medical Entomology* 2009, **46**(5)**:** 1084-1093.

63. Donnelly MJ, Corbel V, Weetman D, Wilding CS, Williamson MS, Black WC. Does kdr genotype predict insecticide-resistance phenotype in mosquitoes? *Trends Parasitol* 2009, **25**(5)**:** 213-219.

64. Doosti S, Vatandoost H, Oshaghi MA, Hosseini M, Sedaghat MM. Applying morphometric variation of Seta 2 (Antepalmate hair) among the larvae of the members of the *maculipennis* subgroup (Diptera: Culicidae) in Iran. *Iran J Arthropod-Bor* 2007, **1**(1)**:** 28-37.

65. dos Santos JMM, Maia JD, Tadei WP, Rodriguez GAD. Isoenzymatic variability among five *Anopheles* species belonging to the *Nyssorhynchus* and *Anopheles* subgenera of the Amazon Region, Brazil. *Memorias do Instituto Oswaldo Cruz* 2003, **98**(2)**:** 247-253.

66. Dusfour I, Michaux JR, Harbach RE, Manguin S. Speciation and phylogeography of the Southeast Asian *Anopheles sundaicus* complex. *Infection Genetics and Evolution* 2007, **7**(4)**:** 484-493.

67. Emerson KJ, Merz CR, Catchen JM, Hohenlohe PA, Cresko WA, Bradshaw WE*, et al.* Resolving postglacial phylogeography using high-throughput sequencing. *Proceedings of the National Academy of Sciences of the United States of America* 2010, **107**(37)**:** 16196-16200.

68. Endersby NM, White VL, Chan J, Hurst T, Rasic G, Miller A*, et al.* Evidence of cryptic genetic lineages within *Aedes notoscriptus* (Skuse). *Infection Genetics and Evolution* 2013, **18:** 191-201.

69. Engdahl C, Larsson P, Naslund J, Bravo M, Evander M, Lundstrom JO, *et al.* Identification of Swedish mosquitoes based on molecular barcoding of the COI gene and SNP analysis. *Molecular ecology resources* 2014, **14**(3)**:** 478-488.

70. Fallon AM, Li L. The c-terminal extension that characterizes mosquito (Diptera: Culicidae) ribosomal protein S6 is widespread among the Culicomorpha. *Journal of Medical Entomology* 2007, **44**(4)**:** 608-616.

71. Foley DH, Bryan JH, Yeates D, Saul A. Evolution and systematics of *Anopheles*: Insights from a molecular phylogeny of Australasian mosquitoes. *Mol Phylogenet Evol* 1998, **9**(2)**:** 262-275.

72. Foley DH, Meek SR, Bryan JH. The *Anopheles punctulatus* group of mosquitos in the Solomon-Islands and Vanuatu surveyed by allozyme electrophoresis. *Medical and Veterinary Entomology* 1994, **8**(4)**:** 340-350.

73. Foley DH, Paru R, Dagoro H, Bryan JH. Allozyme analysis reveals 6 Species within the *Anopheles punctulatus* complex of mosquitos in Papua-New-Guinea. *Medical and Veterinary Entomology* 1993, **7**(1)**:** 37-48.

74. Foley DH, Wilkerson RC, Cooper RD, Volovsek ME, Bryan JH. A molecular phylogeny of *Anopheles annulipes* (Diptera: Culicidae) sensu lato: The most species-rich anopheline complex. *Mol Phylogenet Evol* 2007, **43**(1)**:** 283-297.

75. Foster PG, Bergo ES, Bourke BP, Oliveira TMP, Nagaki SS, Sant'Ana DC, *et al.* Phylogenetic analysis and DNA-based species confirmation in *Anopheles (Nyssorhynchus*). *PLoS One* 2013, **8**(2).

76. Fritz GN, Bermudez H, Seawright JA. Genetic differentiation and diagnostic loci of *Anopheles nuneztovari*, an-trinkae, and an-rangeli (Diptera, Culicidae). *Journal of Medical Entomology* 1995, **32**(5)**:** 663-672.

77. Garcia BA, Caccone A, Mathiopoulos KD, Powell JR. Inversion monophyly in African anopheline malaria vectors. *Genetics* 1996, **143**(3)**:** 1313-1320.

78. Garros C, Harbach RE, Manguin S. Systematics and biogeographical implications of the phylogenetic relationships between members of the *funestus* and *minimus* Groups of Anopheles (Diptera: Culicidae). *Journal of Medical Entomology* 2005, **42**(1)**:** 7-18.

79. Garros C, Harbach RE, Manguin S. Morphological assessment and molecular phylogenetics of the *funestus* and *minimus* groups of *Anopheles (Cellia)*. *Journal of Medical Entomology* 2005, **42**(4)**:** 522-536.

80. Gholizadeh S, Djadid ND, Nouroozi B, Bekmohammadi M. Molecular phylogenetic analysis of

Anopheles and *Cellia* subgenus anophelines (Diptera: Culicidae) in temperate and tropical regions of Iran. *Acta tropica* 2013, **126**(1)**:** 63-74.

81. Gimnig JE. Genetic and morphological variation in three snow pool *Aedes* mosquito species of the subgenus *Ochlerotatus* (Diptera: Culicidae). *Journal of Medical Entomology* 2000, **37**(6)**:** 902-908.

82. Gimnig JE, Eldridge BF. Genetic and morphological characterization of the *Aedes (Ochlerotatus) dorsalis* (Diptera: Culicidae) group in North America. *Journal of Medical Entomology* 1999, **36**(6)**:** 685-694.

83. Gordeev M, Goriacheva I, Shaikevitch E, Ejov M. Intraspecific variability of the second internal transcribed spacer of the ribosomal DNA among five Palaearctic species of anopheline mosquitoes. *European Mosquito Bulletin* 2004, **17:** 14-19.

84. Green CA. Cladistic analysis of mosquito chromosome data (*Anopheles (Cellia)* Myzomyia). *J Hered* 1982, **73**(1)**:** 2-11.

85. Chu F, Qian G. A numerical phylogenetic analysis of the Chinese *Uranotaenia* mosquitoes (Dipera: Culicidae) [J]. *Acta Entomol Sinica* 1988, **3:** 010.

86. Gutierrez LA, Orrego LM, Gomez GF, Lopez A, Luckhart S, Conn JE*, et al.* A new mtDNA COI gene lineage closely related to *Anopheles janconnae* of the *albitarsis* complex in the Caribbean region of Colombia. *Memorias do Instituto Oswaldo Cruz* 2010, **105**(8)**:** 1019-1025.

87. Harbach RE. Review of the Internal Classification of the Genus *Anopheles* (Diptera, Culicidae) - the foundation for comparative systematics and phylogenetic research. *Bulletin of Entomological Research* 1994, **84**(3)**:** 331-342.

88. Harbach RE. The classification of genus *Anopheles* (Diptera: Culicidae): a working hypothesis of phylogenetic relationships. *Bulletin of Entomological Research* 2004, **94**(6)**:** 537-553.

89. Harbach RE. The Culicidae (Diptera): a review of taxonomy, classification and phylogeny. *Zootaxa* 2007(1668)**:** 591-638.

90. Harbach RE. Classification within the cosmopolitan genus *Culex* (Diptera: Culicidae): The foundation for molecular systematics and phylogenetic research. *Acta tropica* 2011, **120**(1-2)**:** 1-14.

91. Harbach RE, Howard TM. Corrections in the status and rank of names used to denote varietal forms of mosquitoes (Diptera: Culicidae). *Zootaxa* 2007(1542)**:** 35-48.

92. Harbach RE, Huong VD, Kitching JJ. Systematics of *Kimia*, a new genus of Sabethini (Diptera: Culicidae) in the Oriental Region. *P Entomol Soc Wash* 2007, **109**(1)**:** 102-120.

93. Harbach RE, Kitching IJ. Phylogeny and classification of the Culicidae (Diptera). *Syst Entomol* 1998, **23**(4)**:** 327-370.

94. Harbach RE, Kitching IJ. Reconsideration of anopheline mosquito phylogeny (Diptera: Culicidae: Anophelinae) based on morphological data. *Syst Biodivers* 2005, **3**(4)**:** 345-374.

95. Harbach RE, Kitching IJ, Culverwell CL, Dubois J, Linton YM. Phylogeny of mosquitoes of tribe Culicini (Diptera: Culicidae) based on morphological diversity. *Zool Scr* 2012, **41**(5)**:** 499-514.

96. Harbach RE, Kitching IJ, Culverwell CL, Howard TM, Linton YM. *Nyx pholeocola*, a new genus and *cavernicolous* species of tribe Aedini (Diptera: Culicidae) from southern Thailand based on morphological and molecular data. *Zootaxa* 2013, **3683**(2)**:** 159-177.

97. Harbach RE, Peyton E. Systematics of Onirion, a new genus of Sabethini (Diptera: Culicidae) from the Neotropical Region. *Bulletin of the Natural History Museum Entomology Series.* 2000, **69**(2)**:** 115-170.

98. Harrison BA, Ruiz-Lopez F, Falero GC, Savage HM, Pecor JE, Wilkerson RC. *Anopheles (Kerteszia) lepidotus* (Diptera: Culicidae), not the malaria vector we thought it was: Revised male and female morphology; larva, pupa, and male genitalia characters; and molecular verification. *Zootaxa* 2012(3218)**:** 1-17.

99. Hasan AU, Suguri S, Fujimoto C, Itaki RL, Harada M, Kawabata M*, et al.* Genetic diversity in two sibling species of the *Anopheles punctulatus* group of mosquitoes on Guadalcanal in the Solomon Islands. *BMC Evolutionary Biology* 2008, **8**.

100. Hemmerter S, Slapeta J, Beebe NW. Resolving genetic diversity in Australasian *Culex* mosquitoes: Incongruence between the mitochondrial cytochrome c oxidase I and nuclear acetylcholine esterase 2. *Mol Phylogenet Evol* 2009, **50**(2)**:** 317-325.

101. Hemmerter S, Slapeta J, van den Hurk AF, Cooper RD, Whelan PI, Russell RC*, et al.* A curious coincidence: mosquito biodiversity and the limits of the Japanese encephalitis virus in Australasia. *BMC Evolutionary Biology* 2007, **7**.

102. Hempolchom C, Otsuka Y, Baimai V, Thongsahuan S, Saeung A, Taai K*, et al.* Development of a multiplex PCR assay for the identification of eight species members of the Thai Hyrcanus Group (Diptera: Culicidae). *Appl Entomol Zool* 2013, **48**(4)**:** 469-476.

103. Henry-Halldin CN, Nadesakumaran K, Keven JB, Zimmerman AM, Siba P, Mueller I*, et al.* Multiplex assay for species identification and monitoring of insecticide resistance in *Anopheles punctulatus* group populations of Papua New Guinea. *American Journal of Tropical Medicine and Hygiene* 2012, **86**(1)**:** 140-151.

104. Hernandez VP, Higgins L, Fallon AM. Characterization and cDNA cloning of an immune-induced lysozyme from cultured *Aedes albopictus* mosquito cells. *Developmental and comparative immunology* 2003, **27**(1)**:** 11-20.

105. Herrera F, Urdaneta L, Rivero J, Zoghbi N, Ruiz J, Carrasquel G*, et al.* Population genetic structure of the dengue mosquito *Aedes aegypti* in Venezuela. *Memorias do Instituto Oswaldo Cruz* 2006, **101**(6)**:** 625-633.

106. Hesson JC, Lundstrom JO, Halvarsson P, Erixon P, Collado A. A sensitive and reliable restriction enzyme assay to distinguish between the mosquitoes *Culex torrentium* and *Culex pipiens*. *Medical and Veterinary Entomology* 2010, **24**(2)**:** 142-149.

107. Hilburn LR, Cooksey LM. Patterns of genetic variability in *Anopheles quadrimaculatis* (sensu stricto) (Diptera: Culicidae) populations in Eastern Arkansas. *Journal of Medical Entomology* 2004, **41**(1)**:** 40-46.

108. Hilburn LR, Rai KS. Electrophoretic similarities and mating compatibility among four species of the *Aedes (Stegomyia) scutellaris* complex (Diptera: Culicidae). *Journal of Medical Entomology* 1981, **18**(5)**:** 401-408.

109. Hittinger CT, Johnston M, Tossberg JT, Rokas A. Leveraging skewed transcript abundance by RNA-Seq to increase the genomic depth of the tree of life. *Proc Natl Acad Sci U S A* 2010, **107**(4)**:** 1476-1481.

110. Ho CM, Liu YM, Wei YH, Hu ST. Gene for cytochrome C oxidase subunit II in the mitochondrial DNA of *Culex quinquefasciatus* and *Aedes aegypti* (Diptera: Culicidae). *J Med Entomol* 1995, **32**(2)**:** 174-180.

111. Hu X, Wang W, Zhang D, Jiao J, Tan W, Sun Y*, et al.* Cloning and characterization of 40S ribosomal protein S4 gene from *Culex pipiens pallens*. *Comparative biochemistry and physiology Part B, Biochemistry & molecular biology* 2007, **146**(2)**:** 265-270.

112. Huang S, Sun D, Brattsten LB. Novel cytochrome P450s, CYP6BB1 and CYP6P10, from the salt marsh mosquito *Aedes sollicitans* (Walker) (Diptera: Culicidae). *Arch Insect Biochem Physiol* 2008, **67**(3)**:** 139-154.

113. Huang YM, Mathis WN, Wilkerson RC. Coetzeemyia, a new subgenus of *Aedes*, and a redescription of the holotype female of *Aedes (Coetzeemyia) fryeri* (Theobald) (Diptera: Culicidae). *Zootaxa* 2010(2638)**:** 1-24.

114. Huang Z-h, Wang J-f. Molecular evolution of the mitochondrial cytochrome C oxidase II gene of three mosquitoes. *JOURNAL-ZHEJIANG UNIVERSITY-SCIENCES EDITION-* 2003, **30**(4)**:** 457-460.

115. Humeres SG, Gardenal CN, Almiron W, Sereno R, Sabattini MS. *Culex* species (Diptera, Culicidae) from Central Argentina - Identification by electrophoretic zymograms and genetic-relationships. *Journal of Medical Entomology* 1990, **27**(5)**:** 784-788.

116. Hwang UW. Revisited ITS2 phylogeny of *Anopheles (Anopheles) hyrcanus* group mosquitoes: reexamination of unidentified and misidentified ITS2 sequences. *Parasitology research* 2007, **101**(4)**:** 885-894.

117. Imwong M, Sharpe RG, Kittayapong P, Baimai V. Distribution of the transposable element mariner in anopheline mosquitoes. *Heredity* 2000, **85**(3)**:** 271-276.

118. Janice J, Pande A, Weiner J, Lin CF, Makalowski W. U12-type Spliceosomal Introns of Insecta. *Int J Biol Sci* 2012, **8**(3)**:** 344-352.

119. Jimenez-Guri E, Huerta-Cepas J, Cozzuto L, Wotton KR, Kang H, Himmelbauer H*, et al.* Comparative transcriptomics of early dipteran development. *BMC genomics* 2013, **14**.

120. Jin L, Luo J, Guo Y, Xie L, Fu Y. Phylogenetic analysis of *Culex fuscanus*, *Culex bitaeniorhynchus* and *Culex pipiens quinquefasciatus* (Diptera: Culicidae), inferred from the internal transcribed spacer 2 of ribosomal DNA. *Acta Parasitology et Medica Entomologica Sinica* 2007, **14**(1)**:** 29.

121. Judd DD. Review of the systematics and phylogenetic relationships of the Sabethini (Diptera: Culicidae). *Syst Entomol* 1996, **21**(2)**:** 129-150.

122. Judd DD. Review of a bromeliad-ovipositing lineage in *Wyeomyia* and the resurrection of *Hystatomyia* (Diptera: Culicidae). *Ann Entomol Soc Am* 1998, **91**(5)**:** 572-589.

123. Judd DD. Exploring component stability using life-stage concordance in sabethine mosquitoes (Diptera: Culicidae). *Cladistics-the International Journal of the Willi Hennig Society* 1998, **14**(1)**:** 63-93.

124. Kamali M, Marek PE, Peery A, Antonio-Nkondjio C, Ndo C, Tu ZJ*, et al.* Multigene phylogenetics reveals temporal diversification of major African malaria vectors. *PLoS One* 2014, **9**(4).

125. Kambhampati S, Rai KS. Variation in mitochondrial-DNA of *Aede*s species (Diptera, Culicidae). *Evolution; international journal of organic evolution* 1991, **45**(1)**:** 120-129.

126. Kamita SG, Samra AI, Liu JY, Cornel AJ, Hammock BD. Juvenile Hormone (JH) esterase of the mosquito *Culex quinquefasciatus* is not a target of the JH analog insecticide Methoprene. *PLoS One* 2011, **6**(12).

127. Kampen H. The ITS2 ribosomal DNA of *Anopheles beklemishevi* and further remarks on the

phylogenetic relationships within the *Anopheles maculipennis* group of species (Diptera: Culicidae). *Parasitology research* 2005, **97**(2)**:** 118-128.

128. Kanojia PC, Paingankar MS, Patil AA, Gokhale MD, Deobagkar DN. Morphometric and allozyme variation in *Culex tritaeniorhynchus* mosquito populations from India. *Journal of insect science* 2010, **10**.

129. Khrabrova NV, andreeva Iu V, Sibataev AK, Stegnii VN. [Comparison of nucleotide sequences of ribosomal DNA of the mosquito genera *Aedes* and *Ochlerotatus* (Diptera: Culicidae: Aedini)]. *Parazitologiia* 2012, **46**(1)**:** 3-10.

130. Koekemoer LL, Misiani EA, Hunt RH, Kent RJ, Norris DE, Coetzee M. Cryptic species within *Anopheles longipalpis* from southern Africa and phylogenetic comparison with members of the *An. funestus* group (vol 99, pg 41, 2009). *Bulletin of Entomological Research* 2009, **99**(3)**:** 323-323.

131. Kohli R, Chaudhry S, Bedi G. Genomic analysis of some *Culex* mosquitoes (Culicidae: Diptera). *Electronic Journal of Biology* 2011, **7**(1)**:** 6-10.

132. Krzywinski J, Besansky NJ. Molecular systematics of *Anopheles*: from subgenera to subpopulations. *Annual Review of Entomology* 2003, **48:** 111-139.

133. Krzywinski J, Li C, Morris M, Conn JE, Lima JB, Povoa MM*, et al.* Analysis of the evolutionary forces shaping mitochondrial genomes of a Neotropical malaria vector complex. *Mol Phylogenet Evol* 2011, **58**(3)**:** 469-477.

134. Krzywinski J, Sangare D, Besansky NJ. Satellite DNA from the Y chromosome of the malaria vector *Anopheles gambiae*. *Genetics* 2005, **169**(1)**:** 185-196.

135. Krzywinski J, Wilkerson RC, Besansky NJ. Toward understanding Anophelinae (Diptera, Culicidae) phylogeny: Insights from nuclear single-copy genes and the weight of evidence. *Systematic Biology* 2001, **50**(4)**:** 540-556.

136. Krzywinski J, Wilkerson RC, Besansky NJ. Evolution of mitochondrial and ribosomal gene sequences in anophelinae (Diptera: Culicidae): implications for phylogeny reconstruction. *Mol Phylogenet Evol* 2001, **18**(3)**:** 479-487.

137. Kumar A, Black WC, Rai KS. An estimate of phylogenetic relationships among culicine mosquitoes using a restriction map of the rDNA cistron. *Insect molecular biology* 1998, **7**(4)**:** 367-373.

138. Kumar NP, Krishnamoorthy N, Sahu SS, Rajavel AR, Sabesan S, Jambulingam P. DNA Barcodes indicate members of the *Anopheles fluviatilis* (Diptera: Culicidae) species complex to be conspecific in India. *Molecular ecology resources* 2013, **13**(3)**:** 354-361.

139. Kumar NP, Rajavel AR, Natarajan R, Jambulingam P. DNA barcodes can distinguish species of Indian mosquitoes (Diptera: Culicidae). *Journal of Medical Entomology* 2007, **44**(1)**:** 1-7.

140. Laboudi M, Faraj C, Sadak A, Harrat Z, Boubidi SC, Harbach RE*, et al.* DNA barcodes confirm the presence of a single member of the *Anopheles maculipennis* group in Morocco and Algeria: *An. sicaulti* is conspecific with *An. labranchiae*. *Acta tropica* 2011, **118**(1)**:** 6-13.

141. Lan CJ, Shen Y, Liang LU, Guang-Yuan MA. Molecular evolution analysis of CO Ⅰ gene in five species of Culex. *Chinese Journal of Vector Biology & Control* 2013.

142. Laurito M, Almiron WR. Phylogenetic relationships of *Culex (Culex*) species (Diptera, Culicidae) from Argentina based on morphological characters. *Zootaxa* 2013, **3652**(1)**:** 117-155.

143. Laurito M, de Oliveira TMP, Almiron WR, Sallum MAM. COI barcode versus morphological identification of *Culex (Culex)* (Diptera: Culicidae) species: a case study using samples from Argentina and Brazil. *Memorias do Instituto Oswaldo Cruz* 2013, **108:** 110-U152.

144. Le Goff G, Brengues C, Robert V. *Stegomyia* mosquitoes in Mayotte, taxonomic study and description of *Stegomyia* pia n. sp. *Parasite* 2013, **20**.

145. Lehr MA, Kilpatrick CW, Wilkerson RC, Conn JE. Cryptic species in the *Anopheles (Nyssorhynchus) albitarsis* (Diptera: Culicidae) complex: Incongruence between random amplified polymorphic DNA-polymerase chain reaction identification and analysis of mitochondrial DNA COI gene sequences. *Ann Entomol Soc Am* 2005, **98**(6)**:** 908-917.

146. Li L, Fallon AM. Recovery of cDNAs encoding ribosomal proteins S9 and L26 from *Aedes albopictus* mosquito cells and identification of their homologs in the malaria vector, *Anopheles gambiae*. *Arch Insect Biochem* 2005, **60**(1)**:** 44-53.

147. Lima RS, Scarpassa VM. Evidence of two lineages of the dengue vector *Aedes aegypti* in the Brazilian Amazon, based on mitochondrial DNA ND4 gene sequences. *Genet Mol Biol* 2009, **32**(2)**:** 414-422.

148. Linton YM, Pecor JE, Porter CH, Mitchell LB, Garzon-Moreno A, Foley DH*, et al.* Mosquitoes of eastern Amazonian Ecuador: biodiversity, bionomics and barcodes. *Memorias do Instituto Oswaldo Cruz* 2013, **108:** 100-U132.

149. Liu Y, Chen H. Cladistic analysis of nine subgenera of *Culex* from China (Diptera: Culicidae). *Acta Parasitologica et Medica Entomologica Sinica* 2000, **7**(4)**:** 218-224.

150. Loaiza JR, Scott ME, Bermingham E, Sanjur OI, Rovira JR, Dutari LC*, et al.* Novel genetic diversity within *Anopheles punctimacula* s.l.: Phylogenetic discrepancy between the Barcode cytochrome c oxidase I (COI) gene and the rDNA second internal transcribed spacer (ITS2).

Acta tropica 2013, **128**(1)**:** 61-69.

151. Logue K, Chan ER, Phipps T, Small ST, Reimer L, Henry-Halldin C*, et al.* Mitochondrial genome sequences reveal deep divergences among *Anopheles punctulatus* sibling species in Papua New Guinea. *Malaria journal* 2013, **12**.

152. Lombardo F, Ronca R, Rizzo C, Mestres-Simon M, Lanfrancotti A, Curra C*, et al.* The *Anopheles gambiae* salivary protein gSG6: An anopheline-specific protein with a blood-feeding role. *Insect biochemistry and molecular biology* 2009, **39**(7)**:** 457-466.

153. Lounibos LP, Wilkerson RC, Conn JE, Hribar LJ, Fritz GN, Danoff-Burg JA. Morphological, molecular, and chromosomal discrimination of cryptic *Anopheles (Nyssorhynchus)* (Diptera: Culicidae) from South America. *Journal of Medical Entomology* 1998, **35**(5)**:** 830-838.

154. Low VL, Lim PE, Chen CD, Lim YAL, Tan TK, Norma-Rashid Y*, et al.* Mitochondrial DNA analyses reveal low genetic diversity in *Culex quinquefasciatus* from residential areas in Malaysia. *Medical and Veterinary Entomology* 2014, **28**(2)**:** 157-168.

155. Ma L, Gerenday A, Coley KM, Fallon AM. Co-immunoprecipitation of putative proteins that interact with mosquito proliferating cell nuclear antigen. *Insect molecular biology* 2006, **15**(2)**:** 197-205.

156. Ma YJ, Xu JN. The *hyrcanus* group of *Anopheles (Anopheles)* in China (Diptera: Culicidae): Species discrimination and phylogenetic relationships inferred by ribosomal DNA internal transcribed spacer 2 sequences. *Journal of Medical Entomology* 2005, **42**(4)**:** 610-619.

157. Ma YJ, Xu JN, Li SZ. Molecular identification and phylogeny of the *maculatus* group of *Anopheles* mosquitoes (Diptera: Culicidae) based on nuclear and mitochondrial DNA sequences. *American Journal of Tropical Medicine and Hygiene* 2006, **75**(5)**:** 67-67.

158. Manguin S, Wilkerson RC, Conn JE, Rubio-Palis Y, Danoff-Burg JA, Roberts DR. Population structure of the primary malaria vector in South America, *Anopheles darlingi*, using isozyme, random amplified polymorphic DNA, internal transcribed spacer 2, and morphologic markers. *American Journal of Tropical Medicine and Hygiene* 1999, **60**(3)**:** 364-376.

159. Manh ND, Hinh TD, Harbach RE, Elphick J, Linton YM. A new species of the *hyrcanus* group of *Anopheles*, subgenus *Anopheles*, a secondary vector of malaria in coastal areas of southern Vietnam. *Journal of the American Mosquito Control Association* 2000, **16**(3)**:** 189-198.

160. Marinucci M, Romi R, Mancini P, Di Luca M, Severini C. Phylogenetic relationships of seven palearctic members of the *maculipennis* complex inferred from ITS2 sequence analysis. *Insect molecular biology* 1999, **8**(4)**:** 469-480.

161. Marrelli MT, Floeter-Winter LM, Malafronte RS, Tadei WP, Lourenco-de-Oliveira R,

Flores-Mendoza C*, et al.* Amazonian malaria vector anopheline relationships interpreted from ITS2 rDNA sequences. *Medical and Veterinary Entomology* 2005, **19**(2)**:** 208-218.

162. Mathiopoulos KD, Powell JD, Mccutchan TF. An anchored restriction-mapping approach applied to the genetic-analysis of the *Anopheles gambiae* malaria vector complex-1. *Mol Biol Evol* 1995, **12**(1)**:** 103-112.

163. Mayoral JG, Leonard KT, Nouzova M, Noriega FG, Defelipe LA, Turjanski AG. Functional analysis of a mosquito short-chain dehydrogenase cluster. *Arch Insect Biochem* 2013, **82**(2)**:** 96-115.

164. Mayoral JG, Nouzova M, Brockhoff A, Goodwin M, Hernandez-Martinez S, Richter D*, et al.* Allatostatin-C receptors in mosquitoes. *Peptides* 2010, **31**(3)**:** 442-450.

165. Mehravaran A, Oshaghi MA, Vatandoost H, Abai MR, Ebrahimzadeh A, Roodi AM*, et al.* First report on *Anopheles fluviatilis* U in southeastern Iran. *Acta tropica* 2011, **117**(2)**:** 76-81.

166. Merritt TJS, Young CR, Vogt RG, Wilkerson RC, Quattro JM. Intron retention identifies a malaria vector within the *Anopheles (Nyssorhynchus) albitaris* complex (Diptera: Culicidae). *Mol Phylogenet Evol* 2005, **35**(3)**:** 719-724.

167. Miller BR, Crabtree MB, Savage HM. Phylogeny of fourteen *Culex* mosquito species, including the *Culex pipiens* complex, inferred from the internal transcribed spacers of ribosomal DNA. *Insect molecular biology* 1996, **5**(2)**:** 93-107.

168. Miller BR, Crabtree MB, Savage HM. Phylogenetic relationships of the Culicomorpha inferred from 18S and 5.8S ribosomal DNA sequences (Diptera: Nematocera). *Insect molecular biology* 1997, **6**(2)**:** 105-114.

169. Mitchell A, Sperling FAH, Hickey DA. Higher-level phylogeny of mosquitoes (Diptera: Culicidae): mtDNA data support a derived placement for *Toxorhynchites*. *Insect Syst Evol* 2002, **33**(2)**:** 163-174.

170. Miura S, Nozawa M, Nei M. Evolutionary Changes of the target sites of two microRNAs encoded in the Hox gene cluster of *Drosophila* and other insect species. *Genome biology and evolution* 2011, **3:** 129-139.

171. Mohanty A, Swain S, Kar SK, Hazra RK. Analysis of the phylogenetic relationship of *Anopheles* species, subgenus *Cellia* (Diptera: Culicidae) and using it to define the relationship of morphologically similar species. *Infection Genetics and Evolution* 2009, **9**(6)**:** 1204-1224.

172. Molina-Cruz A, De Merida AMP, Mills K, Rodriguez F, Schoua C, Yurrita MM*, et al.* Gene flow among *Anopheles albimanus* populations in Central America, South America, and the Caribbean assessed by microsatellites and mitochondrial DNA. *American Journal of Tropical*

Medicine and Hygiene 2004, **71**(3)**:** 350-359.

173. Moore M, Sylla M, Goss L, Burugu MW, Sang R, Kamau LW*, et al.* Dual African origins of global *Aedes aegypti* s.l. populations revealed by mitochondrial DNA. *PLoS neglected tropical diseases* 2013, **7**(4).

174. Moreno M, Bickersmith S, Harlow W, Hildebrandt J, McKeon SN, Silva-do-Nascimento TF*, et al.* Phylogeography of the neotropical *Anopheles triannulatus* complex (Diptera: Culicidae) supports deep structure and complex patterns. *Parasites & vectors* 2013, **6**.

175. Morgan K, O'Loughlin SM, Fong MY, Linton YM, Somboon P, Min S*, et al.* Molecular phylogenetics and biogeography of the *Neocellia* series of *Anopheles* mosquitoes in the Oriental Region. *Mol Phylogenet Evol* 2009, **52**(3)**:** 588-601.

176. Motta MA, Lourenco-de-Oliveira R, Monteiro FA, Barros LR. Preliminary evaluation of the genetic relatedness of three species of the subgenus *Dendromyia* Theobald and other species of the genus *Wyeomyia* Theobald (Diptera: Culicidae). *Memorias do Instituto Oswaldo Cruz* 1998, **93**(2)**:** 189-194.

177. Motta MA, Lourenco-de-Oliveira R, Sallum MAM. Phylogeny of genus *Wyeomyia* (Diptera: Culicidae) inferred from morphological and allozyme data. *Can Entomol* 2007, **139**(5)**:** 591-627.

178. Munstermann LE. Mosquito systematics: Current status, new trends, associated complications. *Journal of Vector Ecology* 1995, **20**(2)**:** 129-138.

179. Munstermann LE, Conn JE. Systematics of mosquito disease vectors (Diptera, Culicidae): Impact of molecular biology and cladistic analysis. *Annual Review of Entomology* 1997, **42:** 351-369.

180. Namin HH, Iranpour M, Sharanowski BJ. Phylogenetics and molecular identification of the *Ochlerotatus communis* complex (Diptera: Culicidae) using DNA barcoding and polymerase chain reaction-restriction fragment length polymorphism. *Can Entomol* 2014, **146**(1)**:** 26-35.

181. Navarro JC, Hernandez C, Rangel-Diaz G, Rangel Y, Guerrero E, Arrivillaga J. Invalidation of putative autapomorphic characters using molecular phylogenetic analyses in *Anopheles* subgenus *Nyssorhynchus*. 2009, **49**(2)**:** 223-239.

182. Navarro JC, Liria J. Phylogenetic relationships among eighteen neotropical Culicini species. *Journal of the American Mosquito Control Association* 2000, **16**(2)**:** 75-85.

183. Navarro JC, Weaver SC. Molecular phylogeny of the *vomerifer* and *pedroi* groups in the *spissipes* section of the subgenus Culex (Melanoconion). *Journal of Medical Entomology* 2004, **41**(4)**:** 575-581.

184. Ndo C, Simard F, Kengne P, Awono-Ambene P, Morlais I, Sharakhov I*, et al.* Cryptic genetic diversity within the *Anopheles nili* group of malaria vectors in the equatorial forest area of Cameroon (Central Africa). *PLoS One* 2013, **8**(3).

185. Ngo CT, Harbach RE, Garros C, Parzy D, Le HQ, Manguin S. Taxonomic assessment of *Anopheles crawfordi* and *An. dangi* of the *hyrcanus* group of subgenus *Anopheles* in Vietnam. *Acta tropica* 2013, **128**(3)**:** 623-629.

186. Papavero N, Guimaraes JH. The taxonomy of Brazilian insects vectors of transmissible diseases (1900-2000) - Then and now. *Memorias do Instituto Oswaldo Cruz* 2000, **95:** 109-118.

187. Pape T. Cladistic analyses of mosquito chromosome data in *Anopheles* subgenus *Cellia* (Diptera: Culicidae). *Mosquito Systematics* 1992, **24**.

188. Paramasivan R, Dhananjeyan KJ, Pandian RS. A preliminary report on DNA barcoding and phylogenetic relationships of certain public health important mosquito species recorded in rural areas of south India. *Journal of vector borne diseases* 2013, **50**(2)**:** 144-146.

189. Paredes-Esquivel C, Donnelly MJ, Harbach RE, Townson H. A molecular phylogeny of mosquitoes in the *Anopheles barbirostris* subgroup reveals cryptic species: Implications for identification of disease vectors. *Mol Phylogenet Evol* 2009, **50**(1)**:** 141-151.

190. Paredes-Esquivel C, Harbach RE, Townson H. Molecular taxonomy of members of the *Anopheles hyrcanus* group from Thailand and Indonesia. *Medical and Veterinary Entomology* 2011, **25**(3)**:** 348-352.

191. Pashley DP, Rai KS, Pashley DN. Patterns of allozyme relationships compared with morphology, hybridization, and geologic history in allopatric island-dwelling mosquitos. *Evolution; international journal of organic evolution* 1985, **39**(5)**:** 985-997.

192. Paskewitz SM, Wesson DM, Collins FH. The internal transcribed spacers of ribosomal DNA in five members of the *Anopheles gambiae* species complex. *Insect molecular biology* 1993, **2**(4)**:** 247-257.

193. Patsoula E, Samanidou-Voyadjoglou A, Spanakos G, Kremastinou J, Nasioulas G, Vakalis NC. Molecular and morphological characterization of *Aedes albopictus* in northwestern Greece and differentiation from *Aedes cretinus* and *Aedes aegypti*. *J Med Entomol* 2006, **43**(1)**:** 40-54.

194. Pawlowski J, Szadziewski R, Kmieciak D, Fahrni J, Bittar G. Phylogeny of the infraorder Culicomorpha (Diptera: Nematocera) based on 28S RNA gene sequences. *Syst Entomol* 1996, **21**(2)**:** 167-178.

195. Pfeiler E, Flores-Lopez CA, Mada-Velez JG, Escalante-Verdugo J, Markow TA. Genetic diversity and population genetics of mosquitoes (Diptera: Culicidae: *Culex* spp.) from the

Sonoran Desert of North America. *Sci World J* 2013.

196. Phillips A, Sabatini A, Milligan PJM, Boccolini D, Broomfield G, Molyneux DH. The *Anopheles maculipennis* complex (Diptera, Culicidae) - comparison of the cuticular hydrocarbon profiles determined in adults of 5 Palearctic species. *Bulletin of Entomological Research* 1990, **80**(4)**:** 459-464.

197. Poncon N, Toty C, Kengne P, Alten B, Fontenille D. Molecular evidence for similarity between *Anopheles hyrcanus* (Diptera: Culicidae) and *Anopheles pseudopictus* (Diptera: Culicidae), sympatric potential vectors of malaria in France. *Journal of Medical Entomology* 2008, **45**(3)**:** 576-580.

198. Porter CH, Collins FH. Phylogeny of nearctic members of the *Anopheles maculipennis* species group derived from the D2 variable region of 28S ribosomal RNA. *Mol Phylogenet Evol* 1996, **6**(2)**:** 178-188.

199. Powell JR, Petrarca V, della Torre A, Caccone A, Coluzzi M. Population structure, speciation, and introgression in the *Anopheles gambiae* complex. *Parassitologia, Vol 41, Nos 1-3, September* 1999**:** 101-113.

200. Puslednik L, Russell RC, Ballard JWO. Phylogeography of the medically important mosquito *Aedes (Ochlerotatus) vigilax* (Diptera: Culicidae) in Australasia. *J Biogeogr* 2012, **39**(7)**:** 1333-1346.

201. Quintero L, Navarro JC. Intraspecific phylogeny and genetic variability of *Culex quinquefasciatus* Say (Diptera: Culicidae) with mitochondrial genes ND5 and COI. *B Malariol Salud Amb* 2012, **52**(1)**:** 45-65.

202. Rashmi K, Sharma M, Chaudhry S. DNA fingerprinting and phylogenetics of five species of genus *Culex* using ITS2 sequence (Diptera: Culicidae). *Caryologia* 2011, **64**(1)**:** 3-9.

203. Reidenbach KR, Cook S, Bertone MA, Harbach RE, Wiegmann BM, Besansky NJ. Phylogenetic analysis and temporal diversification of mosquitoes (Diptera: Culicidae) based on nuclear genes and morphology. *BMC Evolutionary Biology* 2009, **9**.

204. Reinert JF, Harbach RE, Kitching IJ. Phylogeny and classification of Aedini (Diptera: Culicidae), based on morphological characters of all life stages. *Zoological Journal of the Linnean Society* 2004, **142**(3)**:** 289-368.

205. Reinert JF, Harbach RE, Kitching IJ. Phylogeny and classification of *Finlaya* and allied taxa (Diptera: Culicidae: Aedini) based on morphological data from all life stages. *Zoological Journal of the Linnean Society* 2006, **148**(1)**:** 1-101.

206. Reinert JF, Harbach RE, Kitching IJ. Phylogeny and classification of *Ochlerotatus* and allied

taxa (Diptera: Culicidae: Aedini) based on morphological data from all life stages. *Zoological Journal of the Linnean Society* 2008, **153**(1)**:** 29-114.

207. Reinert JF, Harbach RE, Kitching IJ. Phylogeny and classification of tribe Aedini (Diptera: Culicidae). *Zoological Journal of the Linnean Society* 2009, **157**(4)**:** 700-794.

208. Rey D, Despres L, Schaffner F, Meyran JC. Mapping of resistance to vegetable polyphenols among *Aedes* taxa (Diptera, Culicidae) on a molecular phylogeny. *Mol Phylogenet Evol* 2001, **19**(2)**:** 317-325.

209. Rossi GC, Harbach RE. *Phytotelmatomyia*, a new neotropical subgenus of *Culex* (Diptera: Culicidae). *Zootaxa* 2008(1879)**:** 1-17.

210. Rubio-Palis Y. Caracterización morfométrica de poblaciones del vector de malaria *Anopheles (Nyssorhynchus) darlingi* Root (Diptera: Culicidae) en Venezuela. *Bol Entomol Venez* 1998, **13**(2)**:** 141-172.

211. Rueanghiran C, Apiwathnasorn C, Sangthong P, Samung Y, Ruangsittichai J. Utility of a set of conserved mitochondrial cytochrome oxidase subunit I gene primers for *Mansonia annulata* identification. *Se Asian J Trop Med* 2011, **42**(6)**:** 1381-1387.

212. Ruiz-Garcia M, Bello F, Ramirez D, Alvarez D. Genetic structure of the genera *Psorophora* (Diptera: Culicidae) in Columbian and North American populations using isoenzymes and ITS2 sequences. *Russ J Genet+* 2006, **42**(7)**:** 752-765.

213. Ruiz-Lopez F, Wilkerson RC, Conn JE, McKeon SN, Levin DM, Quinones ML*, et al.* DNA barcoding reveals both known and novel taxa in the *Albitarsis* group (*Anopheles: Nyssorhynchus*) of Neotropical malaria vectors. *Parasites & vectors* 2012, **5**.

214. Ruiz-Lopez F, Wilkerson RC, Ponsonby DJ, Herrera M, Sallum MAM, Velez ID*, et al.* Systematics of the o*swaldoi* complex (*Anopheles, Nyssorhynchus*) in South America. *Parasites & vectors* 2013, **6**.

215. Saeung A, Baimai V, Thongsahuan S, Min GS, Park MH, Otsuka Y*, et al.* Geographic distribution and genetic compatibility among six karyotypic forms of *Anopheles peditaeniatus* (Diptera: Culicidae) in Thailand. *Tropical biomedicine* 2012, **29**(4)**:** 613-625.

216. Saeung A, Otsuka Y, Baimai V, Somboon P, Pitasawat B, Tuetun B*, et al.* Cytogenetic and molecular evidence for two species in the *Anopheles barbirostris* complex (Diptera: Culicidae) in Thailand. *Parasitology research* 2007, **101**(5)**:** 1337-1344.

217. Sallum MAM, Bergo ES, Flores DC, Forattini OP. Systematic studies on *Anopheles galvaoi* Causey, Deane & Deane from the subgenus *Nysssorhynchus* Blanchard (Diptera: Culicidae). *Memorias do Instituto Oswaldo Cruz* 2002, **97**(8)**:** 1177-1189.

218. Sallum MAM, Foster PG, dos Santos CLS, Flores DC, Motoki MT, Bergo ES. Resurrection of two species from synonymy of *Anopheles (Nyssorhynchus) strodei* Root, and characterization of a distinct morphological form from the s*trodei* complex (Diptera: Culicidae). *Journal of Medical Entomology* 2010, **47**(4)**:** 504-526.

219. Sallum MAM, Foster PG, Li C, Sithiprasasna R, Wilkerson RC. Phylogeny of the *leucosphyrus* group of *Anopheles (Cellia)* (Diptera: Culicidae) based on mitochondrial gene sequences. *Ann Entomol Soc Am* 2007, **100**(1)**:** 27-35.

220. Sallum MAM, Schultz TR, Foster PG, Aronstein K, Wirtz RA, Wilkerson RC. Phylogeny of Anophelinae (Diptera: Culicidae) based on nuclear ribosomal and mitochondrial DNA sequences. *Syst Entomol* 2002, **27**(3)**:** 361-382.

221. Sallum MAM, Schultz TR, Wilkerson RC. Phylogeny of Anophelinae (Diptera Culicidae) based on morphological characters. *Ann Entomol Soc Am* 2000, **93**(4)**:** 745-775.

222. Salvemini M, Mauro U, Lombardo F, Milano A, Zazzaro V, Arca B*, et al.* Genomic organization and splicing evolution of the doublesex gene, a *Drosophila* regulator of sexual differentiation, in the dengue and yellow fever mosquito *Aedes aegypti*. *BMC Evolutionary Biology* 2011, **11**.

223. Sathe T, Jagtap M. Taxonomy and molecular phylogeny of new species of the genus *Anopheles (Cellia)* Theobald (Culicidae: Diptera) from India. *Journal of Entomological Research* 2012, **36**(1)**:** 71-75.

224. Savage HM. Classification of mosquitoes in tribe aedini (Diptera: culicidae): *Paraphylyphobia*, and classification versus cladistic analysis. *Journal of Medical Entomology* 2005, **42**(6)**:** 923-927.

225. Savage HM, Strickman D. The genus and subgenus categories within culicidae and placement of *Ochlerotatus* as a subgenus of *Aedes*. *Journal of the American Mosquito Control Association* 2004, **20**(2)**:** 208-214.

226. Sawabe K, Takagi M, Tsuda Y, Tuno N. Molecular variation and phylogeny of the *Anopheles minimus* complex (Diptera: Culicidae) inhabiting Southeast Asian countries, based on ribosomal DNA internal transcribed spacers, ITS1 and 2, and the 28S D3 sequences. *The Southeast Asian journal of tropical medicine and public health* 2003, **34**(4)**:** 771-780.

227. Scarpassa VM, Cardoza TB, Cardoso Junior RP. Population genetics and phylogeography of *Aedes aegypti* (Diptera: Culicidae) from Brazil. *The American journal of tropical medicine and hygiene* 2008, **78**(6)**:** 895-903.

228. Scarpassa VM, Conn JE. Mitochondrial DNA Detects a complex evolutionary history with Pleistocene Epoch divergence for the Neotropical malaria vector *Anopheles nuneztovari* sensu

lato. *American Journal of Tropical Medicine and Hygiene* 2011, **85**(5)**:** 857-867.

229. Schultz JH, Meier PG, Newson HD. Evolutionary relationships among the salt marsh *Aedes* (Diptera: Culicidae). *Mosquito Systematics* 1986, **18**(2)**:** 145-170.

230. Shaikevich EV, Zagoskin MV, Mukha DV. Comparative characteristics of the intergenic spacer of the ribosomal RNA gene cluster in mosquitoes of the genus *Culex* (Diptera: Culicidae). *Mol Biol+* 2013, **47**(3)**:** 364-372.

231. Sharakhova MV, Antonio-Nkondjio C, Xia A, Ndo C, Awono-Ambene P, Simard F*, et al.* Cytogenetic map for *Anopheles nili*: Application for population genetics and comparative physical mapping. *Infection Genetics and Evolution* 2011, **11**(4)**:** 746-754.

232. Sharpe RG, Harbach RE, Butlin RK. Molecular variation and phylogeny of members of the *minimus* group of *Anopheles* subgenus *Cellia* (Diptera: Culicidae). *Syst Entomol* 2000, **25**(2)**:** 263-272.

233. SHENYuan, LANCe-Jie, MAGuang-Yuan, WUJia-Lin. CO I Gene molecular evolution of four species of mosquitoes. *ACTA PARASITOLOGICA ET MEDICA ENTOMOLOGICA SINICA* 2012, **19**(3)**:** 165-168.

234. Shepard JJ, andreadis TG, Vossbrinck CR. Molecular Phylogeny and evolutionary relationships among mosquitoes (Diptera: Culicidae) from the northeastern United States based on small subunit ribosomal DNA (18S rDNA) sequences. *Journal of Medical Entomology* 2006, **43**(3)**:** 443-454.

235. Shi zhu LI, Ya jun MA, Zheng ZM. Sequence analysis of mtDNA-COⅡ gene and molecular phylogeny in *Anopheles maculatus* complex from China (Diptera: Culicidae). *Journal of Shaanxi Normal University* 2003.

236. Shouche YS, Patole MS. Sequence analysis of mitochondrial 16S ribosomal RNA gene fragment from seven mosquito species. *J Bioscience* 2000, **25**(4)**:** 361-366.

237. Sieglaff DH, Dunn WA, Xie XHS, Megy K, Marinotti O, James AA. Comparative genomics allows the discovery of cis-regulatory elements in mosquitoes. *Proceedings of the National Academy of Sciences of the United States of America* 2009, **106**(9)**:** 3053-3058.

238. Silva-Do-Nascimento TF, Wilkerson RC, Lourenco-De-Oliveira R, Monteiro FA. Molecular confirmation of the specific status of *Anopheles halophylus* (Diptera: Culicidae) and evidence of a new cryptic species within *An. triannulatus* in central Brazil. *Journal of Medical Entomology* 2006, **43**(3)**:** 455-459.

239. Simon C. Application of numerical techniques to the systematics of *Toxorhynchites* (Diptera: Culicidae). 1983.

240. Simon C, Steffan W, Moss W, Evenhuis N. The genus *Toxorhynchites* (Diptera: Culicidae); numerical phylogenetic analysis of *Toxorhynchites splendens* and allies with phenetic comparisons. *Mosquito Systematics (USA)* 1982.

241. Sinclair BJ, Borkent A, Wood DM. The male genital tract and aedeagal components of the Diptera with a discussion of their phylogenetic significance. *Zoological Journal of the Linnean Society* 2007, **150**(4)**:** 711-742.

242. Singh OP, Chandra D, Nanda N, Sharma SK, Htun PT, Adak T*, et al.* On the conspecificity of *Anopheles fluviatilis* species S with *Anopheles minimus* species C. *J Biosci* 2006, **31**(5)**:** 671-677.

243. Sither CB, Hopkins VE, Harrison BA, Bintz BJ, Hickman EY, Brown JS*, et al.* Differentiation of *Aedes atlanticus* and *Aedes tormentor* by restriction fragment length polymorphisms of the second internal transcribed spacer. *Journal of the American Mosquito Control Association* 2013, **29**(4)**:** 376-379.

244. Somboon P, Walton C, Sharpe RG, Higa Y, Tuno N, Tsuda Y*, et al.* Evidence for a new sibling species of *Anopheles minimus* from the Ryukyu Archipelago, Japan. *Journal of the American Mosquito Control Association* 2001, **17**(2)**:** 98-113.

245. Song S, Zhao T, Dong Y, Jiang S, Lu B. mtDNA-16S rRNA sequences of the *Culex pipiens* complex and their phylogenetic analyses. *Acta Zootaxonomica Sinica* 2002, **27**(4)**:** 665-671.

246. Sota T, Mogi M. Origin of pitcher plant mosquitoes in *Aedes (Stegomyia)*: A molecular phylogenetic analysis using mitochondrial and nuclear gene sequences. *Journal of Medical Entomology* 2006, **43**(5)**:** 795-800.

247. Staley M, Dorman KS, Bartholomay LC, Fernandez-Salas I, Farfan-Ale JA, Lorono-Pino MA*, et al.* Universal primers for the amplification and sequence analysis of actin-1 from diverse mosquito species. *Journal of the American Mosquito Control Association* 2010, **26**(2)**:** 214-218.

248. Summa K, Urbanski JM, Zhao X, Poelchau M, Armbruster P. Cloning and sequence analysis of the circadian clock genes period and timeless in *Aedes albopictus* (Diptera: Culicidae). *J Med Entomol* 2012, **49**(3)**:** 777-782.

249. Sun E, Zhang X, Qin Z. Sequence analysis of the second internal transcription spacer of ribosomal DNA from *Anopheles dirus* and *Anopheles stephensi*. *Chinese Journal of Zoonoses* 2007.

250. Sun H, Sun L, He J, Shen B, Yu J, Chen C*, et al.* Cloning and characterization of ribosomal protein S29, a deltamethrin resistance associated gene from *Culex pipiens pallens*. *Parasitology research* 2011, **109**(6)**:** 1689-1697.

251. Surendran SN, Sarma DK, Jude PJ, Kemppainen P, Kanthakumaran N, Gajapathy K*, et al.* Molecular characterization and identification of members of the *Anopheles subpictus* complex in Sri Lanka. *Malaria journal* 2013, **12:** 304.

252. Surendran SN, Singh OP, Jude PJ, Ramasamy R. Genetic evidence for malaria vectors of the *Anopheles sundaicus* complex in Sri Lanka with morphological characteristics attributed to *Anopheles subpictus* species B. *Malaria journal* 2010, **9:** 343.

253. Taai K, Baimai V, Saeung A, Thongsahuan S, Min GS, Otsuka Y*, et al.* Genetic compatibility between *Anopheles lesteri* from Korea and *Anopheles paraliae* from Thailand. *Memorias do Instituto Oswaldo Cruz* 2013, **108**(3)**:** 312-320.

254. Taira K, Toma T, Tamashiro M, Miyagi I. DNA barcoding for identification of mosquitoes (Diptera: Culicidae) from the Ryukyu Archipelago, Japan. *Medical Entomology and Zoology* 2012, **63**(4)**:** 289-306.

255. Takano KT, Ngoc THN, Binh THN, Sunahara T, Yasunami M, Manh DN*, et al.* Partial mitochondrial DNA sequences suggest the existence of a cryptic species within the *leucosphyrus* group of the genus *Anopheles* (Diptera: Culicidae), forest malaria vectors, in northern Vietnam. *Parasites & vectors* 2010, **3**.

256. Talbalaghi A, Shaikevich E. Molecular approach for identification of mosquito species (Diptera: Culicidae) in Province of Alessandria, Piedmont, Italy. *Eur J Entomol* 2011, **108**(1)**:** 35-40.

257. Tan W, Zhao T, Dong Y, Wang Z, Li C. Molecule identification and rDNA ITS2 sequence analysis of *Anopheles* from different provinces and habitats. *Acta Parasitol Med Entomol Sin* 2011, **17:** 66-70.

258. Taylor DB. Genetics of interspecific hybridization in the *triseriatus* and *zoosophus* groups of *Aedes (Protomacleaya)* (Diptera, Culicidae). *Ann Entomol Soc Am* 1990, **83**(6)**:** 1181-1191.

259. Thongsahuan S, Baimai V, Otsuka Y, Saeung A, Tuetun B, Jariyapan N*, et al.* Karyotypic variation and geographic distribution of *Anopheles campestris*-like (Diptera: Culicidae) in Thailand. *Memorias do Instituto Oswaldo Cruz* 2009, **104**(4)**:** 558-566.

260. Toma T, Miyagi I, Crabtree MB, Miller BR. Investigation of the *Aedes (Stegomyia) flavopictus* complex (Diptera: Culicidae) in Japan by sequence analysis of the internal transcribed spacers of ribosomal DNA. *Journal of Medical Entomology* 2002, **39**(3)**:** 461-468.

261. Torres EP, Foley DH, Bryan JH. Molecular systematics of the Philippine malaria vector *Anopheles flavirostris*. *Med Vet Entomol* 2006, **20**(1)**:** 44-52.

262. Trindade DB, Scarpassa VM. Genetic differentiation and diagnostic loci among *Anopheles*

(Nyssorhynchus) rangeli, *An. (Nys.) nuneztovari*, and *An. (Nys.) dunhami* (Diptera: Culicidae) in the Brazilian Amazon. *Journal of Medical Entomology* 2002, **39**(4)**:** 613-620.

263. Tuten HC, Bridges WC, Adler PH. Comparative morphology of the pyloric armature of adult mosquitoes (Diptera: Culicidae). *Arthropod Struct Dev* 2012, **41**(5)**:** 475-481.

264. Tyagi BK, Hiriyan J, Tewari SC, Ayanar K, Samuel PP, Arunachalam N*, et al.* Description of a new species, *Anopheles pseudosundaicus* (Diptera: Culicidae) from Kerala, India. *Zootaxa* 2009(2219)**:** 49-60.

265. Versteirt V, Boyer S, Damiens D, De Clercq EM, Dekoninck W, Ducheyne E*, et al.* Nationwide inventory of mosquito biodiversity (Diptera: Culicidae) in Belgium, Europe. *Bulletin of Entomological Research* 2013, **103**(2)**:** 193-203.

266. Vesgueiro FT, Demari-Silva B, Malafronte RD, Sallum MAM, Marrelli MT. Intragenomic variation in the second internal transcribed spacer of the ribosomal DNA of species of the genera *Culex* and *Lutzia* (Diptera: Culicidae). *Memorias do Instituto Oswaldo Cruz* 2011, **106**(1)**:** 1-8.

267. Wagner R, Bartak M, Borkent A, Courtney G, Goddeeris B, Haenni JP*, et al.* Global diversity of dipteran families (Insecta Diptera) in freshwater (excluding Simulidae, Culicidae, Chironomidae, Tipulidae and Tabanidae). *Hydrobiologia* 2008, **595:** 489-519.

268. Walter KS, Brown JE, Powell JR. Microhabitat Partitioning of *Aedes simpsoni* (Diptera: Culicidae). *Journal of Medical Entomology* 2014, **51**(3)**:** 596-604.

269. Walton C, Somboon P, O'Loughlin SM, Zhang S, Harbach RE, Linton YM*, et al.* Genetic diversity and molecular identification of mosquito species in the *Anopheles maculatus* group using the ITS2 region of rDNA. *Infection Genetics and Evolution* 2007, **7**(1)**:** 93-102.

270. Wang G, Li CX, Guo XX, Xing D, Dong YD, Wang ZM*, et al.* Identifying the main mosquito species in China based on DNA barcoding. *PLoS One* 2012, **7**(10).

271. Wang X-z, Townson H. Molecular variation and phylogeny of the members of *minimus* group of *Anopheles*. *Chinese Journal of Vector Biology and Control* 2001, **12**(4)**:** 252-256.

272. Weitzel T, Collado A, Jost A, Pietsch K, Storch V, Becker N. Genetic differentiation of populations within the *Culex pipiens* complex and phylogeny of related species. *Journal of the American Mosquito Control Association* 2009, **25**(1)**:** 6-17.

273. Wesson DM, Porter CH, Collins FH. Sequence and secondary structure comparisons of ITS rDNA in mosquitoes (Diptera: Culicidae). *Mol Phylogenet Evol* 1992, **1**(4)**:** 253-269.

274. Wilkerson RC, Foster PG, Li C, Sallum MAM. Molecular phylogeny of neotropical *Anopheles (Nyssorhynchus) albitarsis* species complex (Diptera: Culicidae). *Ann Entomol Soc Am* 2005,

98(6)**:** 918-925.

275. Wu J, Ma Y, Ma Y. Phylogenetic relationship among the species of *Anopheles* subgenus *Cellia* (Diptera: Culicidae) in China: inferred by mitochondrial and ribosomal DNA sequences. *Acta Entomol Sinica* 2010, **53**(9)**:** 1030-1038.

276. Xia A, Sharakhova MV, Sharakhov IV. Reconstructing an inversion history in the *Anopheles gambiae* complex. *Lect N Bioinformat* 2007, **4751:** 136-+.

277. Ya-Jun MA, Jing WU, Ying MA. Phylogenetic relationships among the species of *Anopheles* subgenus *Cellia* (Diptera: Culicidae) in China inferred by ITS2 sequences of ribosomal DNA. *Entomotaxonomia* 2011, **33**(4)**:** 245-256.

278. Ying MA, Ya jun MA, Lin L, Wang Y. Phylogenetic relationship among some species of genus *Anopheles* subgenus *Anopheles* (Diptera: Culicidae) in China: based on rDNA-ITS2 sequences. *Chinese Journal of Vector Biology & Control* 2013, **24**(5)**:** 382-388.

279. Young CLE, Chaon BC, Shan QA, Lobo NF, Collins FH. A checklist of the mosquitoes of Indiana with notes on the cryptic species complexes *Anopheles quadrimaculatus* s.l. and *Anopheles punctipennis*. *Journal of the American Mosquito Control Association* 2008, **24**(3)**:** 450-452.

280. Yssouf A, Socolovschi C, Flaudrops C, Ndiath MO, Sougoufara S, Dehecq JS*, et al.* Matrix-assisted laser desorption Ionization - time of flight mass spectrometry: An emerging tool for the rapid identification of mosquito vectors. *PLoS One* 2013, **8**(8).

281. Zavortink TJ. Classical taxonomy of mosquitos - a memorial to Belkin,John, N. *Journal of the American Mosquito Control Association* 1990, **6**(4)**:** 593-599.

282. Zhan LL, Ding Z, Qian YH, Zeng QT. Convergent intron loss of MRP1 in *Drosophila* and mosquito species. *J Hered* 2012, **103**(1)**:** 147-151.

283. Zhao T, Lu B. The clasdics of *Culex pipiens* complex. *Acta Zootaxonomica Sinica* 1999, **24**(2)**:** 206-210.

284. Zomuanpuii R, Ringngheti L, Brindha S, Gurusubramanian G, Kumar NS. ITS2 characterization and *Anopheles* species identification of the subgenus *Cellia*. *Acta tropica* 2013, **125**(3)**:** 309-319.

Part2: Reference list for the 374 source papers used to acquire the data on vectors.

1. Abduselam N, Zeynudin A, Berens-Riha N, Seyoum D, Pritsch M, Tibebu H*, et al.* Similar trends of susceptibility in *Anopheles arabiensis* and *Anopheles pharoensis* to *Plasmodium vivax* infection in Ethiopia. *Parasites & vectors* 2016, **9**.

2. Afolabi JS, Ewing SA, Wright RE, Wright JC. *Culex erraticus*: a host for *Dirofilaria immitis*. *Journal of the American Mosquito Control Association* 1989, **5**(1)**:** 109.

3. Afrane YA, Bonizzoni M, Yan GY. Secondary malaria vectors of Sub-Saharan Africa: Threat to malaria elimination on the continent? *Current Topics in Malaria* 2016**:** 473-490.

4. Agwu EJ, Igbinosa IB, Isaac C. Entomological assessment of yellow fever-epidemic risk indices in Benue State, Nigeria, 2010-2011. *Acta tropica* 2016, **161:** 18-25.

5. Al Ahmed AM, Badjah-Hadj-Ahmed AY, Al Othman ZA, Sallam MF. Identification of wild collected mosquito vectors of diseases using gas chromatography-mass spectrometry in Jazan province, Saudi Arabia. *Journal of Mass Spectrometry* 2013, **48**(11)**:** 1170-1177.

6. Alam MS, Chakma S, Khan WA, Glass GE, Mohon AN, Elahi R*, et al.* Diversity of anopheline species and their Plasmodium infection status in rural Bandarban, Bangladesh. *Parasites & vectors* 2012, **5**.

7. Al-Amin HM, Elahi R, Mohon AN, Kafi MAH, Chakma S, Lord JS*, et al.* Role of underappreciated vectors in malaria transmission in an endemic region of Bangladesh-India border. *Parasites & vectors* 2015, **8**.

8. Alencar J, de Mello CF, Barbosa LS, Gil-Santana HR, Maia DD, Marcondes CB*, et al.* Diversity of yellow fever mosquito vectors in the Atlantic Forest of Rio de Janeiro, Brazil. *Revista da Sociedade Brasileira de Medicina Tropical* 2016, **49**(3)**:** 351-356.

9. Aliota MT, Peinado SA, Osorio JE, Bartholomay LC. *Culex pipiens* and *Aedes triseriatus* mosquito susceptibility to Zika virus. *Emerging infectious diseases* 2016, **22**(10)**:** 1857-1859.

10. Almeida APG, Freitas FB, Novo MT, Sousa CA, Rodrigues JC, Alves R*, et al.* Mosquito Surveys and West Nile virus screening in two different areas of Southern Portugal, 2004-2007. *Vector-Borne Zoonot* 2010, **10**(7)**:** 673-680.

11. Altamiranda-Saavedra M, Arboleda S, Parra JL, Peterson AT, Correa MM. Potential distribution of mosquito vector species in a primary malaria endemic region of Colombia. *PLoS One* 2017, **12**(6).

12. Alto BW, Wiggins K, Eastmond B, Velez D, Lounibos LP, Lord CC. Transmission risk of two chikungunya lineages by invasive mosquito vectors from Florida and the Dominican Republic. *PLoS neglected tropical diseases* 2017, **11**(7).

13. anderson CR, Osornomesa E. The laboratory transmission of yellow fever virus by *Haemagogus splendens*. *Am J Trop Med* 1946, **26**(5)**:** 613-618.

14. anderson JF, andreadis TG, Main AJ, Ferrandino FJ, Vossbrinck CR. West Nile virus from female and male mosquitoes (Diptera: Culicidae) in subterranean, ground, and canopy habitats in Connecticut. *Journal of Medical Entomology* 2006, **43**(5)**:** 1010-1019.

15. anderson JF, McKnight S, Ferrandino FJ. *Aedes japonicus japonicus* and associated woodland species attracted to centers for disease control and prevention miniature light traps baited with carbon dioxide and the traptech (R) mosquito lure. *Journal of the American Mosquito Control Association* 2012, **28**(3)**:** 184-191.

16. andreadis TG, anderson JF, Armstrong PM, Main AJ. Isolations of Jamestown Canyon virus (Bunyaviridae: Orthobunyavirus) from field-collected mosquitoes (Diptera: Culicidae) in Connecticut, USA: A ten-year analysis, 1997-2006. *Vector-Borne Zoonot* 2008, **8**(2)**:** 175-188.

17. andreadis TG, anderson JF, Tirrell-Peck SJ. Multiple isolations of eastern equine encephalitis and Highlands J viruses from mosquitoes (Diptera: Culicidae) during a 1996 epizootic in southeastern Connecticut. *Journal of Medical Entomology* 1998, **35**(3)**:** 296-302.

18. andreadis TG, anderson JF, Vossbrinck CR, Main AJ. Epidemiology of West Nile virus in Connecticut: A five-year analysis of mosquito data 1999-2003. *Vector-Borne Zoonot* 2004, **4**(4)**:** 360-378.

19. andreadis TG, Armstrong PM, anderson JF, Main AJ. Spatial-Temporal Analysis of Cache Valley Virus (Bunyaviridae: Orthobunyavirus) Infection in Anopheline and Culicine Mosquitoes (Diptera: Culicidae) in the Northeastern United States, 1997-2012. *Vector-Borne Zoonot* 2014, **14**(10)**:** 763-773.

20. Antonio-Nkondjio C, Kerah CH, Simard F, Awono-Ambene P, Chouaibou M, Tchuinkam T*, et al.* Complexity of the malaria vectorial system in Cameroon: Contribution of secondary vectors to malaria transmission. *Journal of Medical Entomology* 2006, **43**(6)**:** 1215-1221.

21. Antonio-Nkondjio C, Simard F, Awono-Ambene P, Ngassam P, Toto JC, Tchuinkam T*, et al.* Malaria vectors and urbanization in the equatorial forest region of south Cameroon. *Transactions of the Royal Society of Tropical Medicine and Hygiene* 2005, **99**(5)**:** 347-354.

22. Anyanwu IN, Agbede RIS, Ajanusi OJ, Umoh JU, Ibrahim NDG. The incrimination of *Aedes (stegomyia) aegypti* as the vector of *Dirofilaria repens* in Nigeria. *Veterinary parasitology* 2000, **92**(4)**:** 319-327.

23. Appawu MA, Baffoe-Wilmot A, Afari EA, Dunyo S, Koram KA, Nkrumah FK. Malaria vector studies in two ecological zones in Southern Ghana. *Afr Entomol* 2001, **9**(1)**:** 59-65.

24. Arum SO, Weldon CW, Orindi B, Landmann T, Tchouassi DP, Affognon HD*, et al.* Distribution and diversity of the vectors of Rift Valley fever along the livestock movement routes in the northeastern and coastal regions of Kenya. *Parasites & vectors* 2015, **8**.

25. Ataie A, Moosa-Kazemi SH, Vatandoost H, Yaghoobi-Ershadi MR, Bakhshi H, Anjomruz M. Assessing the susceptibility status of mosquitoes (Diptera: Culicidae) in a dirofilariasis focus, northwestern Iran. *Journal of arthropod-borne diseases* 2015, **9**(1)**:** 7-21.

26. Azuolas JK, Wishart E, Bibby S, Ainsworth C. Isolation of Ross River virus from mosquitoes and from horses with signs of musculo-skeletal disease. *Aust Vet J* 2003, **81**(6)**:** 344-347.

27. Ba Y, Sall AA, Diallo D, Mondo M, Girault L, Dia I*, et al.* Re-emergence of Rift Valley fever virus In Barkedji (Senegal, West Africa) In 2002-2003: Identification of new vectors and epidemiological implications. *Journal of the American Mosquito Control Association* 2012, **28**(3)**:** 170-178.

28. Bagavan A, Rahuman AA. Evaluation of larvicidal activity of medicinal plant extracts against three mosquito vectors. *Asian Pacific journal of tropical medicine* 2011, **4**(1)**:** 29-34.

29. Balenghien T, Vazeille M, Grandadam M, Schaffner F, Zeller H, Reiter P*, et al.* Vector Competence of Some French *Culex* and *Aedes* mosquitoes for West Nile virus. *Vector-Borne Zoonot* 2008, **8**(5)**:** 589-595.

30. Barbosa LMC, Souto RNP, Ferreira RMD, Scarpassa VM. Behavioral patterns, parity rate and natural infection analysis in anopheline species involved in the transmission of malaria in the northeastern Brazilian Amazon region. *Acta tropica* 2016, **164:** 216-225.

31. Bargues MD, Morchon R, Latorre JM, Cancrini G, Mas-Coma S, Simon F. Ribosomal DNA second internal transcribed spacer sequence studies of Culicid vectors from an endemic area of *Dirofilaria immitis* in Spain. *Parasitology research* 2006, **99**(3)**:** 205-213.

32. Barrera R, MacKay A, Amador M, Vasquez J, Smith J, Diaz A*, et al.* Mosquito vectors of West Nile virus during an epizootic outbreak in Puerto Rico. *Journal of Medical Entomology* 2010, **47**(6)**:** 1185-1195.

33. Bashawri LA, Mandil AM, Bahnassy AA, Al-Shamsi MA, Bukhari HA. Epidemiological profile of malaria in a university hospital in the eastern region of Saudi Arabia. *Saudi Med J* 2001, **22**(2)**:** 133-138.

34. Beebe NW, Russell T, Burkot TR, Cooper RD. *Anopheles punctulatus* group: Evolution,

distribution, and control. *Annual Review of Entomology, Vol 60* 2015, **60:** 335-350.

35. Beier JC, Muller GC, Gu WD, Arheart KL, Schlein Y. Attractive toxic sugar bait (ATSB) methods decimate populations of *Anopheles* malaria vectors in arid environments regardless of the local availability of favoured sugar-source blossoms. *Malaria journal* 2012, **11**.

36. Beng TS, Ahmad R, Hisam RSR, Heng SK, Leaburi J, Ismail Z*, et al.* Molecular xenomonitoring of filarial infection in Malaysian mosquitoes under the national program for elimination of lymphatic filariasis. *Se Asian J Trop Med* 2016, **47**(4)**:** 617-624.

37. Bharati M, Saha D. Insecticide susceptibility status and major detoxifying enzymes' activity in *Aedes albopictus* (Skuse), vector of dengue and chikungunya in northern part of West Bengal, India. *Acta tropica* 2017, **170:** 112-119.

38. Bingham AM, Burkett-Cadena ND, Hassan HK, Unnasch TR. Vector Competence and Capacity of Culex erraticus (Diptera: Culicidae) for Eastern Equine Encephalitis Virus in the Southeastern United States. *J Med Entomol* 2016, **53**(2)**:** 473-476.

39. Blackmore CGM, Blackmore MS, Grimstad PR. Role of *Anopheles quadrimaculatus* and *Coquillettidia perturbans* (Diptera: Culicidae) in the transmission cycle of Cache Valley virus (Bunyaviridae: Bunyavirus) in the Midwest, USA. *Journal of Medical Entomology* 1998, **35**(5)**:** 660-664.

40. Bockova E, Iglodhova A, Kocisova A. Potential mosquito (Diptera:Culicidae) vector of *Dirofilaria repens* and *Dirofilaria immitis* in urban areas of Eastern Slovakia. *Parasitology research* 2015, **114**(12)**:** 4487-4492.

41. Bockova E, Rudolf I, Kocisova A, Betasova L, Venclikova K, Mendel J*, et al. Dirofilaria repens* microfilariae in *Aedes vexans* mosquitoes in Slovakia. *Parasitology research* 2013, **112**(10)**:** 3465-3470.

42. Borland EM, Ledermann JP, Powers AM. *Culex tarsalis* mosquitoes as vectors of highlands J Virus. *Vector-Borne Zoonot* 2016, **16**(8)**:** 558-565.

43. Boukraa S, de la Grandiere MA, Bawin T, Raharimalala FN, Zimmer JY, Haubruge E*, et al.* Diversity and ecology survey of mosquitoes potential vectors in Belgian equestrian farms: A threat prevention of mosquito-borne equine arboviruses. *Preventive veterinary medicine* 2016, **124:** 58-68.

44. Boyd AM, Kay BH. Assessment of the potential of dogs and cats as urban reservoirs of Ross River and Barmah Forest viruses. *Aust Vet J* 2002, **80**(1-2)**:** 83-86.

45. Brock PM, Fornace KM, Parmiter M, Cox J, Drakeley CJ, Ferguson HM*, et al. Plasmodium knowlesi* transmission: integrating quantitative approaches from epidemiology and ecology to

understand malaria as a zoonosis. *Parasitology* 2016, **143**(4)**:** 389-400.

46. Brustolin M, Talavera S, Nunez A, Santamaria C, Rivas R, Pujol N*, et al.* Rift Valley fever virus and European mosquitoes: vector competence of *Culex pipiens* and *Stegomyia albopicta* (= *Aedes albopictus*). *Med Vet Entomol* 2017.

47. Brustolin M, Talavera S, Santamaria C, Rivas R, Pujol N, Aranda C*, et al. Culex pipiens* and *Stegomyia albopicta* (=*Aedes albopictus*) populations as vectors for lineage 1 and 2 West Nile virus in Europe. *Medical and Veterinary Entomology* 2016, **30**(2)**:** 166-173.

48. Burke A, Dandalo L, Munhenga G, Dahan-Moss Y, Mbokazi F, Ngxongo S*, et al.* A new malaria vector mosquito in South Africa. *Scientific Reports* 2017, **7**.

49. Burkot TR, Graves PM. The value of vector-based estimates of malaria transmission. *Ann Trop Med Parasit* 1995, **89**(2)**:** 125-134.

50. Calvez E, Guillaumot L, Girault D, Richard V, O'Connor O, Paoaafaite T*, et al.* Dengue-1 virus and vector competence of *Aedes aegypti* (Diptera: Culicidae) populations from New Caledonia. *Parasites & vectors* 2017, **10**.

51. Calzolari M, Ze-Ze L, Vazquez A, Seco MPS, Amaro F, Dottori M. Insect-specific flaviviruses, a worldwide widespread group of viruses only detected in insects. *Infection Genetics and Evolution* 2016, **40:** 381-388.

52. Cancrini G, Magi M, Gabrielli S, Arispici M, Tolari F, Dell'Omodarme M*, et al.* Natural vectors of Dirofilariasis in rural and urban areas of the Tuscan Region, central Italy. *Journal of Medical Entomology* 2006, **43**(3)**:** 574-579.

53. Cardoso JD, de Almeida MAB, dos Santos E, da Fonseca DF, Sallum MAM, Noll CA*, et al.* Yellow fever Virus in Haemagogus leucocelaenus and Aedes serratus Mosquitoes, Southern Brazil, 2008. *Emerging infectious diseases* 2010, **16**(12)**:** 1918-1924.

54. Chapman GE, Archer D, Torr S, Solomon T, Baylis M. Potential vectors of equine arboviruses in the UK. *Veterinary Record* 2017, **180**(1)**:** 19-+.

55. Charan SS, Pawar KD, Gavhale SD, Tikhe CV, Charan NS, Angel B*, et al.* Comparative analysis of midgut bacterial communities in three aedine mosquito species from dengue-endemic and non-endemic areas of Rajasthan, India. *Medical and Veterinary Entomology* 2016, **30**(3)**:** 264-277.

56. Chevalier V, Mondet B, Diaite A, Lancelot R, Fall AG, Poncon N. Exposure of sheep to mosquito bites: possible consequences for the transmission risk of Rift Valley fever in Senegal. *Medical and Veterinary Entomology* 2004, **18**(3)**:** 247-255.

57. Chiang GL, Samarawickrema WA, Mak JW, Cheong WH, Sulaiman I, Yap HH. Field and laboratory observations on *Coquillettidia crassipes* in relation to transmission of Brugia-Malayi in Peninsular Malaysia. *Ann Trop Med Parasit* 1986, **80**(1)**:** 117-121.

58. Christofferson RC, Mores CN. A role for vector control in dengue vaccine programs. *Vaccine* 2015, **33**(50)**:** 7069-7074.

59. Coffey LL, Failloux AB, Weaver SC. Chikungunya virus-vector interactions. *Viruses-Basel* 2014, **6**(11)**:** 4628-4663.

60. Cohen SB, Lewoczko K, Huddleston DB, Moody E, Mukherjee S, Dunn JR*, et al.* Host feeding patterns of potential vectors of Eastern Equine Encephalitis virus at an epizootic focus in Tennessee. *American Journal of Tropical Medicine and Hygiene* 2009, **81**(3)**:** 452-456.

61. Collins FH, Paskewitz SM. Malaria - Current and future-prospects for control. *Annual Review of Entomology* 1995, **40:** 195-219.

62. Conde M, Pareja PX, Orjuela LI, Ahumada ML, Duran S, Jara JA*, et al.* Larval habitat characteristics of the main malaria vectors in the most endemic regions of Colombia: potential implications for larval control. *Malaria journal* 2015, **14**.

63. Cook S, Moureau G, Harbach RE, Mukwaya L, Goodger K, Ssenfuka F*, et al.* Isolation of a novel species of flavivirus and a new strain of *Culex flavivirus* (Flaviviridae) from a natural mosquito population in Uganda. *Journal of General Virology* 2009, **90:** 2669-2678.

64. Cooper RD, Edstein MD, Frances SP, Beebe NW. Malaria vectors of Timor-Leste. *Malaria journal* 2010, **9**.

65. Cooper RD, Waterson DGE, Frances SP, Beebe NW, Pluess B, Sweeney AW. Malaria vectors of Papua New Guinea. *International journal for parasitology* 2009, **39**(13)**:** 1495-1501.

66. Costanzo KS, Muturi EJ, Montgomery AV, Alto BW. Effect of oral infection of La Crosse virus on survival and fecundity of native *Ochlerotatus triseriatus* and invasive *Stegomyia albopicta*. *Medical and Veterinary Entomology* 2014, **28**(1)**:** 77-84.

67. Couto-Lima D, Madec Y, Bersot MI, Campos SS, Motta MD, dos Santos FB*, et al.* Potential risk of re-emergence of urban transmission of yellow fever virus in Brazil facilitated by competent Aedes populations. *Scientific Reports* 2017, **7**.

68. Cramer JP. Plasmodium knowlesi malaria: Overview focussing on travel-associated infections. *Curr Infect Dis Rep* 2015, **17**(3).

69. Cunze S, Koch LK, Kochmann J, Klimpel S. *Aedes albopictus* and *Aedes japonicus* - two invasive mosquito species with different temperature niches in Europe (vol 9, 573, 2016).

Parasites & vectors 2016, **9**.

70. Cuong DM, Beebe NW, Van NTV, Tao LQ, Chau TL, Dung VN*, et al.* Vectors and malaria transmission in deforested, rural communities in north-central Vietnam. *Malaria journal* 2010, **9**.

71. Cupp EW, Hassan HK, Yue X, Oldland WK, Lilley BM, Unnasch TR. West Nile virus infection in mosquitoes in the Mid-South USA, 2002-2005. *Journal of Medical Entomology* 2007, **44**(1)**:** 117-125.

72. Danabalan R, Monaghan MT, Ponsonby DJ, Linton YM. Occurrence and host preferences of *Anopheles maculipennis* group mosquitoes in England and Wales. *Medical and Veterinary Entomology* 2014, **28**(2)**:** 169-178.

73. Day JF, Curtis GA. Blood feeding and oviposition by *Culex nigripalpus* (Diptera: Culicidae) before, during, and after a widespread St. Louis encephalitis virus epidemic in Florida. *Journal of Medical Entomology* 1999, **36**(2)**:** 176-181.

74. Day JF, Stark LM. Transmission patterns of St Louis encephalitis and eastern equine encephalitis viruses in Florida: 1978-1993. *Journal of Medical Entomology* 1996, **33**(1)**:** 132-139.

75. Day JF, Tabachnick WJ, Smartt CT. Factors that influence the transmission of West Nile virus in Florida. *Journal of Medical Entomology* 2015, **52**(5)**:** 743-754.

76. Deardorff ER, Estrada-Franco JG, Freier JE, Navarro-Lopez R, Da Rosa AT, Tesh RB*, et al.* Candidate vectors and rodent hosts of Venezuelan Equine Encephalitis virus, Chiapas, 2006-2007. *American Journal of Tropical Medicine and Hygiene* 2011, **85**(6)**:** 1146-1153.

77. Deitz KC, Athrey GA, Jawara M, Overgaard HJ, Matias A, Slotman MA. Genome-wide divergence in the West-African malaria vector *Anopheles melas*. *G3-Genes Genom Genet* 2016, **6**(9)**:** 2867-2879.

78. Derodaniche E, Galindo P. Isolation of yellow fever virus from *Haemagogus mesodentatus*, *H. Equinus* and *Sabethes chloropterus* captured in Guatemala In 1956. *American Journal of Tropical Medicine and Hygiene* 1957, **6**(2)**:** 232-237.

79. Derodaniche E, Galindo P, Johnson CM. Isolation of yellow fever virus from *Haemagogus lucifer*, *H. Equinus*, *H. spegazzinii* Falco, *Sabethes chloropterus* and *Anopheles neivai* captured in Panama in the fall of 1956. *American Journal of Tropical Medicine and Hygiene* 1957, **6**(4)**:** 681-685.

80. Dev V, Adak T, Singh OP, Nanda N, Baidya BK. Malaria transmission in Tripura: disease distribution & determinants. *Indian Journal of Medical Research* 2015, **142:** 12-22.

81. Dia I, Konate L, Samb B, Sarr JB, Diop A, Rogerie F*, et al.* Bionomics of malaria vectors and

relationship with malaria transmission and epidemiology in three physiographic zones in the Senegal River Basin. *Acta tropica* 2008, **105**(2)**:** 145-153.

82. Diagne CT, Diallo D, Faye O, Ba Y, Faye O, Gaye A*, et al.* Potential of selected Senegalese *Aedes* spp. mosquitoes (Diptera: Culicidae) to transmit Zika virus. *BMC infectious diseases* 2015, **15**.

83. Diallo D, Talla C, Ba Y, Dia I, Sall AA, Diallo M. Temporal distribution and spatial pattern of abundance of the Rift Valley fever and West Nile fever vectors in Barkedji, Senegal. *Journal of Vector Ecology* 2011, **36**(2)**:** 426-436.

84. Dibo MR, de Menezes RMT, Ghirardelli CP, Mendonca AL, Neto FC. The presence of Culicidae species in medium-sized cities in the State of Sao Paulo, Brazil and the risk of West Nile fever and other arbovirus infection. *Revista da Sociedade Brasileira de Medicina Tropical* 2011, **44**(4)**:** 496-503.

85. Duchemin JB, Mee PT, Lynch SE, Vedururu R, Trinidad L, Paradkar P. Zika vector transmission risk in temperate Australia: a vector competence study. *Virology journal* 2017, **14**.

86. Dutta P, Khan SA, Chetry S, Dev V, Sarmah CK, Mahanta J. First evidence of dengue virus infection in wild caught mosquitoes during an outbreak in Assam, Northeast India. *Journal of vector borne diseases* 2015, **52**(4)**:** 293-298.

87. Dutton TJ, Sinkins SP. Filarial susceptibility and effects of *Wolbachia* in *Aedes pseudoscutellaris* mosquitoes. *Medical and Veterinary Entomology* 2005, **19**(1)**:** 60-65.

88. Eldridge BF, Lanzaro GC, Campbell GL, Reeves WC, Hardy JL. Occurrence and evolutionary significance of a California Encephalitis-Like Virus In Aedes-Squamiger (Diptera, Culicidae). *Journal of Medical Entomology* 1991, **28**(5)**:** 645-651.

89. Elissa N, Karch S, Bureau P, Ollomo B, Lawoko M, Yangari P*, et al.* Malaria transmission in a region of savanna-forest mosaic, Haut-Ogooue, Gabon. *Journal of the American Mosquito Control Association* 1999, **15**(1)**:** 15-23.

90. Ellis BR, Sang RC, Horne KM, Higgs S, Wesson DM. yellow fever virus susceptibility of two mosquito vectors from Kenya, East Africa. *Transactions of the Royal Society of Tropical Medicine and Hygiene* 2012, **106**(6)**:** 387-389.

91. Enayati A, Hemingway J. Malaria management: Past, present, and future. *Annual Review of Entomology* 2010, **55:** 569-591.

92. Endy TP, Nisalak A. Japanese encephalitis virus: Ecology and epidemiology. *Curr Top Microbiol* 2002, **267:** 11-48.

93. Evans MV, Dallas TA, Han BA, Murdock CC, Drake JM. Data-driven identification of potential Zika virus vectors. *eLife* 2017, **6**.

94. Eyraud-Griffet A, Pradel J, Chalvet-Monfray K, Bicout DJ. Climatic conditions and probability of the presence of mosquitos, Disease Vectors. *Rev Epid San Anim* 2009(56)**:** 161-174.

95. Fakoorziba MR, Vijayan A. Breeding Habitats of *Culex tritaeniorhynchus* (Diptera: Culicidae), A Japanese encephalitis vector, and associated mosquitoes in Mysore, India. *J Entomol Res Soc* 2008, **10:** 1-9.

96. Fall AG, Diaite A, Etter E, Bouyer J, Ndiaye TD, Konate L. The mosquito *Aedes (Aedimorphus) vexans* arabiensis as a probable vector bridging the West Nile virus between birds and horses in Barkedji (Ferlo, Senegal). *Medical and Veterinary Entomology* 2012, **26**(1)**:** 106-111.

97. Fauran P. New epidemiological findings on dengue. *Bulletin de la Societe de pathologie exotique* 1996, **89**(2)**:** 163-165.

98. Faye O, Diallo M, Diop D, Bezeid OE, Ba H, Niang M*, et al.* Rift valley fever outbreak with East-Central African virus lineage in Mauritania, 2003. *Emerging infectious diseases* 2007, **13**(7)**:** 1016-1023.

99. Feng XY, Zhang SS, Huang F, Zhang L, Feng J, Xia ZG*, et al.* Biology, bionomics and molecular biology of *Anopheles sinensis* Wiedemann 1828 (Diptera: Culicidae), main malaria vector in China. *Frontiers in microbiology* 2017, **8**.

100. Fernando AW, Jayakody S, Wijenayake HK, Galappaththy GNL, Yatawara M, Harishchandra J. Species composition and population dynamics of malaria vectors in three previously ignored aquatic systems in Sri Lanka. *Malaria journal* 2016, **15**.

101. Ferreira CAC, Mixao VD, Novo MTLM, Calado MMP, Goncalves LAP, Belo SMD*, et al.* First molecular identification of mosquito vectors of *Dirofilaria immitis* in continental Portugal. *Parasites & vectors* 2015, **8**.

102. Foley DH, Bryan JH, Lawrence GW. The potential of ivermectin to control the malaria vector *Anopheles farauti*. *Transactions of the Royal Society of Tropical Medicine and Hygiene* 2000, **94**(6)**:** 625-628.

103. Fontenille D, Carnevale P. Malaria and dengue vector biology and control in West and Central Africa. *Wag Ur Fron* 2006, **11:** 91-+.

104. Fontenille D, Cohuet A, Awono-Ambene P, Kengne P, Antonio-Nkondjio C, Wondji C*, et al.* Malaria vectors: from the field to genetics - Research in Africa. *Rev Epidemiol Sante* 2005, **53**(3)**:** 283-290.

105. Fortuna C, Remoli ME, Severini F, Di Luca M, Toma L, Fois F*, et al.* Evaluation of vector competence for West Nile virus in Italian *Stegomyia albopicta* (=*Aedes albopictus*) mosquitoes. *Medical and Veterinary Entomology* 2015, **29**(4)**:** 430-433.

106. Frank C, Faber M, Hellenbrand W, Wilking H, Stark K. Important vector-borne infectious diseases among humans in Germany. Epidemiological aspects. *Bundesgesundheitsbla* 2014, **57**(5)**:** 557-567.

107. Freitas FB, Novo MT, Esteves A, de Almeida APG. Species composition and WNV screening of mosquitoes from lagoons in a wetland area of the Algarve, Portugal. *Front Physiol* 2012, **2**.

108. Fritz GN, Engman S, Rodriguez R, Wilkerson RC. Identification of four vectors of human *Plasmodium* spp. by multiplex PCR: *Anopheles rangeli*, *An-strodei*, *An-triannulatus*, and *An-trinkae* (Diptera: Culicidae: Nyssorhynchus). *Journal of Medical Entomology* 2004, **41**(6)**:** 1111-1115.

109. Gajanana A, Rajendran R, Samuel PP, Thenmozhi V, Tsai TF, Kimura-Kuroda J*, et al.* Japanese encephalitis in South Arcot district, Tamil Nadu, India: A three-year longitudinal study of vector abundance and infection frequency. *Journal of Medical Entomology* 1997, **34**(6)**:** 651-659.

110. Gao Q, Beebe NW, Cooper RD. Molecular identification of the malaria vectors *Anopheles anthropophagus* and *Anopheles sinensis* (Diptera: Culicidae) in central China using polymerase chain reaction and appraisal of their position within the *hyrcanus* group. *Journal of Medical Entomology* 2004, **41**(1)**:** 5-11.

111. Gingrich JB, Williams GM. Host feeding patterns of suspected West Nile virus mosquito vectors in Delaware, 2001-2002. *Journal of the American Mosquito Control Association* 2005, **21**(2)**:** 194-200.

112. Glushakova LG, Alto BW, Kim MS, Bradley A, Yaren O, Benner SA. Detection of chikungunya viral RNA in mosquito bodies on cationic (Q) paper based on innovations in synthetic biology. *J Virol Methods* 2017, **246:** 104-111.

113. Goddard LB, Roth AE, Reisen WK, Scott TW. Vector competence of California mosquitoes for West Nile virus. *Emerging infectious diseases* 2002, **8**(12)**:** 1385-1391.

114. Goenaga S, Kenney JL, Duggal NK, Delorey M, Ebel GD, Zhang B*, et al.* Potential for co-Infection of a mosquito-specific Flavivirus, Nhumirim virus, to block West Nile virus Transmission in Mosquitoes. *Viruses-Basel* 2015, **7**(11)**:** 5801-5812.

115. Goertz GP, Vogels CBF, Geertsema C, Koenraadt CJM, Pijlman GP. Mosquito co-infection with Zika and chikungunya virus allows simultaneous transmission without affecting vector competence of *Aedes aegypti*. *PLoS neglected tropical diseases* 2017, **11**(6).

116. Gokhale MD, Paingankar MS, Sudeep AB, Parashar D. Chikungunya virus susceptibility & variation in populations of *Aedes aegypti* (Diptera: Culicidae) mosquito from India. *Indian Journal of Medical Research* 2015, **142:** 33-43.

117. Golding N, Nunn MA, Medlock JM, Purse BV, Vaux AGC, Schafer SM. West Nile virus vector *Culex modestus* established in Southern England. *Parasites & vectors* 2012, **5**.

118. Gomes E, Capinha C, Rocha J, Sousa C. Mapping risk of malaria transmission in mainland Portugal using a mathematical modelling approach. *PLoS One* 2016, **11**(11).

119. Gong HF, DeGaetano AT, Harrington LC. Climate-based models for West Nile *Culex* mosquito vectors in the Northeastern US. *Int J Biometeorol* 2011, **55**(3)**:** 435-446.

120. Gordeev MI, Bezzhonova OV, Moskaev AV. Chromosomal polymorphism in the populations of malaria vector mosquito *Anopheles messeae* at the south of Russian Plain. *Russ J Genet+* 2012, **48**(9)**:** 962-965.

121. Grimstad PR, Paulson SL, Craig GB. Vector competence of *Aedes hendersoni* (Diptera, Culicidae) for La Crosse Virus and evidence of a salivary-gland escape barrier. *Journal of Medical Entomology* 1985, **22**(4)**:** 447-453.

122. Guedes DR, Paiva MH, Donato MM, Barbosa PP, Krokovsky L, Rocha S*, et al.* Zika virus replication in the mosquito *Culex quinquefasciatus* in Brazil. *Emerging microbes & infections* 2017, **6**(8)**:** e69.

123. Gunasekaran K, Sahu SS, Jambulingam P. Estimation of vectorial capacity of *Anopheles minimus* Theobald & *An. fluviatilis* James (Diptera: Culicidae) in a malaria endemic area of Odisha State, India. *Indian Journal of Medical Research* 2014, **140:** 653-659.

124. Guo S, Ling F, Hou J, Wang JN, Fu GM, Gong ZY. Mosquito surveillance revealed lagged effects of mosquito abundance on mosquito-borne disease transmission: A retrospective study in Zhejiang, China. *PLoS One* 2014, **9**(11).

125. Guo XX, Li CX, Deng YQ, Xing D, Liu QM, Wu Q*, et al. Culex pipiens quinquefasciatus*: a potential vector to transmit Zika virus. *Emerging microbes & infections* 2016, **5**.

126. Hall-Mendelin S, Pyke AT, Moore PR, Mackay IM, McMahon JL, Ritchie SA*, et al.* Assessment of local mosquito species incriminates *Aedes aegypti* as the potential vector of Zika virus in Australia. *PLoS neglected tropical diseases* 2016, **10**(9).

127. Hammami P, Tran A, Kemp A, Tshikae P, Kgori P, Chevalier V*, et al.* Rift Valley fever vector diversity and impact of meteorological and environmental factors on *Culex pipiens* dynamics in the Okavango Delta, Botswana. *Parasites & vectors* 2016, **9**.

128. Hanafi HA, Fryauff DJ, Saad MD, Soliman AK, Mohareb EW, Medhat I*, et al.* Virus isolations and high population density implicate *Culex antennatus* (Becker) (Diptera: Culicidae) as a vector of Rift Valley fever virus during an outbreak in the Nile Delta of Egypt. *Acta tropica* 2011, **119**(2-3)**:** 119-124.

129. Hardy JL, Eldridge BF, Reeves WC, Schutz SJ, Presser SB. Isolations of Jamestown Canyon virus (Bunyaviridae, California Serogroup) from mosquitos (Diptera, Culicidae) in the western United-States, 1990-1992. *Journal of Medical Entomology* 1993, **30**(6)**:** 1053-1059.

130. Harley D, Ritchie S, Phillips D, Van den Hurk A. Mosquito isolates of Ross River virus from Cairns, Queensland, Australia. *American Journal of Tropical Medicine and Hygiene* 2000, **62**(5)**:** 561-565.

131. Heitmann A, Jansen S, Luhken R, Leggewie M, Badusche M, Pluskota B*, et al.* Experimental transmission of Zika virus by mosquitoes from central Europe. *Eurosurveillance* 2017, **22**(2)**:** 4-6.

132. Hemmerter S, Slapeta J, van den Hurk AF, Cooper RD, Whelan PI, Russell RC*, et al.* A curious coincidence: mosquito biodiversity and the limits of the Japanese encephalitis virus in Australasia. *BMC Evolutionary Biology* 2007, **7**.

133. Hernandez-Morales A, Arvizu-Gomez JL, Carranza-Alvarez C, Gomez-Luna BE, Alvarado-Sanchez B, Ramirez-Chavez E*, et al.* Larvicidal activity of affinin and its derived amides from *Heliopsis longipes* A. Gray Blake against *Anopheles albimanus* and *Aedes aegypti*. *J Asia-Pac Entomol* 2015, **18**(2)**:** 227-231.

134. Hesson JC, Verner-Carlsson J, Larsson A, Ahmed R, Lundkvist A, Lundstrom JO. *Culex torrentium* mosquito role as major enzootic vector defined by rate of Sindbis virus infection, Sweden, 2009. *Emerging infectious diseases* 2015, **21**(5)**:** 875-878.

135. Hoyos-Lopez R, Suaza-Vasco J, Rua-Uribe G, Uribe S, Gallego-Gomez JC. Molecular detection of flaviviruses and alphaviruses in mosquitoes (Diptera: Culicidae) from coastal ecosystems in the Colombian Caribbean. *Memorias do Instituto Oswaldo Cruz* 2016, **111**(10)**:** 625-634.

136. Hribar LJ, Vlach JJ, Demay DJ, Stark LM, Stoner RL, Godsey MS*, et al.* Mosquitoes infected with West Nile virus in the Florida Keys, Monroe county, Florida, USA. *Journal of Medical Entomology* 2003, **40**(3)**:** 361-363.

137. Huang YM. *Aedes (Stegomyia) bromeliae* (Diptera, Culicidae), the yellow-fever virus vector in East-Africa. *Journal of Medical Entomology* 1986, **23**(2)**:** 196-200.

138. Ilkal MA, Mavale MS, Prasanna Y, Jacob PG, Geevarghese G, Banerjee K. Experimental

studies on the vector potential of certain *Culex* species to West Nile virus. *Indian Journal of Medical Research* 1997, **106:** 225-228.

139. Imai N, White MT, Ghani AC, Drakeley CJ. Transmission and Control of *Plasmodium knowlesi*: A mathematical modelling study. *PLoS neglected tropical diseases* 2014, **8**(7).

140. Iranpour M, Turell MJ, Lindsay LR. Potential for Canadian mosquitoes To transmit Rift Valley fever virus. *Journal of the American Mosquito Control Association* 2011, **27**(4)**:** 363-369.

141. Jackson BT, Brewster CC, Paulson SL. La Crosse virus infection alters blood feeding behavior in *Aedes triseriatus* and *Aedes albopictus* (Diptera: Culicidae). *Journal of Medical Entomology* 2012, **49**(6)**:** 1424-1429.

142. Jansen CC, Prow NA, Webb CE, Hall RA, Pyke AT, Harrower BJ*, et al.* Arboviruses isolated from mosquitoes collected from urban and Peri-Urban areas of eastern Australia. *Journal of the American Mosquito Control Association* 2009, **25**(3)**:** 272-278.

143. Jansen CC, Webb CE, Northill JA, Ritchie SA, Russell RC, Van Den Hurk AF. Vector competence of Australian mosquito species for a north American strain of West Nile virus. *Vector-Borne Zoonot* 2008, **8**(6)**:** 805-811.

144. Jiram AI, Vythilingam I, NoorAzian YM, Yusof YM, Azahari AH, Fong MY. Entomologic investigation of *Plasmodium knowlesi* vectors in Kuala Lipis, Pahang, Malaysia. *Malaria journal* 2012, **11**.

145. Johansen CA, Nisbet DJ, Zborowski P, Van den Hurk AF, Ritchie SA, Mackenzie JS. Flavivirus isolations from mosquitoes collected from western Cape York Peninsula, Australia, 1999-2000. *Journal of the American Mosquito Control Association* 2003, **19**(4)**:** 392-396.

146. Jupille H, Seixas G, Mousson L, Sousa CA, Failloux AB. Zika virus, a new threat for Europe? *PLoS neglected tropical diseases* 2016, **10**(8).

147. Jupp PG. Laboratory Studies on vector capability of *Aedes (Neomelaniconion) unidentatus* Mcintosh and *Aedes (Aedimorphus) dentatus* (Theobald) with West Nile and Sindbis viruses. *S Afr J Med Sci* 1976, **41**(4)**:** 265-269.

148. Jupp PG. The ecology of West Nile virus in South Africa and the occurrence of outbreaks in humans. *Ann Ny Acad Sci* 2001, **951:** 143-152.

149. Kalimuthu K, Panneerselvam C, Chou C, Tseng LC, Murugan K, Tsai KH*, et al.* Control of dengue and Zika virus vector *Aedes aegypti* using the predatory copepod *Megacyclops formosanus*: Synergy with *Hedychium coronarium*-synthesized silver nanoparticles and related histological changes in targeted mosquitoes. *Process Saf Environ* 2017, **109:** 82-96.

150. Kenney JL, Romo H, Duggal NK, Tzeng WP, Burkhalter KL, Brault AC*, et al.* Transmission Incompetence of *Culex quinquefasciatus* and *Culex pipiens pipiens* from North America for Zika virus. *The American journal of tropical medicine and hygiene* 2017, **96**(5)**:** 1235-1240.

151. Keven JB, Reimer L, Katusele M, Koimbu G, Vinit R, Vincent N*, et al.* Plasticity of host selection by malaria vectors of Papua New Guinea. *Parasites & vectors* 2017, **10**.

152. Kibret S, Wilson GG. Increased outdoor biting tendency of *Anopheles arabiensis* and its challenge for malaria control in Central Ethiopia. *Public Health* 2016, **141:** 143-145.

153. Kightlinger L. West Nile Review: 15 Years of human disease in South Dakota, 2002-2016. *South Dakota medicine: the journal of the South Dakota State Medical Association* 2017, **70**(8)**:** 346-351.

154. Killeen GF, Masalu JP, Chinula D, Fotakis EA, Kavishe DR, Malone D*, et al.* Control of malaria vector mosquitoes by insecticide-treated combinations of window screens and eave baffles. *Emerging infectious diseases* 2017, **23**(5)**:** 782-789.

155. Kilpatrick AM, Fonseca DM, Ebel GD, Reddy MR, Kramer LD. Spatial and temporal variation in vector competence of *Culex pipiens* and *Cx. restuans* mosquitoes for West Nile virus. *American Journal of Tropical Medicine and Hygiene* 2010, **83**(5)**:** 357-357.

156. Kim H, Cha GW, Jeong YE, Lee WG, Chang KS, Roh JY*, et al.* Detection of Japanese encephalitis virus genotype V in *Culex orientalis* and *Culex pipiens* (Diptera: Culicidae) in Korea. *PLoS One* 2015, **10**(2).

157. Kim HC, Takhampunya R, Tippayachai B, Chong ST, Park JY, Kim MS*, et al.* Japanese encephalitis virus in Culicine mosquitoes (Diptera: Culicidae) of the Republic of Korea, 2008-2010. *Military medicine* 2015, **180**(2)**:** 158-167.

158. Klein TA, Lima JBP, Tada MS, Miller R. Comparative susceptibility of Anopheline mosquitos in Rondonia, Brazil to infection by *Plasmodium vivax*. *American Journal of Tropical Medicine and Hygiene* 1991, **45**(4)**:** 463-470.

159. Klobucar A, Benic N, Krajcar D, Kosanovic-Licina ML, Tesic V, Merdic E*, et al.* An overview of mosquitoes and emerging arboviral infections in the Zagreb area, Croatia. *Journal of infection in developing countries* 2016, **10**(12)**:** 1286-1293.

160. Kokernot RH, Demorais T, Mcintosh BM, Worth CB, Weinbren MP. Isolation of viruses from mosquitoes collected at Lumbo, Mozambique .1. Lumbo virus, a new virus isolated from *Aedes (Skusea) pembaensis* Theobald. *American Journal of Tropical Medicine and Hygiene* 1962, **11**(5)**:** 678-&.

161. Korgaonkar NS, Kumar A, Yadav RS, Kabadi D, Dash AP. Mosquito biting activity on humans &

detection of *Plasmodium falciparum* infection in *Anopheles stephensi* in Goa, India. *Indian Journal of Medical Research* 2012, **135**(1)**:** 120-126.

162. Kramer LD, Bowen MD, Hardy JL, Reeves WC, Presser SB, Eldridge BF. Vector competence of Alpine, Central Valley, and Coastal mosquitos (Diptera, Culicidae) from California for Jamestown Canyon virus. *Journal of Medical Entomology* 1993, **30**(2)**:** 398-406.

163. Kramer LD, Chin P, Cane RP, Kauffman EB, Mackereth G. Vector competence of New Zealand mosquitoes for selected arboviruses. *American Journal of Tropical Medicine and Hygiene* 2011, **85**(1)**:** 182-189.

164. Kramer LD, Hardy JL, Reeves WC, Presser SB, Bowen MD, Eldridge BF. Vector Competence of selected mosquito species (Diptera, Culicidae) for California strains of Northway virus (Bunyaviridae, Bunyavirus). *Journal of Medical Entomology* 1993, **30**(3)**:** 607-613.

165. Kurucz K, Kepner A, Krtinic B, Zana B, Foldes F, Banyai K*, et al.* First molecular identification of *Dirofilaria* spp. (Onchocercidae) in mosquitoes from Serbia. *Parasitology research* 2016, **115**(8)**:** 3257-3260.

166. Kuwata R, Nga PT, Yen T, Hoshino K, Isawa H, Higa Y*, et al.* Surveillance of Japanese encephalitis virus infection in mosquitoes in Vietnam from 2006 to 2008. *American Journal of Tropical Medicine and Hygiene* 2013, **88**(4)**:** 681-688.

167. Kuwata R, Sugiyama H, Yonemitsu K, Dung NV, Terada Y, Taniguchi M*, et al.* Isolation of Japanese encephalitis virus and a novel insect-specific flavivirus from mosquitoes collected in a cowshed in Japan. *Archives of virology* 2015, **160**(9)**:** 2151-2159.

168. Laporta GZ, Linton YM, Wilkerson RC, Bergo ES, Nagaki SS, Sant'Ana DC*, et al.* Malaria vectors in South America: current and future scenarios. *Parasites & vectors* 2015, **8**.

169. Laporta GZ, Ramos DG, Ribeiro MC, Sallum MAM. Habitat suitability of *Anopheles* vector species and association with human malaria in the Atlantic forest in south-eastern Brazil. *Memorias do Instituto Oswaldo Cruz* 2011, **106:** 239-U249.

170. Le Flohic G, Porphyre V, Barbazan P, Gonzalez JP. Review of climate, landscape, and viral genetics as drivers of the Japanese encephalitis virus ecology. *PLoS neglected tropical diseases* 2013, **7**(9).

171. Leake CJ. The vector competence of colonized *Aedes (Stegomyia) katherinensis* for Dengue-2 virus. *Transactions of the Royal Society of Tropical Medicine and Hygiene* 1984, **78**(6)**:** 829-832.

172. Ledermann JP, Guillaumot L, Yug L, Saweyog SC, Tided M, Machieng P*, et al. Aedes hensilli* as a potential vector of Chikungunya and Zika viruses. *PLoS neglected tropical diseases* 2014,

8(10).

173. Ledesma N, Harrington L. Mosquito vectors of dog heartworm in the United States: vector status and factors influencing transmission efficiency. *Top Companion Anim M* 2011, **26**(4)**:** 178-185.

174. Li CX, Guo XX, Deng YQ, Xing D, Sun AJ, Liu QM*, et al.* Vector competence and transovarial transmission of two *Aedes aegypti* strains to Zika virus. *Emerging microbes & infections* 2017, **6**.

175. Li KJ, Cai SX, Lin W, Xia J, Pi Q, Hu LQ*, et al.* Impact of malaria vector control interventions at the beginning of a malaria elimination stage in a dominant area of *Anopheles anthropophagus*, Hubei province, China. *Journal of Parasitology* 2015, **101**(5)**:** 598-602.

176. Li YY, Floate KD, Fields PG, Pang BP. Review of treatment methods to remove *Wolbachia* bacteria from arthropods. *Symbiosis* 2014, **62**(1)**:** 1-15.

177. Liang JT, Cheng B, Zhu GD, Wei Y, Tang JX, Cao J*, et al.* Structural divergence of chromosomes between malaria vectors Anopheles lesteri and Anopheles sinensis. *Parasites & vectors* 2016, **9**.

178. Linton YM, Dusfour I, Howard TM, Ruiz F, Nguyen DM, Trung HD*, et al. Anopheles (Cellia) epiroticus* (Diptera: Culicidae), a new malaria vector species in the Southeast Asian Sundaicus Complex. *Bulletin of Entomological Research* 2005, **95**(4)**:** 329-339.

179. Liu H, Lu HJ, Liu ZJ, Jing J, Ren JQ, Liu YY*, et al.* Japanese encephalitis virus in mosquitoes and swine in Yunnan province, China 2009-2010. *Vector-Borne Zoonot* 2013, **13**(1)**:** 41-49.

180. Liu ZZ, Zhou TF, Lai ZT, Zhang ZH, Jia ZR, Zhou GF*, et al.* Competence of *Aedes aegypti*, *Ae. albopictus*, and *Culex quinquefasciatus* mosquitoes as Zika virus vectors, China. *Emerging infectious diseases* 2017, **23**(7)**:** 1085-1091.

181. Lord JS, Gurley ES, Pulliam JRC. Rethinking Japanese encephalitis virus transmission: A framework for implicating host and vector species. *PLoS neglected tropical diseases* 2015, **9**(12).

182. Lourencodeoliveira R, Decastro FA. Culex-Saltanensis Dyar, 1928 - Natural vector of *Plasmodium juxtanucleare* in Rio-De-Janeiro, Brazil. *Memorias do Instituto Oswaldo Cruz* 1991, **86**(1)**:** 87-94.

183. Lourenco-de-Oliveira R, Failloux AB. High risk for chikungunya virus to initiate an enzootic sylvatic cycle in the tropical Americas. *PLoS neglected tropical diseases* 2017, **11**(6).

184. Lubelczyk C, Elias SP, Kantar L, Albert J, Hansen S, Saxton-Shaw K*, et al.* Detection of Eastern

Equine Encephalitis virus antibodies in Moose (Alces americana), Maine, 2010. *Vector-Borne Zoonot* 2014, **14**(1)**:** 77-81.

185. Lutomiah J, Bast J, Clark J, Richardson J, Yalwala S, Oullo D*, et al.* Abundance, diversity, and distribution of mosquito vectors in selected ecological regions of Kenya: public health implications. *Journal of Vector Ecology* 2013, **38**(1)**:** 134-142.

186. Lyimo IN, Ferguson HM. Ecological and evolutionary determinants of host species choice in mosquito vectors. *Trends Parasitol* 2009, **25**(4)**:** 189-196.

187. Maeno Y, Quang NT, Culleton R, Kawai S, Masuda G, Nakazawa S*, et al.* Humans frequently exposed to a range of non-human primate malaria parasite species through the bites of *Anopheles dirus* mosquitoes in South-central Vietnam. *Parasites & vectors* 2015, **8**.

188. Maghsoodi N, Ladonni H, Basseri HR. Species composition and seasonal activities of malaria vectors in an area at reintroduction prevention stage, Khuzestan, South-Western Iran. *Journal of arthropod-borne diseases* 2015, **9**(1)**:** 60-70.

189. Mahardika RW, Ibrahim H, Nurulhusna AH, Awang K. Efficacy of four species of Zingiberaceae extract against vectors of dengue, Chikungunya and filariasis. *Tropical biomedicine* 2017, **34**(2)**:** 375-387.

190. Makanga B, Yangari P, Rahola N, Rougeron V, Elguero E, Boundenga L*, et al.* Ape malaria transmission and potential for ape-to-human transfers in Africa. *Proceedings of the National Academy of Sciences of the United States of America* 2016, **113**(19)**:** 5329-5334.

191. Mancini G, Montarsi F, Calzolari M, Capelli G, Dottori M, Ravagnan S*, et al.* Mosquito species involved in the circulation of West Nile and Usutu viruses in Italy. *Veterinaria italiana* 2017, **53**(2)**:** 97-110.

192. Manrique-Saide P, Escobedo-Ortegon J, Bolio-Gonzalez M, Sauri-Arceo C, Dzib-Florez S, Guillermo-May G*, et al.* Incrimination of the mosquito, *Aedes taeniorhynchus,* as the primary vector of heartworm, *Dirofilaria immitis*, in coastal Yucatan, Mexico. *Medical and Veterinary Entomology* 2010, **24**(4)**:** 456-460.

193. Mariappan T, Samuel PP, Thenmozhi V, Paramasivan R, Sharma PK, Biswas AK*, et al.* Entomological investigations into an epidemic of Japanese encephalitis (JE) in northern districts of West Bengal, India (2011-2012). *Indian Journal of Medical Research* 2014, **139:** 754-761.

194. Martinez-de la Puente J, Moreno-Indias I, Hernandez-Castellano LE, Arguello A, Ruiz S, Soriguer R*, et al.* Host-feeding pattern of *Culex theileri* (Diptera: Culicidae), potential vector of *Dirofilaria immitis* in the Canary Islands, Spain. *Journal of Medical Entomology* 2012, **49**(6)**:** 1419-1423.

195. Matheson R, Boyd MF, Stratman-Thomas WK. Anopheles walkeri, Theobald, as a vector or plasmodium vivax, Grassi and Feletti. *Am J Hyg* 1933, **17**(2)**:** 515-516.

196. Mcintosh BM, Sweetnam J, Mcgillivray GM, Desousa J. Laboratory Transmission of Chikungunya virus by *Mansonia* (Mansonioides) Africana (Theobald). *Ann Trop Med Parasit* 1965, **59**(4)**:** 390-+.

197. Mcintosh BM, Weinbren MP, Kokernot RH, Worth CB. Isolation of viruses from mosquitoes collected at Lumbo, Mozambique .3. Isolation of spondweni virus from *Aedes (Ochlerotatus) Fryeri* (Theobald) and/or *Aedes (Aedimorphus) fowleri* (Demmerez De Charmoy). *American Journal of Tropical Medicine and Hygiene* 1962, **11**(5)**:** 685-&.

198. Mckay T, Bianco T, Rhodes L, Barnett S. Prevalence of *Dirofilaria immitis* (Nematoda: Filarioidea) in mosquitoes from Northeast Arkansas, the United States. *Journal of Medical Entomology* 2013, **50**(4)**:** 871-878.

199. Medlock JM, Snow KR, Leach S. Potential transmission of West Nile virus in the British Isles: an ecological review of candidate mosquito bridge vectors. *Medical and Veterinary Entomology* 2005, **19**(1)**:** 2-21.

200. Mogi M. Unusual life history traits of *Aedes (Stegomyia)* mosquitoes (Diptera: Culicidae) inhabiting *Nepenthes pitchers*. *Ann Entomol Soc Am* 2010, **103**(4)**:** 618-624.

201. Molaei G, andreadis TA, Armstrong PM, anderson JF, Vossbrinck CR. Host feeding patterns of *Culex* mosquitoes and West Nile virus transmission, northeastern United States. *Emerging infectious diseases* 2006, **12**(3)**:** 468-474.

202. Molaei G, andreadis TG, Armstrong PM, Diuk-Wasser M. Host-feeding patterns of potential mosquito vectors in Connecticut, USA: Molecular analysis of bloodmeals from 23 species of *Aedes*, *Anopheles*, *Culex*, *Coquillettidia*, *Psorophora*, and *Uranotaenia*. *Journal of Medical Entomology* 2008, **45**(6)**:** 1143-1151.

203. Molaei G, andreadis TG, Armstrong PM, Thomas MC, Deschamps T, Cuebas-Incle E*, et al.* Vector-host interactions and epizootiology of Eastern Equine Encephalitis virus in Massachusetts. *Vector-Borne Zoonot* 2013, **13**(5)**:** 312-323.

204. Molaei G, Cummings RF, Su TY, Armstrong PM, Williams GA, Cheng ML*, et al.* Vector-host interactions governing epidemiology of West Nile virus in Southern California. *American Journal of Tropical Medicine and Hygiene* 2010, **83**(6)**:** 1269-1282.

205. Mondet B. Importance of Aedes (Diceromyia) furcifer Edwards, 1913 (Diptera: Culicidae) among the arbovirus potential vectors, in the epidemiology of the yellow fever, in the sub-Sudanese savannas of Ivory Coast. *Ann Soc Entomol Fr* 1997, **33**(1)**:** 47-54.

206. Montoya-Lerma J, Solarte YA, Giraldo-Calderon GI, Quinones ML, Ruiz-Lopez F, Wilkerson RC*, et al.* Malaria vector species in Colombia - A review. *Memorias do Instituto Oswaldo Cruz* 2011, **106:** 223-238.

207. Morales-Betoulle ME, Komar N, Panella NA, Alvarez D, Lopez MR, Betoulle JL*, et al.* West Nile virus ecology in a tropical ecosystem in Guatemala. *American Journal of Tropical Medicine and Hygiene* 2013, **88**(1)**:** 116-126.

208. Moreno ES, Rocco IM, Bergo ES, Brasil RA, Siciliano MM, Suzuki A*, et al.* Reemergence of yellow fever: detection of transmission in the State of Sao Paulo, Brazil, 2008. *Revista da Sociedade Brasileira de Medicina Tropical* 2011, **44**(3)**:** 290-296.

209. Moreno-Madrinan MJ, Turell M. Factors of concern regarding Zika and other *Aedes aegypti*-transmitted viruses in the United States. *Journal of Medical Entomology* 2017, **54**(2)**:** 251-257.

210. Morgante O, Shemanchuk JA. Virus of California Encephalitis complex - Isolation from *Culiseta inornata*. *Science* 1967, **157**(3789)**:** 692-+.

211. Mourya DT, Mishra AC, Soman RS. Transmission of Japanese Encephalitis-virus in *Culex pseudovishnui* and *C tritaeniorhynchus* mosquitos. *Indian J Med Res-A* 1991, **93:** 250-252.

212. Muenworn V, Sungvornyothin S, Kongmee M, Polsomboon S, Bangs MJ, Akrathanakul P*, et al.* Biting activity and host preference of the malaria vectors *Anopheles maculatus* and *Anopheles sawadwongporni* (Diptera: Culicidae) in Thailand. *Journal of Vector Ecology* 2009, **34**(1)**:** 62-69.

213. Mutebi JP, Crabtree MB, Kading RC, Powers AM, Lutwama JJ, Miller BR. Mosquitoes of Western Uganda. *Journal of Medical Entomology* 2012, **49**(6)**:** 1289-1306.

214. Mutebi JP, Swope BN, Doyle MS, Biggerstaff BJ. Vector competence of *Culex restuans* (Diptera: Culicidae) from two regions of Chicago with low and high prevalence of West Nile virus human infections. *Journal of Medical Entomology* 2012, **49**(3)**:** 678-686.

215. Muturi EJ, Bara JJ, Rooney AP, Hansen AK. Midgut fungal and bacterial microbiota of *Aedes triseriatus* and *Aedes japonicus* shift in response to La Crosse virus infection. *Molecular ecology* 2016, **25**(16)**:** 4075-4090.

216. Mwangangi JM, Mbogo CM, Orindi BO, Muturi EJ, Midega JT, Nzovu J*, et al.* Shifts in malaria vector species composition and transmission dynamics along the Kenyan coast over the past 20 years. *Malaria journal* 2013, **12**.

217. Nasar F, Haddow AD, Tesh RB, Weaver SC. Eilat virus displays a narrow mosquito vector range. *Parasites & vectors* 2014, **7**.

218. Ndiath MO, Sarr JB, Gaayeb L, Mazenot C, Sougoufara S, Konate L*, et al.* Low and seasonal malaria transmission in the middle Senegal River basin: identification and characteristics of Anopheles vectors. *Parasites & vectors* 2012, **5**.

219. Ndiaye E, Fall G, Gaye A, Bob NS, Talla C, Diagne CT*, et al.* Vector competence of *Aedes vexans* (Meigen), *Culex poicilipes* (Theobald) and *Cx. quinquefasciatus* Say from Senegal for West and East African lineages of Rift Valley fever virus. *Parasites & vectors* 2016, **9**.

220. Ndoen E, Wild C, Dale P, Sipe N, Dale M. Relationships between anopheline mosquitoes and topography in West Timor and Java, Indonesia. *Malaria journal* 2010, **9**.

221. Nelms BM, Thiemann TC, Bridges DN, Williams AE, Koschik ML, Ryan BM*, et al.* Bionomics and vector potential of *Culex thriambus* (Diptera: Culicidae) mosquitoes in Lake county, California. *Journal of Medical Entomology* 2016, **53**(6)**:** 1473-1481.

222. Nepomichene TNJJ, Tata E, Boyer S. Malaria case in Madagascar, probable implication of a new vector, *Anopheles coustani*. *Malaria journal* 2015, **14**.

223. Ngo CT, Dubois G, Sinou V, Parzy D, Le HQ, Harbach RE*, et al.* Diversity of *Anopheles* mosquitoes in Binh Phuoc and Dak Nong provinces of Vietnam and their relation to disease. *Parasites & vectors* 2014, **7**.

224. Nugapola NWNP, De Silva WAPP, Karunaratne SHPP. Distribution and phylogeny of *Wolbachia* strains in wild mosquito populations in Sri Lanka. *Parasites & vectors* 2017, **10**.

225. Obsomer V, Defourny P, Coosemans M. The *Anopheles dirus* complex: spatial distribution and environmental drivers. *Malaria journal* 2007, **6**.

226. Ogden NH, Lindsay LR. Effects of climate and climate change on vectors and vector-borne diseases: Ticks Are Different. *Trends Parasitol* 2016, **32**(8)**:** 646-656.

227. Ogoma SB, Lweitoijera DW, Ngonyani H, Furer B, Russell TL, Mukabana WR*, et al.* Screening mosquito house entry points as a potential method for integrated control of endophagic filariasis, arbovirus and malaria vectors. *PLoS neglected tropical diseases* 2010, **4**(8).

228. Olanga EA, Okombo L, Irungu LW, Mukabana WR. Parasites and vectors of malaria on Rusinga Island, Western Kenya. *Parasites & vectors* 2015, **8**.

229. Olano VA, Matiz MI, Lenhart A, Cabezas L, Vargas SL, Jaramillo JF*, et al.* Schools as potential risk sites for vector-borne disease transmission: Mosquito vectors in rural schools in two municipalities in Colombia. *Journal of the American Mosquito Control Association* 2015, **31**(3)**:** 212-222.

230. Orfano AS, Duarte APM, Molina-Cruz A, Pimenta PF, Barillas-Mury C. *Plasmodium yoelii*

nigeriensis (N67) is a robust animal model to study malaria transmission by South American Anopheline mosquitoes. *PLoS One* 2016, **11**(12).

231. Orshan L, Bin H, Schnur H, Kaufman A, Valinsky A, Shulman L*, et al.* Mosquito vectors of West Nile fever in Israel. *Journal of Medical Entomology* 2008, **45**(5)**:** 939-947.

232. Ortiz DI, Anishchenko M, Weaver SC. Susceptibility of *Psorophora confinnis* (Diptera: Culicidae) to infection with epizootic (subtype IC) and enzootic (subtype ID) Venezuelan equine encephalitis viruses. *Journal of Medical Entomology* 2005, **42**(5)**:** 857-863.

233. Osorio HC, Ze-Ze L, Alves MJ. Host-Feeding Patterns of *Culex pipiens* and other potential mosquito vectors (Diptera: Culicidae) of West Nile virus (Flaviviridae) collected in Portugal. *Journal of Medical Entomology* 2012, **49**(3)**:** 717-721.

234. Papa A, Papadopoulou E, Paliwal R, Kalaitzopoulou S, Mourelatos S, Niedrig M. Insect-specific flaviviruses in *Aedes* mosquitoes in Greece. *Archives of virology* 2016, **161**(8)**:** 2183-2188.

235. Paras KL, O'Brien VA, Reiskind MH. Comparison of the vector potential of different mosquito species for the transmission of heartworm, *Dirofilaria immitis*, in rural and urban areas in and surrounding Stillwater, Oklahoma, USA. *Medical and Veterinary Entomology* 2014, **28:** 60-67.

236. Parida SK, Hazra RK, Marai N, Tripathy HK, Mahapatra N. Host feeding patterns of malaria vectors of Orissa, India. *Journal of the American Mosquito Control Association* 2006, **22**(4)**:** 629-634.

237. Parra-Henao G, Suarez L. Mosquitoes (Diptera: Culiciadae) as potential vectors of arbovirused in the Uraba region, Northwest of Colombia. *Biomedica* 2012, **32**(2)**:** 252-262.

238. Paulson SL, Poirier SJ, Grimstad PR, Craig GB. Vector competence of *Aedes hendersoni* (Diptera, Culicidae) for La-Crosse virus - Lack of impaired function in virus-infected salivary-glands and enhanced virus transmission by sporozoite-infected mosquitos. *Journal of Medical Entomology* 1992, **29**(3)**:** 483-488.

239. Paupy C, Makanga B, Ollomo B, Rahola N, Durand P, Magnus J*, et al. Anopheles moucheti* and *Anopheles vinckei* are candidate vectors of ape *Plasmodium* parasites, including *Plasmodium praefalciparum* in Gabon. *PLoS One* 2013, **8**(2).

240. Pauvolid-Correa A, Solberg O, Couto-Lima D, Kenney J, Serra-Freire N, Brault A*, et al.* Nhumirim virus, a novel flavivirus isolated from mosquitoes from the Pantanal, Brazil. *Archives of virology* 2015, **160**(1)**:** 21-27.

241. Peiris JSM, Amerasinghe FP, Amerasinghe PH, Ratnayake CB, Karunaratne SHPP, Tsai TF. Japanese encephalitis in Sri-Lanka - the study of an epidemic - Vector incrimination, Porcine infection and human-disease. *Transactions of the Royal Society of Tropical Medicine and*

Hygiene 1992, **86**(3)**:** 307-313.

242. Peiris JSM, Amerasinghe PH, Amerasinghe FP, Calisher CH, Perera LP, Arunagiri CK*, et al.* Viruses isolated from mosquitos collected in Sri-Lanka. *American Journal of Tropical Medicine and Hygiene* 1994, **51**(2)**:** 154-161.

243. Pereira LPLA, Brito MCA, Araruna F, de andrade MS, Moraes DFC, Borges ACR*, et al.* Molecular studies with *Aedes (Stegomyia) aegypti* (Linnaeus, 1762), mosquito transmitting the dengue virus. *Parasitology research* 2017, **116**(8)**:** 2057-2063.

244. Perera MDB, Hemingway J, Karunaratne SHPP. Multiple insecticide resistance mechanisms involving metabolic changes and insensitive target sites selected in anopheline vectors of malaria in Sri Lanka. *Malaria journal* 2008, **7**.

245. Phunngam P, Chareonviriyaphap T, Bangs MJ, Arunyawat U. Phylogenetic relationships among malaria vectors and closely related species in Thailand using multilocus DNA sequences. *Journal of the American Mosquito Control Association* 2017, **33**(2)**:** 91-102.

246. Pitzer JB, Byford RL, Vuong HB, Steiner RL, Creamer RJ, Caccamise DF. Potential vectors of West Nile virus in a semiarid environment: Dona Ana county, New Mexico. *Journal of Medical Entomology* 2009, **46**(6)**:** 1474-1482.

247. Port GR, Wilkes TJ. *Aedes (Diceromyia) furcifer*-Taylori and a yellow-fever outbreak in the Gambia. *Transactions of the Royal Society of Tropical Medicine and Hygiene* 1979, **73**(3)**:** 341-344.

248. Port GR, Wilkes TJ, Bryan JH. A note on the distribution of *Aedes (Diceromyia) furcifer*-Taylori, a yellow-fever vector, in the Gambia. *Insect Sci Appl* 1981, **2**(3)**:** 163-166.

249. Pothikasikorn J, Bangs MJ, Boonplueang R, Chareonviriyaphap T. Susceptibility of various mosquitoes of Thailand to nocturnal subperiodic *Wuchereria bancrofti*. *Journal of Vector Ecology* 2008, **33**(2)**:** 313-320.

250. Pradel JA, Martin T, Rey D, Foussadier R, Bicout DJ. Is *Culex modestus* (Diptera: Culicidae), vector of West Nile virus, spreading in the Dombes area, France? *Journal of Medical Entomology* 2009, **46**(6)**:** 1269-1281.

251. Prakash A, Bhattacharyya DR, Mohapatra PK, Mahanta J. Malaria transmission risk by the mosquito *Anopheles baimaii* (formerly known as *An. dirus* species D) at different hours of the night in North-east India. *Medical and Veterinary Entomology* 2005, **19**(4)**:** 423-427.

252. Pramanik MK, Aditya G. Immatures of *Lutzia fuscana* (Wiedemann, 1820) (Diptera: Culicidae) in ricefields: implications for biological control of vector mosquitoes. *Asian Pacific journal of tropical medicine* 2009, **2**(3)**:** 29-34.

253. Pridgeon JW, Pereira RM, Becnel JJ, Allan SA, Clark GG, Linthicum KJ. Susceptibility of *Aedes aegypti*, *Culex quinquefasciatus* say, and *Anopheles quadrimaculatus* say to 19 pesticides with different modes of action. *Journal of Medical Entomology* 2008, **45**(1)**:** 82-87.

254. Prow NA. The changing epidemiology of Kunjin virus in Australia. *International journal of environmental research and public health* 2013, **10**(12)**:** 6255-6272.

255. Ramasamy MS, Kulasekera R, Wanniarachchi IC, Srikrishnaraj KA, Ramasamy R. Interactions of human malaria parasites, *Plasmodium vivax* and *P-falciparum*, with the midgut of *Anopheles* mosquitoes. *Medical and Veterinary Entomology* 1997, **11**(3)**:** 290-296.

256. Ramasamy R, Dealwis R, Wijesundere A, Ramasamy MS. Malaria transmission at a new irrigation project in Sri-Lanka - the emergence of *Anopheles annularis* as a major vector. *American Journal of Tropical Medicine and Hygiene* 1992, **47**(5)**:** 547-553.

257. Rana SM, Khan EA, Yaqoob A, Latif AA, Abbasi MM. Susceptibility and irritability of adult forms of main malaria vectors against insecticides used in the indoor residual sprays in Muzaffargarh District, Pakistan: A Field Survey. *Journal of Medical Entomology* 2014, **51**(2)**:** 387-391.

258. Reisen WK, Fang Y, Martinez VM. Vector competence of *Culiseta incidens* and *Culex thriambus* for West Nile virus. *Journal of the American Mosquito Control Association* 2006, **22**(4)**:** 662-665.

259. Ren ZP, Wang DQ, Ma AM, Hwang J, Bennett A, Sturrock HJW*, et al.* Predicting malaria vector distribution under climate change scenarios in China: Challenges for malaria elimination. *Scientific Reports* 2016, **6**.

260. Reuben R. Re – *Anopheles nigerrimus* as a vector of malaria in India. *Journal of the American Mosquito Control Association* 1991, **7**(1)**:** 132-132.

261. Ribeiro AF, Urbinatti PR, Duarte AMRD, de Paula MB, Pereira DM, Mucci LF*, et al.* Mosquitoes in degraded and preserved areas of the Atlantic forest and potential for vector-borne disease risk in the municipality of Sao Paulo, Brazil. *Journal of Vector Ecology* 2012, **37**(2)**:** 316-324.

262. Richard V, Paoaafaite T, Cao-Lormeau V. Acquittal of *Culex quinquefasciatus* in transmitting Zika virus during the French Polynesian outbreak. *Acta tropica* 2017, **173:** 200-201.

263. Richards SL, anderson SL, Lord CC, Tabachnick WJ. Impact of West Nile virus dose and incubation period on vector competence of *Culex nigripalpus* (Diptera: Culicidae). *Vector-Borne Zoonot* 2011, **11**(11)**:** 1487-1491.

264. Robert V, Lhuillier M, Meunier D, Sarthou JL, Monteny N, Digoutte JP*, et al.* Yellow fever, dengue-2 and other mosquito-borne arboviruses, in Burkina-Faso, from 1983 to 1986 -

entomological and epidemiologic aspects. *Bulletin de la Societe de pathologie exotique* 1993, **86**(2)**:** 90-100.

265. Rodriguez MH. Malaria and dengue vector biology and control in Latin America. *Wag Ur Fron* 2006, **11:** 129-141.

266. Romi R, Pontuale G, Ciufolini G, Fiorentini G, Marchi A, Nicoletti L*, et al.* Potential vectors of West Nile virus following an equine disease outbreak in Italy. *Medical and Veterinary Entomology* 2004, **18**(1)**:** 14-19.

267. Roundy CM, Azar SR, Rossi SL, Huang JH, Leal G, Yun R*, et al.* Variation in *Aedes aegypti* mosquito competence for Zika virus transmission. *Emerging infectious diseases* 2017, **23**(4)**:** 625-632.

268. Rowland M, Mohammed N, Rehman H, Hewitt S, Mendis C, Ahmad M*, et al.* Anopheline vectors and malaria transmission in eastern Afghanistan. *Transactions of the Royal Society of Tropical Medicine and Hygiene* 2002, **96**(6)**:** 620-626.

269. Ruangsittichai J, Apiwathnasorn C, Dujardin JP. Interspecific and sexual shape variation in the filariasis vectors *Mansonia* dives and Ma. bonneae. *Infection Genetics and Evolution* 2011, **11**(8)**:** 2089-2094.

270. Rudnick A, Sather GE, Hammon WM. A Strain of Sindbis virus Isolated from *Culex bitaeniorhynchus* mosquitoes in Philippines. *American Journal of Tropical Medicine and Hygiene* 1962, **11**(4)**:** 546-&.

271. Russell TL, Beebe NW, Cooper RD, Lobo NF, Burkot TR. Successful malaria elimination strategies require interventions that target changing vector behaviours. *Malaria journal* 2013, **12**.

272. Ryan PA, Kay BH. Vector competence of mosquitoes (Diptera: Culicidae) from Maroochy Shire, Australia, for Barmah Forest virus. *Journal of Medical Entomology* 1999, **36**(6)**:** 856-860.

273. Saeung A, Hempolchom C, Baimai V, Thongsahuan S, Taai K, Jariyapan N*, et al.* Susceptibility of eight species members in the *Anopheles hyrcanus* group to nocturnally subperiodic Brugia malayi. *Parasites & vectors* 2013, **6**.

274. Sahu SS, Gunasekaran K, Krishnamoorthy N, Vanamail P, Mathivanan A, Manonmani A*, et al.* Bionomics of *Anopheles fluviatilis* and *Anopheles culicifacies* (Diptera: Culicidae) in Relation to Malaria Transmission in East-Central India. *Journal of Medical Entomology* 2017, **54**(4)**:** 821-830.

275. Saiyasombat R, Dorman KS, Garcia-Rejon JE, Lorono-Pino MA, Farfan-Ale JA, Blitvich BJ. Isolation and sequence analysis of *Culex flavivirus* from *Culex interrogator* and *Culex*

quinquefasciatus in the Yucatan Peninsula of Mexico. *Archives of virology* 2010, **155**(6)**:** 983-986.

276. Sang R, Kioko E, Lutomiah J, Warigia M, Ochieng C, O'Guinn M*, et al.* Rift Valley fever virus epidemic in Kenya, 2006/2007: The entomologic investigations. *American Journal of Tropical Medicine and Hygiene* 2010, **83**(2)**:** 28-37.

277. Sang R, Lutomiah J, Said M, Makio A, Koka H, Koskei E*, et al.* Effects of irrigation and rainfall on the population dynamics of Rift Valley fever and other arbovirus mosquito vectors in the epidemic-prone Tana River county, Kenya. *Journal of Medical Entomology* 2017, **54**(2)**:** 460-470.

278. Sardelis MR, Turell MJ, andre ARG. Laboratory transmission of La Crosse virus by *Ochlerotatus j. japonicus* (Diptera: Culicidae). *Journal of Medical Entomology* 2002, **39**(4)**:** 635-639.

279. Sardelis MR, Turell MJ, Dohm DJ, O'Guinn ML. Vector competence of selected North American *Culex* and *Coquillettidia* mosquitoes for West Nile virus. *Emerging infectious diseases* 2001, **7**(6)**:** 1018-1022.

280. Savage HM, Aggarwal D, Apperson CS, Katholi CR, Gordon E, Hassan HK*, et al.* Host choice and West Nile virus infection rates in blood-fed mosquitoes, including members of the *Culex pipiens* complex, from Memphis and Shelby county, Tennessee, 2002-2003. *Vector-Borne Zoonot* 2007, **7**(3)**:** 365-386.

281. Savage HM, Fritz CL, Rutstein D, Yolwa A, Vorndam V, Gubler DJ. Epidemic of dengue-4 virus in Yap State, Federated States of Micronesia, and implication of *Aedes hensilli* as an epidemic vector. *American Journal of Tropical Medicine and Hygiene* 1998, **58**(4)**:** 519-524.

282. Sebesta O, Gelbic I, Pesko J. Daily and seasonal variation in the activity of potential vector mosquitoes. *Cent Eur J Biol* 2011, **6**(3)**:** 422-430.

283. Semuel S, Ivon A, Melda SS, Yustinus M, Mardi RP, Jan L*, et al.* The malaria vectors of *Anopheles flavirostris* and *Anopheles barbirostris* in West Southeast Maluku Regency. *Tropical biomedicine* 2016, **33**(4)**:** 771-779.

284. Seo HJ, Kim HC, Klein TA, Ramey AM, Lee JH, Kyung SG*, et al.* Molecular detection and genotyping of Japanese encephalitis virus in mosquitoes during a 2010 outbreak in the Republic of Korea. *PLoS One* 2013, **8**(2).

285. Serra OP, Cardoso BF, Ribeiro ALM, dos Santos FAL, Slhessarenko RD. Mayaro virus and dengue virus 1 and 4 natural infection in culicids from Cuiaba, state of Mato Grosso, Brazil. *Memorias do Instituto Oswaldo Cruz* 2016, **111**(1)**:** 20-29.

286. Shapiro LLM, Murdock CC, Jacobs GR, Thomas RJ, Thomas MB. Larval food quantity affects the capacity of adult mosquitoes to transmit human malaria. *Proceedings of the Royal Society B-Biological Sciences* 2016, **283**(1834).

287. Shen DT, Gorham JR, Harwood RF, Padgett GA. Persistence of Aleutian disease virus in mosquito *Aedes fitchii*. *Arch Ges Virusforsch* 1973, **40**(3-4)**:** 375-381.

288. Shepard JJ, andreadis TG, Thomas MC, Molaei G. Host associations of mosquitoes at eastern equine encephalitis virus foci in Connecticut, USA. *Parasites & vectors* 2016, **9**.

289. Shragai T, Tesla B, Murdock C, Harrington LC. Zika and chikungunya: mosquito-borne viruses in a changing world. *Annals of the New York Academy of Sciences* 2017, **1399**(1)**:** 61-77.

290. Shriram AN, Krishnamoorthy K, Vijayachari P. Diurnally subperiodic filariasis among the Nicobarese of Nicobar district - epidemiology, vector dynamics & prospects of elimination. *Indian Journal of Medical Research* 2015, **141:** 598-607.

291. Siju KP, Reifenrath A, Scheiblich H, Neupert S, Predel R, Hansson BS*, et al.* Neuropeptides in the antennal lobe of the yellow fever mosquito, *Aedes aegypti*. *J Comp Neurol* 2014, **522**(3)**:** 592-608.

292. Singh P, Lingala MAL, Sarkar S, Dhiman RC. Mapping of malaria vectors at district level in India: Changing scenario and identified gaps. *Vector-Borne Zoonot* 2017, **17**(2)**:** 91-98.

293. Singhasivanon P. Malaria vectors in the greater Mekong Subregion: Overview of malaria vectors and remaining challenges. *Se Asian J Trop Med* 2013, **44:** 73-165.

294. Sinka ME, Bangs MJ, Manguin S, Coetzee M, Mbogo CM, Hemingway J*, et al.* The dominant *Anopheles* vectors of human malaria in Africa, Europe and the Middle East: occurrence data, distribution maps and bionomic precis. *Parasites & vectors* 2010, **3**.

295. Sotomayor-Bonilla J, Abella-Medrano CA, Chaves A, Alvarez-Mendizabal P, Rico-Chavez O, Ibanez-Bernal S*, et al.* Potential sympatric vectors and mammalian hosts of Venezuelan Equine Encephalitis virus in Southern Mexico. *Journal of wildlife diseases* 2017, **53**(3)**:** 657-661.

296. Southgate BA, Bryan JH. Factors Affecting Transmission of Wuchereria-Bancrofti by Anopheline mosquitos .4. Facilitation, limitation, proportionality and their epidemiologic significance. *Transactions of the Royal Society of Tropical Medicine and Hygiene* 1992, **86**(5)**:** 523-530.

297. Srinivasan R, Viswam K. Laboratory Studies on the biology of *Mansonia annulifer* a Theobald (1901) (Diptera, Culicidae). *Indian Journal of Medical Research* 1986, **83:** 384-386.

298. Sriwichai P, Samung Y, Sumruayphol S, Kiattibutr K, Kumpitak C, Payakkapol A*, et al.* Natural

human *Plasmodium* infections in major *Anopheles* mosquitoes in western Thailand. *Parasites & vectors* 2016, **9**.

299. Stephen C, Plamondon N, Belton P. Notes on the distribution of mosquito species that could potentially transmit West Nile virus on Vancouver Island, British Columbia. *Journal of the American Mosquito Control Association* 2006, **22**(3)**:** 553-556.

300. Su CL, Yang CF, Teng HJ, Lu LC, Lin C, Tsai KH*, et al.* Molecular Epidemiology of Japanese encephalitis virus in mosquitoes in Taiwan during 2005-2012. *PLoS neglected tropical diseases* 2014, **8**(10).

301. Sudeep AB, Ghodke YS, George RP, Ingale VS, Dhaigude SD, Gokhale MD. Vectorial capacity of *Culex gelidus* (Theobald) mosquitoes to certain viruses of public health importance in India. *Journal of vector borne diseases* 2015, **52**(2)**:** 153-158.

302. Sugiarto, Hadi UK, Soviana S, Hakim L. Confirmation of *Anopheles peditaeniatus* and *Anopheles sundaicus* as malaria vectors (Diptera: Culicidae) in Sungai Nyamuk village, Sebatik Island North Kalimantan, Indonesia using an enzyme-linked immunosorbent Assay. *Journal of Medical Entomology* 2016, **53**(6)**:** 1422-1424.

303. Sun JF, Wu D, Zhou HQ, Zhang H, Guan DW, He X*, et al.* The epidemiological characteristics and genetic diversity of dengue virus during the third largest historical outbreak of dengue in Guangdong, China, in 2014. *J Infection* 2016, **72**(1)**:** 80-90.

304. Szentpali-Gavaller K, Antal L, Toth M, Kemenesi G, Soltesz Z, Dan A*, et al.* Monitoring of West Nile virus in mosquitoes between 2011-2012 in Hungary. *Vector-Borne Zoonot* 2014, **14**(9)**:** 648-655.

305. Taai K, Harbach RE, Aupalee K, Srisuka W, Yasanga T, Otsuka Y*, et al.* An effective method for the identification and separation of *Anopheles minimus*, the primary malaria vector in Thailand, and its sister species *Anopheles harrisoni*, with a comparison of their mating behaviors. *Parasites & vectors* 2017, **10**.

306. Tainchum K, Ritthison W, Chuaycharoensuk T, Bangs MJ, Manguin S, Chareonviriyaphap T. Diversity of *Anopheles* species and trophic behavior of putative malaria vectors in two malaria endemic areas of northwestern Thailand. *Journal of Vector Ecology* 2014, **39**(2)**:** 424-436.

307. Tananchai C, Tisgratog R, Juntarajumnong W, Grieco JP, Manguin S, Prabaripai A*, et al.* Species diversity and biting activity of *Anopheles dirus* and *Anopheles baimaii* (Diptera: Culicidae) in a malaria prone area of western Thailand. *Parasites & vectors* 2012, **5**.

308. Tantely ML, Rakotoniaina JC, Tata E, andrianaivolambo L, Razafindrasata F, Fontenille D*, et al.* Biology of mosquitoes that are potential vectors of Rift Valley fever virus in different biotopes of the central highlands of Madagascar. *Journal of Medical Entomology* 2013, **50**(3)**:** 603-610.

309. Tchouassi DP, Sang R, Sole CL, Bastos ADS, Cohnstaedt LW, Torto B. Trapping of Rift Valley fever (RVF) vectors using Light Emitting Diode (LED) CDC traps in two arboviral disease hot spots in Kenya. *Parasites & vectors* 2012, **5**.

310. Telschow A, Grziwotz F, Crain P, Miki T, Mains JW, Sugihara G*, et al.* Infections of *Wolbachia* may destabilize mosquito population dynamics. *Journal of theoretical biology* 2017, **428:** 98-105.

311. Teng HJ, Wu YL, Lin TH. Mosquito fauna in water-holding containers with emphasis on dengue vectors (Diptera: Culicidae) in Chungho, Taipei county, Taiwan. *Journal of Medical Entomology* 1999, **36**(4)**:** 468-472.

312. Thongsahuan S, Baimai V, Junkum A, Saeung A, Min GS, Joshi D*, et al.* Susceptibility of *Anopheles campestris*-like and *Anopheles barbirostris* species complexes to *Plasmodium falciparum* and *Plasmodium vivax* in Thailand. *Memorias do Instituto Oswaldo Cruz* 2011, **106**(1)**:** 105-112.

313. Thongsripong P, Green A, Kittayapong P, Kapan D, Wilcox B, Bennett S. Mosquito vector diversity across habitats in central Thailand endemic for dengue and other arthropod-borne diseases. *PLoS neglected tropical diseases* 2013, **7**(10).

314. Tiawsirisup S, Nuchprayoon S. Mosquito distribution and Japanese encephalitis virus infection in the immigration bird (Asian open-billed stork) nested area in Pathum Thani province, central Thailand. *Parasitology research* 2010, **106**(4)**:** 907-910.

315. Tingstrom O, Lwande OW, Naslund J, Spyckerelle I, Engdahl C, Von Schoenberg P*, et al.* Detection of Sindbis and Inkoo virus RNA in genetically typed mosquito larvae sampled in Northern Sweden. *Vector-Borne Zoonot* 2016, **16**(7)**:** 461-467.

316. Trajer A, Bede-Fazekas A, Bobvos J, Paldy A. Seasonality and geographical occurrence of West Nile fever and distribution of Asian tiger mosquito. *Idojaras* 2014, **118**(1)**:** 19-40.

317. Tuanudom R, Yurayart N, Tiawsirisup S. Effects of Chikungunya virus titers in blood meals on virus infection, dissemination, and transmission in Asian tiger mosquito: *Aedes albopictus* (Diptera: Culicidae). *Thai J Vet Med* 2017, **47**(2)**:** 233-240.

318. Tung KC, Cheng FP, Lai CH, Wang KS, Wang JS, Lee WM. Demonstration of vector competence of *Culex quinquefasciatus* (Diptera: Culicidae) for *Setaria digitata*. *Veterinary parasitology* 2004, **123**(3-4)**:** 279-284.

319. Tuno N, Tsuda Y, Takagi M. How zoophilic Japanese encephalitis vector mosquitoes feed on Humans. *Journal of Medical Entomology* 2017, **54**(1)**:** 8-13.

320. Turell MJ. Vector competence of three Venezuelan mosquitoes (Diptera: Culicidae) for an epizootic IC strain of Venezuelan equine encephalitis virus. *Journal of Medical Entomology* 1999, **36**(4)**:** 407-409.

321. Turell MJ, Barth J, Coleman RE. Potential for Central American mosquitoes to transmit epizootic and enzootic strains of Venezuelan equine encephalitis virus. *Journal of the American Mosquito Control Association* 1999, **15**(3)**:** 295-298.

322. Turell MJ, Britch SC, Aldridge RL, Kline DL, Boohene C, Linthicum KJ. Potential for mosquitoes (Diptera: Culicidae) from Florida to transmit Rift Valley fever virus. *Journal of Medical Entomology* 2013, **50**(5)**:** 1111-1117.

323. Turell MJ, Britch SC, Aldridge RL, Xue RD, Smith ML, Cohnstaedt LW*, et al.* Potential for *Psorophora columbiae* and *Psorophora ciliata* mosquitoes (Diptera: Culicidae) to Transmit Rift Valley fever virus. *Journal of Medical Entomology* 2015, **52**(5)**:** 1111-1116.

324. Turell MJ, Dohm DJ, Fernandez R, Calampa C, O'Guinn ML. Vector competence of peruvian mosquitoes (Diptera: Culicidae) for a subtype IIIC virus in the Venezuelan equine encephalomyelitis complex isolated from mosquitoes captured in Peru. *Journal of the American Mosquito Control Association* 2006, **22**(1)**:** 70-75.

325. Turell MJ, Dohm DJ, Fonseca DM. Comparison of the potential for different genetic forms in the *Culex pipiens* Complex in North America to transmit Rift Valley fever virus. *Journal of the American Mosquito Control Association* 2014, **30**(4)**:** 253-259.

326. Turell MJ, Jones JW, Sardelis MR, Dohm DJ, Coleman RE, Watts DM*, et al.* Vector competence of peruvian mosquitoes (Diptera: culicidae) for epizootic and enzootic strains of Venezuelan equine encephalomyelitis virus. *Journal of Medical Entomology* 2000, **37**(6)**:** 835-839.

327. Turell MJ, Linthicum KJ, Beaman JR. Transmission of Rift-Valley fever virus by adult mosquitos after ingestion of virus as larvae. *American Journal of Tropical Medicine and Hygiene* 1990, **43**(6)**:** 677-680.

328. Turell MJ, Linthicum KJ, Patrican LA, Davies FG, Kairo A, Bailey CL. Vector competence of selected African mosquito (Diptera: Culicidae) species for Rift Valley fever virus. *Journal of Medical Entomology* 2008, **45**(1)**:** 102-108.

329. Turell MJ, O'Guinn ML, Dohm D, Zyzak M, Watts D, Fernandez R*, et al.* Susceptibility of Peruvian mosquitoes to eastern equine encephalitis virus. *Journal of Medical Entomology* 2008, **45**(4)**:** 720-725.

330. Turell MJ, O'Guinn ML, Dohm DJ, Jones JW. Vector competence of North American mosquitoes (Diptera: Culicidae) for West Nile virus. *Journal of Medical Entomology* 2001, **38**(2)**:**

130-134.

331. Turell MJ, O'Guinn ML, Navarro R, Romero G, Estrada-Franco JG. Vector competence of Mexican and Honduran mosquitoes (Diptera: culicidae) for enzootic (IE) and epizootic (IC) strains of Venezuelan equine encephalomyelitis virus. *Journal of Medical Entomology* 2003, **40**(3)**:** 306-310.

332. Turell MJ, Presley SM, Gad AM, Cope SE, Dohm DJ, Morrill JC*, et al.* Vector competence of Egyptian mosquitoes for Rift Valley fever virus. *American Journal of Tropical Medicine and Hygiene* 1996, **54**(2)**:** 136-139.

333. Turell MJ, Wilson WC, Bennett KE. Potential for North American mosquitoes (Diptera: Culicidae) to transmit Rift Valley fever virus. *Journal of Medical Entomology* 2010, **47**(5)**:** 884-889.

334. Ubalee R, Kim HC, Schuster AL, McCardle PW, Phasomkusolsil S, Takhampunya R*, et al.* Vector competence of *Anopheles kleini* and *Anopheles sinensis* (Diptera: Culicidae) from the Republic of Korea to vivax malaria-infected blood from patients from Thailand. *Journal of Medical Entomology* 2016, **53**(6)**:** 1425-1432.

335. Uejio CK, Kemp A, Comrie AC. Climatic Controls on West Nile virus and Sindbis virus transmission and outbreaks in South Africa. *Vector-Borne Zoonot* 2012, **12**(2)**:** 117-125.

336. Ughasi J, Bekard HE, Coulibaly M, Adabie-Gomez D, Gyapong J, Appawu M*, et al. Mansonia africana* and *Mansonia uniformis* are vectors in the transmission of *Wuchereria bancrofti* lymphatic filariasis in Ghana. *Parasites & vectors* 2012, **5**.

337. Unlu I, Kramer WL, Roy AF, Foil LD. Detection of West Nile virus RNA in mosquitoes and identification of mosquito blood meals collected at alligator farms in Louisiana. *Journal of Medical Entomology* 2010, **47**(4)**:** 625-633.

338. van den Hurk AF, Hall-Mendelin S, Pyke AT, Smith GA, Mackenzie JS. Vector competence of Australian mosquitoes for Chikungunya virus. *Vector-Borne Zoonot* 2010, **10**(5)**:** 489-495.

339. van den Hurk AF, Hall-Mendelin S, Webb CE, Tan CSE, Frentiu FD, Prow NA*, et al.* Role of enhanced vector transmission of a new West Nile virus strain in an outbreak of equine disease in Australia in 2011. *Parasites & vectors* 2014, **7**.

340. Van Den Hurk AF, Montgomery BL, Northill JA, Smith IL, Zborowski P, Ritchie SA*, et al.* Short report: The first isolation of Japanese encephalitis virus from mosquitoes collected from mainland Australia. *American Journal of Tropical Medicine and Hygiene* 2006, **75**(1)**:** 21-25.

341. Van Den Hurk AF, Nisbet DJ, Foley PN, Ritchie SA, Mackenzie JS, Beebe NW. Isolation of arboviruses from mosquitoes (Diptera: Culicidae) collected from the Gulf Plains region of

northwest Queensland, Australia. *Journal of Medical Entomology* 2002, **39**(5)**:** 786-792.

342. van den Hurk AF, Nisbet DJ, Hall RA, Kay BH, MacKenzie JS, Ritchie SA. Vector competence of Australian mosquitoes (Diptera: Culicidae) for Japanese encephalitis virus. *Journal of Medical Entomology* 2003, **40**(1)**:** 82-90.

343. Varnado W, Goddard J. Abundance and diversity of mosquito species collected from a rural area of Central Mississippi: Implications for West Nile virus transmission in Mississippi. *Journal of the American Mosquito Control Association* 2015, **31**(2)**:** 182-186.

344. Vasconcelos PFC, Costa ZG, da Rossa EST, Luna E, Rodrigues SG, Barros VLRS*, et al.* Epidemic of jungle yell fever in Brazil, 2000: Implications of climatic alterations in disease spread. *J Med Virol* 2001, **65**(3)**:** 598-604.

345. Vasconcelos PFC, Rodrigues SG, Degallier N, Moraes MAP, daRosa JFST, daRosa EST*, et al.* An epidemic of sylvatic yellow fever in the southeast region of Maranhao State, Brazil, 1993-1994: Epidemiologic and entomologic findings. *American Journal of Tropical Medicine and Hygiene* 1997, **57**(2)**:** 132-137.

346. Vazquez A, Ruiz S, Herrero L, Moreno J, Molero F, Magallanes A*, et al.* Short Report: West Nile and Usutu viruses in mosquitoes in Spain, 2008-2009. *American Journal of Tropical Medicine and Hygiene* 2011, **85**(1)**:** 178-181.

347. Vogels CBF, Fros JJ, Goertz GP, Pijlman GP, Koenraadt CJM. Vector competence of northern European *Culex pipiens* biotypes and hybrids for West Nile virus is differentially affected by temperature. *Parasites & vectors* 2016, **9**.

348. Vogels CBF, Goertz GP, Pijlman GP, Koenraadt CJM. Vector competence of northern and Southern European *Culex pipiens pipiens* mosquitoes for West Nile virus across a gradient of temperatures. *Med Vet Entomol* 2017.

349. Vythilingam I. Plasmodium knowlesi in humans: a review on the role of its vectors in Malaysia. *Tropical biomedicine* 2010, **27**(1)**:** 1-12.

350. Vythilingam I, Singh KI, Mahadevan S, Zaridah MS, Ong KK, Abidin MHZ. Studies on Japanese encephalitis vector mosquitos in Selangor, Malaysia. *Journal of the American Mosquito Control Association* 1993, **9**(4)**:** 467-469.

351. Vythilingam I, Tan CH, Asmad M, Chan ST, Lee KS, Singh B. Natural transmission of *Plasmodium knowlesi* to humans by *Anopheles latens* in Sarawak, Malaysia. *Transactions of the Royal Society of Tropical Medicine and Hygiene* 2006, **100**(11)**:** 1087-1088.

352. Wagner VE, Newson HD. Field investigations on *Aedes fitchii* mosquito populations in a woodland pool ecosystem. *Mosq News* 1975, **35**(4)**:** 518-522.

353. Waite JL, Swain S, Lynch PA, Sharma SK, Haque MA, Montgomery J*, et al.* Increasing the potential for malaria elimination by targeting zoophilic vectors. *Scientific Reports* 2017, **7**.

354. Walker ED, Torres EP, Villanueva RT. Components of the vectorial capacity of *Aedes poicilius* for *Wuchereria bancrofti* in Sorsogon province, Philippines. *Ann Trop Med Parasit* 1998, **92**(5)**:** 603-614.

355. Wang X, Tu WC, Huang EJ, Chen YH, Chen JH, Yeh WB. Identification of disease-transmitting mosquitoes: development of species-specific probes for DNA chip assay using mitochondrial COI and ND2 genes and ribosomal internal transcribed spacer 2. *Journal of Medical Entomology* 2017, **54**(2)**:** 396-402.

356. Watson TM, Kay BH. Vector competence of *Aedes notoscriptus* (Diptera: Culicidae) for Barmah forest virus and of this species and *Aedes aegypti* (Diptera: Culicidae) for dengue 1-4 viruses in Queensland, Australia. *Journal of Medical Entomology* 1999, **36**(4)**:** 508-514.

357. Webb CE, Doggett SL, Ritchie SA, Russell RC. Vector competence of three Australian mosquitoes, *Verrallina carmenti*, *Verrallina lineata*, and *Mansonia septempunctata* (Diptera: Culicidae), for Ross River virus. *Journal of Medical Entomology* 2008, **45**(4)**:** 737-740.

358. Weidner E, Canning EU, Rutledge CR, Meek CL. Mosquito (Diptera: Culicidae) host compatibility and vector competency for the human myositic parasite *Trachipleistophora hominis* (*Phylum microspora*). *Journal of Medical Entomology* 1999, **36**(4)**:** 522-525.

359. Weng MH, Lien JC, Ji DD. Monitoring of Japanese encephalitis virus infection in mosquitoes (Diptera: Culicidae) at Guandu Nature Park, Taipei, 2002-2004. *Journal of Medical Entomology* 2005, **42**(6)**:** 1085-1088.

360. White SE, Fukuda T, Undeen AH. Horizontal transmission *of Amblyospora opacita* (Microspora, Amblyosporidae) between the mosquito, *Culex territans*, and the Copepod, *Paracyclops fimbriatus* Chiltoni. *Journal of invertebrate pathology* 1994, **63**(1)**:** 19-25.

361. Wiwatanaratanabutr I. Geographic distribution of wolbachial infections in mosquitoes from Thailand. *Journal of invertebrate pathology* 2013, **114**(3)**:** 337-340.

362. Wonham MJ, Lewis MA, Renclawowicz J, Van den Driessche P. Transmission assumptions generate conflicting predictions in host-vector disease models: a case study in West Nile virus. *Ecol Lett* 2006, **9**(6)**:** 706-725.

363. Wu S, Pan JY, Wang XZ, Zhou SS, Zhang GQ, Liu Q*, et al. Anopheles pseudowillmori* is the predominant malaria vector in Motuo county, Tibet Autonomous Region. *Malaria journal* 2009, **8**.

364. Yang TC, Casati S, Flacio E, Caminada AP, Ruggeri-Bernardi N, Demarta A*, et al.* Detection of Chikungunya virus and arboviruses in mosquito vectors. *J Entomol Sci* 2010, **45**(3)**:** 272-282.

365. Yang TC, Xu XM, Hou J, Gong ZY, Cheng ZP, Fan WZ*, et al.* Dengue fever vector composition and pesticide residues in Yiwu, Zhejiang province, China. *J Entomol Sci* 2012, **47**(4)**:** 309-315.

366. Yapabandara AMGM, Curtis CF. Vectors and malaria transmission in a gem mining area in Sri Lanka. *Journal of Vector Ecology* 2004, **29**(2)**:** 264-276.

367. Yapabandara AMGM, Curtis CF, Wickramasinghe MB, Fernando WP. Control of malaria vectors with the insect growth regulator pyriproxyfen in a gem-mining area in Sri Lanka. *Acta tropica* 2001, **80**(3)**:** 265-276.

368. Yee DA, Allgood D, Kneitel JM, Kuehn KA. Constitutive differences between natural and artificial container mosquito habitats: Vector communities, resources, microorganisms, and habitat parameters. *Journal of Medical Entomology* 2012, **49**(3)**:** 482-491.

369. Yee DA, Skiff JF. Interspecific competition of a new invasive mosquito, *Culex coronator*, and two container mosquitoes, *Aedes albopictus* and *Cx. quinquefasciatus* (Diptera: Culicidae), across different detritus environments. *Journal of Medical Entomology* 2014, **51**(1)**:** 89-96.

370. Yildirim A, Inci A, Duzlu O, Biskin Z, Ica A, Sahin I. *Aedes vexans* and *Culex pipiens* as the potential vectors of *Dirofilaria immitis* in Central Turkey. *Veterinary parasitology* 2011, **178**(1-2)**:** 143-147.

371. Yurayart N, Kaewthamasorn M, Tiawsirisup S. Vector competence of *Aedes albopictus* (Skuse) and *Aedes aegypti* (Linnaeus) for *Plasmodium gallinaceum* infection and transmission. *Veterinary parasitology* 2017, **241:** 20-25.

372. Zarowiecki M, Loaiza JR, Conn JE. Towards a new role for vector systematics in parasite control. *Parasitology* 2011, **138**(13)**:** 1723-1729.

373. Zeller HG, Fontenille D, TraoreLamizana M, Thiongane Y, Digoutte JP. Enzootic activity of Rift Valley fever virus in Senegal. *American Journal of Tropical Medicine and Hygiene* 1997, **56**(3)**:** 265-272.

374. Zhu GD, Xia H, Zhou HY, Li JL, Lu F, Liu YB*, et al.* Susceptibility of *Anopheles sinensis* to *Plasmodium vivax* in malarial outbreak areas of central China. *Parasites & vectors* 2013, **6**.
