# Supplementary material for: Elevated atmospheric CO2 promoted speciation in mosquitoes (Diptera, Culicidae)
Source: Commun Biol. 2018 Nov 5;1:182. doi: 10.1038/s42003-018-0191-7 (PMC6218564; doi:10.1038/s42003-018-0191-7)
Supplement: Supplementary file 2 — Description of Supplementary Data [file 42003_2018_191_MOESM2_ESM.docx]

**Description of Additional Supplementary Files**

**File Name**: Supplementary Data 1

**Description**: Table of the ages of dated nodes used for time-calibration.

**File Name**: Supplementary Data 2

**Description**: Table of vectors and the diseases that they transmit.

**File Name**: Supplementary Data 3

**Description**: Data underpinning our plots of atmospheric CO_2_ concentration through time.
